# Unique DNA Repair Gene Variations and Potential Associations with the Primary Antibody Deficiency Syndromes IgAD and CVID

**DOI:** 10.1371/journal.pone.0012260

**Published:** 2010-08-18

**Authors:** Steven M. Offer, Qiang Pan-Hammarström, Lennart Hammarström, Reuben S. Harris

**Affiliations:** 1 Department of Biochemistry, Molecular Biology, and Biophysics, University of Minnesota, Minneapolis, Minnesota, United States of America; 2 Division of Clinical Immunology, Department of Laboratory Medicine, Karolinska Institutet at Karolinska University Hospital Huddinge, Stockholm, Sweden; New York University, United States of America

## Abstract

**Background:**

Despite considerable effort, the genetic factors responsible for >90% of the antibody deficiency syndromes IgAD and CVID remain elusive. To produce a functionally diverse antibody repertoire B lymphocytes undergo class switch recombination. This process is initiated by AID-catalyzed deamination of cytidine to uridine in switch region DNA. Subsequently, these residues are recognized by the uracil excision enzyme UNG2 or the mismatch repair proteins MutSα (MSH2/MSH6) and MutLα (PMS2/MLH1). Further processing by ubiquitous DNA repair factors is thought to introduce DNA breaks, ultimately leading to class switch recombination and expression of a different antibody isotype.

**Methodology/Principal Findings:**

Defects in *AID* and *UNG2* have been shown to result in the primary immunodeficiency hyper-IgM syndrome, leading us to hypothesize that additional, potentially more subtle, DNA repair gene variations may underlie the clinically related antibody deficiencies syndromes IgAD and CVID. In a survey of twenty-seven candidate DNA metabolism genes, markers in *MSH2*, *RAD50*, and *RAD52* were associated with IgAD/CVID, prompting further investigation into these pathways. Resequencing identified four rare, non-synonymous alleles associated with IgAD/CVID, two in *MLH1*, one in *RAD50*, and one in *NBS1*. One IgAD patient carried heterozygous non-synonymous mutations in *MLH1*, *MSH2*, and *NBS1*. Functional studies revealed that one of the identified mutations, a premature *RAD50* stop codon (*Q372X*), confers increased sensitivity to ionizing radiation.

**Conclusions:**

Our results are consistent with a class switch recombination model in which AID-catalyzed uridines are processed by multiple DNA repair pathways. Genetic defects in these DNA repair pathways may contribute to IgAD and CVID.

## Introduction

**Figure 1:**
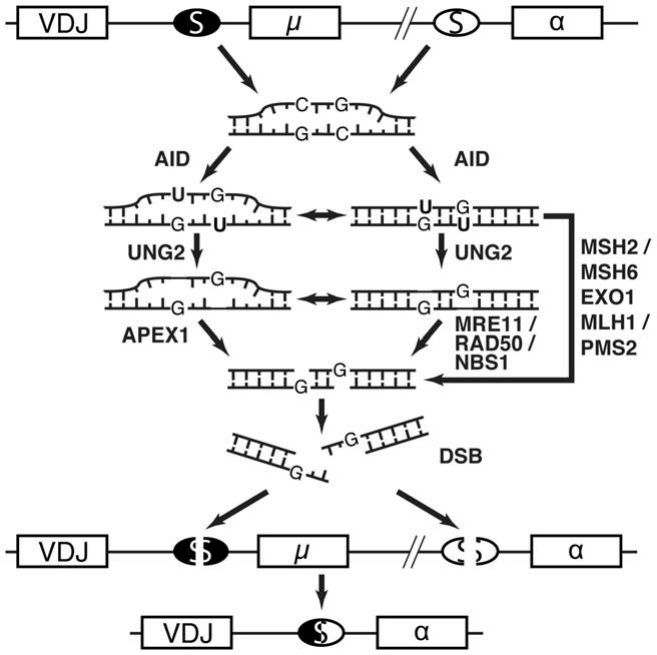
A model for DNA break generation by repair enzymes in class switch recombination. AID initiates CSR by deaminating switch (S) region DNA cytosines to uracils (the µ and α constant regions are depicted). These uracils are recognized and excised by the base excision repair enzyme UNG2 or the mismatch repair complex consisting of MutSa (MSH2/MSH6) and MutLa (PMS2/MLH1). The endonuclease activity of APEX1 or the MRN complex (MRE11-RAD50-NBS1) could then convert UNG2-generated abasic sites to single-strand breaks. The mismatch repair pathway (MSH2/MSH6, EXO1, MLH1/PMS2) could also introduce single-strand breaks at mismatched G:U bases. Opposing single-strand breaks may naturally lead to double-strand breaks, which can be further processed by non-homologous end joining factors to yield a complete CSR event. A switch from IgM to IgA is depicted, and the intervening switch circle is not shown.

Upon antigen stimulation, the constant region exons of the expressed antibody heavy chain gene can be replaced with downstream ones that encode an alternative antibody isotype (IgM -> IgG, IgE or IgA). At the molecular level, antibody class switch recombination (CSR) occurs between C/G-rich switch regions upstream of each set of constant region exons (Figure 1). In recent years a consensus model has emerged whereby CSR is initiated by activation-induced deaminase (AID)-catalyzed cytidine deamination to uridine within switch region DNA [1]–[5]. These DNA uridines are subsequently recognized and removed by the base excision repair enzyme uracil DNA glycosylase 2 (UNG2) or the mismatch repair proteins MutSα and MutLα (heterodimers of MSH2/MSH6 and MLH1/PMS2, respectively) [2], [6]–[13]. Additional base excision repair, mismatch repair, and recombination repair factors are then proposed to help convert these DNA repair intermediates to double-strand breaks and ultimately to CSR products [14]–[19].

A subset of primary antibody deficiency syndromes has been explained by defects in these DNA repair pathways. For instance, hyper-IgM syndrome type 2 (HIGM2) is caused by mutations in *AID* and this disease is characterized by high levels of IgM at the expense of the other antibody isotypes ([20]; reviewed in [21]). Mutations in *UNG2* lead to the less severe HIGM5 [22], and defects in *PMS2* have been associated with decreased antibody production [23]. Varying degrees of antibody deficiency have also been noted in chromosomal instability syndromes such as ataxia-telangiectasia (A-T, *ATM* mutations), Nijmegen breakage syndrome (NBS, *NBS1* mutations), and ataxia-telangiectasia-like disorder (ATLD, *MRE11* mutations) [24]–[30].

Prior studies have shown that missense mutations that impair MSH5 binding to its obligate heterodimerization partner MSH4 associate with immunoglobulin A deficiency (IgAD) and common variable immunodeficiency (CVID) [31]. IgAD and CVID often occur in different individuals of the same family, suggesting a common genetic components in at least a subset of patients [32]. Mutations in the B cell surface receptor genes *TACI* [33]–[35] and *CD19* [36], [37], and the T cell receptor gene *ICOS* [38] are also responsible for a subset of CVID cases. Nevertheless, despite this considerable progress, the genetic causes of >90% of IgAD and CVID cases have yet to be identified (reviewed in [39]–[41]).

Given that defects in DNA metabolism proteins are responsible for a fraction of primary antibody deficiencies, we hypothesized that variations in DNA repair genes could also underlie additional IgAD and CVID cases. To test this hypothesis, we performed a genetic association study of 27 candidate DNA metabolism genes in IgAD/CVID. Based on those results, we sequenced selected genes in a panel of 96 IgAD/CVID patients and subsequently genotyped the non-synonymous alleles we identified in a large case-control association study. Novel coding and non-coding variations were detected in several genes, including seven rare variants found only in IgAD and two specific to CVID. Four of these mutations, MLH1-*S247A*, MLH1-*Q409P*, NBS1-*P401R*, and RAD50-*Q372X* showed significant association with IgAD/CVID. One IgAD patient carried three previously unreported mutations, MSH2-*A727S*, MLH1-*G638R*, and NBS1-*P401R*. A second IgAD patient was compound heterozygous for RAD50-*P165H* and the previously reported RAD50-*R327H* variation. Two novel single nucleotide polymorphisms (SNPs) in the 3′ untranslated region of *MRE11* were also associated with CVID. Finally, both patient-derived and purposefully engineered cells harboring the RAD50-*Q372X* mutation exhibited increased sensitivity to ionizing radiation.

## Results

### IgAD/CVID association screen of 27 DNA repair genes

To screen for candidate genes in IgAD and CVID, we genotyped 140 IgAD patients, 48 CVID patients, and 92 healthy controls for SNPs selected from 26 known DNA repair genes and *AID* (collectively these genes define several DNA metabolism pathways). To test if a given SNP was associated with IgAD and/or CVID, allele frequencies were compared to the healthy control cohort. Significant single marker associations with IgAD and/or CVID (p<0.01) were noted for SNPs in the mismatch repair complexes, MutSα, MutSβ, and MutSγ, the MRN complex, the extended RAD52 epistasis group, and AID. A summary of single marker associations with p-values <0.01 is presented in Table 1. Full association data can be found in Table S1.

**Table 1:** SNPs in multiple DNA repair genes associate with IgAD/CVID.

		Controls	IgAD			CVID			Combined IgAD & CVID		
Gene	SNP	Allele Frequency	Allele Frequency	*p*-Value	Corrected *p*-Value a	Allele Frequency	*p*-Value	Corrected *p*-Value a	Allele Frequency	*p*-Value	Corrected *p*-Value a
*MSH2* b	rs4952887	14/184	31/280	0.22	0.96	18/96	0.0054	0.062	49/376	0.06	0.49
*MSH2* b	rs2347794	60/182	117/278	0.049	0.42	47/94	0.0059	0.070	164/372	0.012	0.17
*MSH2* b	rs3771274	62/184	118/278	0.059	0.46	49/96	0.0049	0.053	167/374	0.013	0.18
*MSH2* b	rs3771275	64/184	117/276	0.10	0.77	48/94	0.0088	0.098	165/370	0.027	0.30
*MSH2* b	rs3771276	59/182	116/276	0.038	0.34	48/96	0.0042	**0.0498**	164/372	0.0085	0.13
*MSH2* b	rs6729015	61/184	122/278	0.021	0.22	49/96	0.0036	**0.045**	171/374	0.0046	0.061
*MSH2* b	rs3771281	56/184	116/280	0.016	0.19	43/96	0.017	0.21	159/376	0.0068	0.079
*RAD50*	rs2237060	59/184	117/280	0.035	0.23	48/96	0.0034	**0.029**	165/376	0.0073	0.057
*RAD52*	rs9634161	28/184	54/280	0.26	1.00	27/96	0.0099	0.26	81/376	0.076	0.88
*RAD52*	rs10849605	75/178	123/276	0.61	1.00	59/94	0.0012	**0.040**	182/370	0.12	0.97
*RAD54B*	rs2046666	71/184	143/280	0.0083	0.23	46/96	0.13	0.98	189/376	0.0092	0.26
*RAD54B*	rs3019279	63/184	134/276	0.0024	0.076	47/94	0.011	0.29	181/370	0.0010	**0.035**
*RAD54B*	rs2921385	71/182	145/276	0.0045	0.14	48/96	0.078	0.89	193/372	0.0044	0.13
*RAD54B*	rs2930968	64/184	132/280	0.0084	0.23	47/94	0.014	0.35	179/374	0.0034	0.11
*AID*	rs2580874	73/184	151/280	0.0026	**0.010**	52/96	0.021	**0.021**	203/376	0.0015	**0.0055**
*AID*	rs1561559	2/184	15/280	0.017	0.068	2/94	0.49	0.49	17/374	0.034	0.13
*AID*	rs714629	73/178	142/274	0.024	0.086	49/90	0.037	**0.037**	191/364	0.012	**0.044**
*MSH5*	rs805304	68/182	170/280	9.2E-07	**0E-00** c	37/94	0.75	1.00	207/374	6.9E-05	**0.0020**
*MSH5*	rs3117572	142/182	251/278	2.7E-04	**0.0064**	76/96	0.83	1.00	327/374	0.0042	0.090
*MSH5*	rs3131379	27/182	87/278	6.4E-05	**0.0016**	14/92	0.93	1.00	101/370	0.0011	**0.024**
*MSH5*	rs3131378	26/182	89/280	2.1E-05	**5E-04**	18/96	0.33	1.00	107/376	2.3E-04	**0.0060**
*MSH5*	rs3117577	26/180	88/278	3.2E-05	**9E-04**	15/96	0.79	1.00	103/374	6.4E-04	**0.016**
*MSH5*	rs707938	63/180	154/276	1.4E-05	**3E-04**	35/96	0.81	1.00	189/372	4.7E-04	**0.012**

a ) Corrected for multiple testing bias by permutation testing as described in Materials and Methods.

b ) Corrected *p*-values derived using MutSα complex SNPs.

c ) No permutations exceeded those observed for SNP rs805304.

To further refine the list of candidates, we performed a relaxed correction for multiple testing by permutation testing markers grouped by DNA repair complex or pathway. After applying this statistical method, 13 SNPs remained significantly associated with IgAD/CVID (Table 1). Markers in *RAD54B* (rs3019279), *AID* (rs2580874 and rs714629) showed significant association with the combined IgAD/CVID cohort, and SNPs in MSH2 (rs3771276 and rs6729015), *RAD50* (rs2237060), and *RAD52* (rs10849605) were associated with IgAD. In agreement with our prior studies, six markers in the MHC class III region gene *MSH5* were associated with IgAD [31].

Next, we constructed multi-marker haplotypes and tested for association with IgAD and/or CVID. The markers comprising each haplotype block are listed in Table S2 and association data for haplotypes with a frequency greater than 0.1% are reported in Table S3. Significantly associated haplotypes with an uncorrected *p*-value less than 0.01 are summarized in Table 2. After pathway-based permutation testing, haplotypes of *MLH1*, *MSH5*, and *MUS81* were associated with IgAD and the combined IgAD/CVID group. Association with CVID was noted for haplotypes of *MSH2* and *RAD50*.

**Table 2:** Haplotypes blocks in *MLH1*, *MSH2*, *RAD50*, and *RAD54B* associate with IgAD/CVID.

	IgAD			CVID			Combined IgAD & CVID		
Haplotype	Hap Freq	*p*-Value	Corrected *p*-Value a	Hap Freq	*p*-Value	Corrected *p*-Value a	Hap Freq	*p*-Value	Corrected *p*-Value a
***MSH2*** – Block 16									
GCGACGCG	0.575	0.079	0.400	0.568	**0.0075**	**0.0250**	0.558	**0.013**	0.066
***MSH3*** – Block 18									
GTT	0.137	0.64	1.00	0.171	**0.0097**	0.14	0.157	0.19	1.00
***MSH3*** – Block 19									
GTC	0.009	**0.014**	0.20	0.014	0.14	0.95	0.007	**0.0043**	0.075
***MLH1*** – Block 11									
ATTATAT	0.008	**0.018**	**0.036**	0.013	0.16	0.64	0.006	**0.0062**	**0.014**
***RAD50*** – Block 23									
TGAGCA	0.365	0.064	0.500	0.367	**0.0062**	**0.041**	0.386	**0.011**	0.097
TGATCA	0.4	0.37	1.00	0.368	**0.0038**	**0.024**	0.373	0.061	0.63
***RAD54B*** – Block 26									
TAGGGCT	0.508	**0.0062**	0.15	0.55	0.086	0.96	0.503	**0.0057**	0.16
***MSH5*** – Block 21									
TACGATT	0.139	**2.0E-04**	**0.0033**	0.211	0.94	1.00	0.15	**0.0066**	0.095
TGCAGCC	0.243	**7.5E-05**	**0.0010**	0.146	0.99	1.00	0.227	**0.0017**	**0.023**
***MUS81*** – Block 22									
AATCA	0.011	**0.0053**	**0.014**	0.018	0.11	0.50	0.009	**0.0014**	**0.0054**

a ) Corrected for multiple testing bias by permutation testing as described in Materials and Methods.

### Identification of novel IgAD/CVID alleles

Since notable IgAD/CVID associations were observed for genes encoding DNA repair proteins that could convert AID-catalyzed uracils into DNA breaks, we resequenced select DNA repair genes in IgAD and CVID patient samples. Specifically, we sequenced the MutSα genes *MSH2* and *MSH6*, the MutLα gene *MLH1*, and the MRN complex genes *MRE11*, *RAD50*, and *NBS1*. The genes encoding AID, APEX1, ERCC1, and RAD52 were also sequenced.

In all, 242 genetic variants were detected, of which, 93 did not have records in dbSNP or SwissProt and were therefore considered novel (Table S4). 24 alleles encoded amino acid changes, 13 of which were previously unreported (all detected in a heterozygous state). Of the remaining 80 novel SNPs, 5 were synonymous coding alleles, 31 were located in mRNA untranslated regions (UTRs), 40 were intronic, and 4 were in flanking non-transcribed regions.

We next determined the allele frequencies of identified non-synonymous alleles and selected UTR, synonymous, and intronic SNPs in a large cohort of healthy controls, IgAD patients, and CVID patients. The most significant data are summarized in Table 3, and full datasets can be found in Table S5. In the mismatch repair pathway, two novel MSH2 variations were discovered in IgAD samples: MSH2-*T292S* and MSH2-*A727S*. The latter variation was unique to a single IgAD patient. Four novel amino acid mutations were detected in MLH1. MLH1-*S247A* and MLH1-*G638R* were specific to IgAD and MLH1-*Q409P* was specific to CVID (Table 3). MLH1-*H727L* and the previously reported mutations MLH1-*K618A*, MLH1-*V716M*, and MLH1-*I219V* occurred at similar frequencies in IgAD/CVID patients and controls.

**Table 3:** Genetic association of SNPs identified by reseqencing.

			Controls	IgAD		CVID	
Gene	SNP	Variant	Allele Frequency	Allele Frequency	*p*-Value	Allele Frequency	*p*-Value
*MSH2*	rs104895022	T292S	1/1890	2/652	0.10	0/230	0.73
*MSH2*	rs104895022	A727S	0/1888	**1/650** a	0.088	0/230	—
*MLH1*	rs1799977	I219V	563/1904	187/656	0.61	79/222	0.065
*MLH1*	rs104894996	S247A	0/1876	**2/642**	**0.016**	0/230	—
*MLH1*	rs104895000	Q409P	0/1902	0/656	—	**1/230**	**0.0040**
*MLH1*	rs35001569	K618A	13/1882	5/646	0.83	2/230	0.76
*MLH1*	rs63750549	G638R	0/1872	**1/642** a	0.088	0/230	—
*MLH1*	rs35831931	V716M	3/1904	1/658	0.98	1/230	0.36
*MLH1*	rs104895002	H727L	1/1872	0/642	0.56	1/228	0.075
*RAD50*	rs104895040	5′ UTR	0/1842	**1/640**	0.090	0/230	—
*RAD50*	rs104895041	5′ UTR	0/1900	**1/658**	0.089	0/230	—
*RAD50*	rs104895041	P165H	0/1842	**1/640**	0.0897	0/230	—
*RAD50*	rs28903091	R327H	4/1826	1/584	0.83	0/214	0.49
*RAD50*	rs104895046	Q372X	0/1900	0/654	—	**1/230**	**0.0040**
*RAD50*	rs104895051	R1077Q	0/1856	**1/596**	0.078	0/216	—
*RAD50*	rs104895053	SYN	0/1900	**1/652**	0.088	0/230	—
*MRE11*	rs104895004	3′ UTR	2/1866	2/636	0.26	3/228	**4.0E-04**
*MRE11*	rs104895010	3′ UTR	0/1864	0/636	—	**1/228**	**0.0042**
*MRE11*	rs104895016	A492D	2/1898	3/652	0.077	1/230	0.21
*MRE11*	rs61749249	E494K	8/1876	0/642	0.098	2/230	0.36
*NBS1*	rs104895039	Intron	0/1814	**1/602** a	0.083	0/226	—
*NBS1*	rs1805794	E185Q	625/1892	207/650	0.58	77/224	0.69
*NBS1*	rs61754796	V210F	4/1858	2/638	0.66	0/230	0.48
*NBS1*	rs104895033	P401R	0/1892	**2/646** a	**0.016**	0/230	—
*NBS1*	rs104895032	L421S	1/1884	0/644	0.56	1/230	0.076
*NBS1*	rs104895031	D527Y	0/1844	**1/598**	0.079	0/212	—
*RAD52*	rs7487683	G180R	74/1876	17/644	0.13	9/230	0.98
*APEX1*	rs2307490	5′ UTR	3/1872	2/642	0.46	2/230	**0.037**
*APEX1*	rs1048945	Q51H	60/1840	12/626	0.085	9/212	0.45

a ) A single IgAD patient carried all four alleles, MLH1-G638R, MSH2-A727S, NBS1-P401R, and the intronic SNP rs104895039.

The most striking variations were observed in *RAD50*. In all, 16 novel SNPs were found: 3 encoded amino acid changes, 2 were located in the 5′ UTR, 1 was synonymous, and 10 were intronic (Table S4). Most notably a single CVID sample contained the heterozygous transition mutation (1114C>T) that creates a premature stop codon at amino acid 372 (RAD50-*Q372X*, Table 3). One IgAD patient carried RAD50-*R1077Q* and another had both RAD50-*P165H* and RAD50-*R327H*. RAD50-*R327H* was also detected in our control samples, but never linked with RAD50-*P165H*. Pedigree analysis of the RAD50-*P165H/R327H* patient revealed that the two alleles were present in a compound heterozygous state.

Novel variations were also detected in the other two MRN complex genes, *MRE11* and *NBS1*. In *MRE11*, 18 novel SNPs were detected (Table S4). Of these, two resulted in amino acid changes (MRE11-*A492D* and MRE11-*E494K*), eight were in the 3′ UTR, and eight were intronic. Both MRE11-*A492D* and MRE11-*E494K* were found at similar frequencies in controls and IgAD/CVID cases (Table 3). Two 3′ UTR SNPs, ss206257789 and ss206257804, were, however, significantly enriched in the CVID population. In *NBS1*, 11 unreported SNPs were detected (Table S4). Three were non-synonymous, and the remaining 8 were a mixture of synonymous, 5′ UTR, 3′ UTR, and intronic. Two novel alleles, NBS1-*P401R* and NBS1-*D527Y*, were specific to IgAD (Table 3). NBS1-*V210F* and NBS1-*L421S* were detected in IgAD/CVID and control samples, as was the previously reported common polymorphism, rs1805794 (NBS1-*E185Q*).

A number of previously unreported SNPs were also discovered in other genes. In *AID*, two novel 3′ UTR SNPs and three novel synonymous SNPs were detected (Table S4). Two novel SNPs were found in *APEX1*, one synonymous and one in the 5′ UTR. The previously reported 5′ UTR SNP rs2307490 showed modest association with CVID (Table 3). In *ERCC1*, one 3′ flanking SNP and five novel intronic SNPs were detected (Table S4). 18 new SNPs were discovered in *RAD52*, 14 in the 3′ UTR, and four in introns. No amino acid substitution mutations variations were detected in these four genes.

While no patients were homozygous for any single SNP, some patients were found to carry mutant alleles in more than one DNA repair gene. An IgAD patient heterozygous for the previously reported APEX1-*Q51H* allele also carried three novel mutations, MLH1-*G638R*, MSH2-*A727S*, and NBS1-*P401R*. One IgAD patient carried both RAD50-*R1077Q* and MLH1-*K618A*. Additionally, as described earlier, an IgAD patient was found to be biallelic for RAD50-*P165H* and RAD50-*R327H*. These observations hint that the combinatorial effects of multiple mutations may underlie a subset of IgAD/CVID cases.

### RAD50-*Q372X* confers sensitivity to ionizing radiation

**Figure 2:**
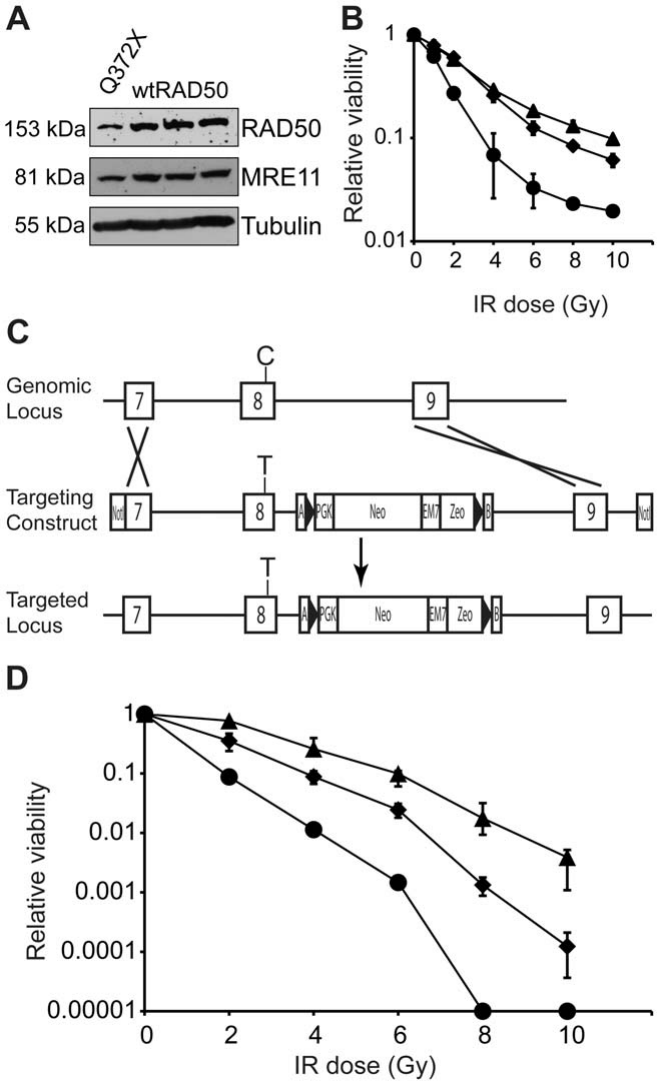
Heterozygous RAD50-Q372X cells show increased sensitivity to ionizing radiation. (**A**) Immunoblot of lysates from lymphoblast cell lines derived from the CVID patient carrying RAD50-*Q372X* and three CVID patients with no mutations in RAD50, MRE11, or NBS1 (wildtype, wtRAD50). (**B**) Viability of patient derived cell lines after exposure to ionizing radiation. Lymphoblast cell lines from the RAD50-*Q372X* patient (diamonds) and a CVID patient with no mutations in *RAD50*, *MRE11*, or *NBS1* (triangles) were exposed to the indicated doses of ionizing radiation and cell viability was assessed by Propidium Iodide staining and flow cytometry 10 days post-exposure. The Coriell cell line GM08436 (filled circles), containing heterozygous truncating mutations in ATM, was included as a positive control (Coriell Cell Repositories). Error bars denote the standard error of triplicate treatments. (**C**) The targeting strategy used to introduce the c.1114C>T mutation into the endogenous *RAD50* locus. Exons 7, 8, and 9 are depicted by boxes, and the position of c.1114C>T in exon 8 is marked. (**D**) Ionizing radiation sensitivity profiles of G418 resistant targeted clones with c.1114C>T (diamonds) or a wildtype *RAD50* exon 8 (triangles). It is notable that both of these clones have properly integrated drug resistance cassette, and they only differ by whether or not the flanking C-to-T mutation was co-recombined. The parental HCT116 cell line shows an ionizing sensitivity profile superimposable with that of the wildtype exon 8 clone (data not shown). DNA-PKcs-deficient cells [42] (circles) are extremely IR sensitive. Error bars represent the standard error of the mean sensitivity of four independent subclones (each assayed in duplicate).

Since CSR is likely to involve DNA breakage and end processing by exonucleases, we next decided to test the effect of one of the identified mutations (RAD50-*Q372X*) on cellular DNA repair capacity. To this end, immortalized B lymphoblast lines were generated from the CVID patient carrying RAD50-*Q372X*. Immunoblotting experiments indicated that RAD50 protein levels were reduced approximately 2-fold compared to three similarly derived cell lines from individuals with no detected RAD50 mutation (Figure 2a). MRE11 levels were also reduced approximately 2-fold, consistent with prior reports indicating that all three components of the MRN complex are subject to coordinated post-translational regulation [27]. Compared to a control EBV immortalized cell line, the *Q372X* cells were modestly, but significantly, sensitive to higher doses of ionizing radiation, but not to the extent of cells derived from an ataxia telangiectasia patient (Figure 2b).

To overcome the natural variability associated with patient-derived cell lines, we asked if we could recapitulate the sensitivity to ionizing radiation by introducing the RAD50 c.1114C>T mutation encoding for RAD50-*Q372X* into the endogenous locus of an immortalized human colon cancer cell line HCT116 (Figure 2c). HCT116 is a near-diploid line that has been used extensively in gene targeting experiments, particularly for studies of DNA metabolism proteins [42]. Targeting events were identified by insertion-specific PCR and confirmed by Southern blot using a flanking probe. Specific incorporation of the c.1114C>T mutation was confirmed by sequencing insertion-specific PCR products. Cells containing the heterozygous c.1114C>T mutation displayed an increased sensitivity to ionizing radiation compared to the control parental line, non-targeted sister clones, or targeted sister clones in which the mutation was not integrated (Figure 2d and data not shown). In comparison, a DNA-PKcs-deficient HCT116 cell line showed higher levels of ionizing radiation sensitivity [42]. These results demonstrate that the RAD50-*Q372X* mutation impairs the ability of the MRN complex to repair ionizing radiation-induced DNA double strand breaks.

## Discussion

We have shown that variations in multiple DNA repair pathways show association with the primary immunodeficiency syndromes CVID and IgAD. To our knowledge this is the first report associating immunodeficiency with markers in many of these genes, notably *MSH2*, *MLH1*, and *RAD50*. We also identified new non-synonymous alleles of *MSH2, MLH1, RAD50*, and *NBS1*, and UTR SNPs in *RAD50* and *MRE11* that were unique to IgAD and/or CVID. Cells carrying one of these mutations, RAD50-*Q372X*, were incapable of wildtype levels of recombination repair, likely due to haploinsufficiency. Overall, these data suggest that variations in the mismatch and recombination repair pathways may underlie some IgAD and CVID cases.

Mismatch repair proteins directly recognize and excise single base DNA mismatches and short patches of mismatched DNA (reviewed in [43]). Genetically, the involvement of mismatch repair in CSR has been well established in mice, with the most prominent defects observed in animals that also lack the uracil excision repair protein UNG2 [5]. Humans with heterozygous inactivating mutations in the mismatch repair proteins MSH2, MSH6, MLH1, or PMS2 are at increased risk for developing hereditary non-polyposis colorectal cancer (HNPCC) [44], [45]. IgA deficiency has been noted in a handful of HNPCC patients, one with a homozygous MSH2 mutation [46] and three with biallelic MSH6 mutations [47], [48].

**Figure 3:**
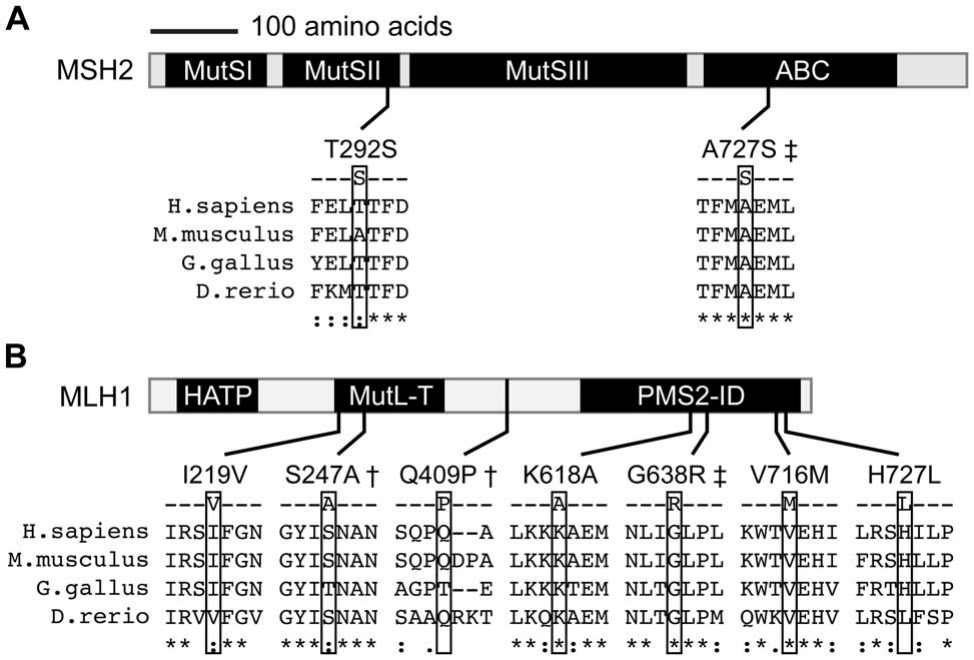
Location of the amino acid variations in the mismatch repair proteins MSH2 and MLH1. (**A**) MSH2 is divided into four domains. MutSI, II, and III represent a DNA binding globular region, an RNaseH-like motif, and a major structured domain, respectively, revealed by the *Thermus aquaticus* MutS crystal structure [59]. The fourth domain is an ABC-type ATPase domain [49]. (**B**) MLH1 has three domains, a histidine kinase-like ATPase domain (HATP), a MutL transducer domain (MutL-T), which is homologous to a domain in DNA gyrase B, and a PMS2 interaction domain (PMS2-ID). The indicated amino acid variations lie within conserved regions. Mutations associated with IgAD or CVID are denoted by †, those that were unique to the IgAD/CVID cohort but did not reach statistical significance are denoted by ‡. The illustrated protein segments were derived from ClustalW reference sequence alignments of the human, mouse, chicken and zebrafish proteins (MSH2, NP_000242, NP_032654, XP_426110, and NP_998689; MLH1, NP_000240, NP_081086, XP_418828, and NP_956953).

Our results are consistent with a role for mismatch repair in CSR. Most of the IgAD/CVID-specific alleles that we detected in the mismatch repair pathway are located in functionally important domains. MSH2-*A727S* maps to a conserved ABC domain (amino acids 718-731) that is predicted to be involved in ATP hydrolysis [49] (Figure 3). Two of the three IgAD/CVID specific mutations identified in *MLH1* map to domains of known importance. MLH1-*S247A* is located in a region that has both nucleotide hydrolysis and signal transduction functions [50]. Mutation of this residue to proline (*S247P*) has been reported in 14 HNPCC2 patients and is associated with decreased MLH1 protein expression and impaired mismatch repair activity using an *in-vitro* assay [51]. MLH1-*G638R* introduces a positively charged amino acid into a neutral area of the region known to be important for interaction with PMS2, MLH1, and PMS1 [52], [53]. Interestingly, two additional rare alleles that we detected in both IgAD/CVID cases and control samples were previously identified and characterized in HNPCC studies. MLH1-*K618A* was suggested to bind less efficiently to PMS2 [54], however follow-up studies with this allele found PMS2 binding and overall mismatch repair function to be normal [51]. The MLH1-*V716M* variant has been suggested to be a protective allele, as it was found enriched in controls compared to HNPCC cases [55]. Taken together, these results indicate that cancer predisposition alleles that are DNA damage sensitizing may also perturb the ability of the DNA repair pathways to elicit CSR.

**Figure 4:**
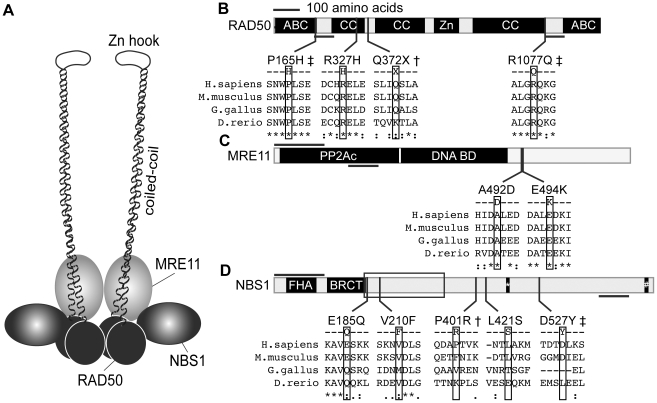
Location of the amino acid variations in the recombination proteins MRE11, RAD50, and NBS1. (**A**) A schematic of the MRN complex, indicating two copies of each protein and the extended coil-coil and zinc hook domains of RAD50. (**B, C, & D**) Schematics depicting the major protein domains of RAD50, MRE11, and NBS1, with the IgAD/CVID amino acid variations shown below aligned to the homologous regions of mouse, chicken, and zebrafish proteins. RAD50 has an N-terminal Walker A-type ATPase and DNA binding domain (ABC), three coiled-coil regions (CC), a zinc-hook (Zn), and a C-terminal Walker B-type ATPase and DNA binding domain (ABC). MRE11 has a protein phosphatase 2A-like catalytic domain (PP2AC) and a DNA binding domain (DNA BD). NBS1 has forkhead homology region (FHA) and a BRCA1 C-terminus-like domain (BRCT). A box in NBS1 indicates the location containing most of the mutations responsible for Nijmegen breakage syndrome. Mutations associated with IgAD or CVID are denoted by †, those that were unique to the IgAD/CVID cohort but did not reach statistical significance are denoted by ‡. The illustrated protein segments were derived from ClustalW reference sequence alignments (RAD50, NP_005723, NP_033038, XP_414645, and XP_696859; MRE11, NP_005582, NP_061206, NP_990109, and NP_001001407; NBS1, NP_002476, NP_038780, NP_989668, and NP_001014819).

Our results are the first to implicate MRN defects in IgAD/CVID. The premature stop codon encoded by the RAD50-*Q372X* allele likely leads to nonsense-mediated decay of the mRNA and haploinsufficiency (Figure 4). Other identified mutations may slightly perturb the structure and/or function of the MRN complex, and could act as weak hypomorphic alleles. The RAD50-*P165H* mutation replaces a neutral, non-polar proline with a positively charged, polar histidine at a position that is evolutionarily conserved through vertebrates. This residue is just outside of the N-terminal NTPase-containing globular domain and at the edge of the region that is thought to be important for interaction with MRE11. At the edge of the N-terminal MRE11-interating domain, RAD50-*R1077Q* replaces a conserved, positively charged arginine with a neutral glutamine. RAD50-*R327H*, which by itself did not associate with IgAD/CVID, exchanges one neutral, polar residue for another in the N-terminal coiled-coil domain. Neither of the additional IgAD/CVID-specific alleles detected in the MRN complex, NBS1-*P401R* and NBS1-*D527Y*, disrupt known or predicted functionally important protein domains. Future studies on the detected MRN alleles will provide greater insight into how they functionally impact recombination repair and CSR.

The association noted for two markers in *AID* suggests that a subclass of IgAD/CVID syndromes may be due to variations at this locus. These markers may be linked to undiscovered alleles that impact the expression of functional AID. This could be due to variations that create splice variants, UTR mutations that impact mRNA stability and protein expression, or mutations that impact transcription of *AID*, including alterations to transcription factor binding sites or sites important for epigenetic modulation of expression. In these studies, we identified two novel SNPs in the *AID* UTR and three novel synonymous mutations (Table S4). The further studies of these alleles may yield that some IgAD/CVID patients represent a new subclass of Hyper-IgM type 2 patients.

Overall, these studies suggest that, like many cancer predisposition syndromes such as HNPCC, IgAD and CVID may be a heterogeneous class of diseases due to defects in multiple pathways. Rather than straightforward causal alleles giving rise to disease, IgAD and CVID alleles likely represent risk factors that predispose an individual to developing immune deficiency. Additional studies to determine what effects these unique variations have on DNA repair and CSR are ongoing and long-term.

## Materials and Methods

### Ethics Statement

The Karolinska Institutet human subjects research review board provided written approval for the informed collection of DNA samples from CVID/IgAD patients for sequence analysis. The sample identities were unknown to the University of Minnesota team and therefore this arm of the project was exempt from institutional review.

### Human DNA samples

The initial association screen was performed in DNA from 140 IgAD patients, 48 CVID patients, and 92 healthy controls. To characterize all coding variation for the selected candidate genes in the CVID population, genomic resequencing was performed in 58 CVID DNAs, 48 from the original population and 10 that were subsequently recruited. 38 IgAD patients, each carrying at least one allele/haplotype associating with disease, were also included. 334 IgAD patients, 108 CVID patients, and 991 healthy controls were used for the subsequent large genetic association study of detected non-synonymous SNPs. Where adequate DNA was available, samples that had been used for earlier studies were utilized. All DNA samples used in this study were collected from unrelated individuals of self-reported Swedish descent and isolated at the Karolinska Institutet (Stockholm, Sweden). Informed consent was obtained from all subjects, and these studies were approved by the Karolinska Institutet human subjects research institutional review board.

### SNP selection and genotyping

For the initial association screen, tag SNPs were selected to cover the genomic locus of each gene plus an additional 10 kb upstream and 5 kb downstream using Tagger [56]. As phase II hapmap data was not yet available at the time, phase I data was used. Additional non-tag SNPs were included to fill out the assay. Genotypes were determined using the Illumina GoldenGate platform at the Broad Institute. Of the 384 markers genotyped, 81 failed to pass quality control tests and were not used in subsequent studies (Table S6). Two markers rs2020908 and rs689754 deviated significantly from Hardy-Weinberg equilibrium in the control DNA set (p<0.001), and 79 markers had genotype call rates that were below the 90% confidence threshold. Therefore, a total of 303 SNPs passed quality control criteria and were used in the subsequent studies.

For the large genetic association study, genotyping was performed by the BioMedical Genomics Center at the University of Minnesota. Two Sequenom iPLEX gold genotyping assays were designed using Spectrodesigner, part of the MassARRAY software package, to include non-synonymous coding SNPs detected by resequencing. Assays were filled out by including synonymous coding SNPs, UTR SNPs, and intronic SNPs located within 10 bases of an exon. Primers were synthesized by IDT and sequences are available upon request.

### DNA repair gene resequencing

Primer design, sequencing, and polymorphism detection were performed by the Broad Institute Center for Genotyping and Analysis. Briefly, primers were designed for each target gene to sequence the known exons, the 5′ UTR, and the 3′UTR, with at least 100 nucleotides of flanking sequence on each side. Genetic variants were identified by a combination of the PolyPhred and PolyDhan programs. Primer sequences for each assay are available upon request. To increase the power to detect informative alleles, the sample set was enriched for DNAs that carried disease-associated single markers and haplotypes as determined in the initial genotyping screen. In all, 3,086,784 nucleotides were examined, with average 1× coverage of 92.0% and 2× coverage of 88.4% (Tables S7). All novel non-synonymous SNPs were confirmed by fluorescence-based Sanger di-deoxy sequencing at either the University of Minnesota Sequencing and Analysis Facility or the Macrogen Company (South Korea).

### Data processing and statistical analyses

For both genotyping studies, allele calls were made using the appropriate built-in automatic allele calling specific to each platform. Call clustering was inspected manually and adjusted when obvious calling errors were made by the software. For genetic association studies, we required that each SNP have a missing genotype rate <10% and, in controls, have a Hardy-Weinberg p-value >0.001. Samples were excluded if genotyping failed for >50% of markers. Haploview was used to determine haplotype structure from control genotypes and to perform association testing for both single markers and haplotypes [57].

To correct for multiple testing bias, association results were subjected to 100,000 permutation tests by pathway using Haploview. For this analysis, genes were grouped based on functional pathways. Each group that contained at least one marker or haplotype with an uncorrected p-value <0.01 was permuted. These pathways were MutSα (*MSH2* and *MSH6*), MutSβ (*MSH2* and *MSH3*), MutLα (*MLH1* and *PMS2*), MRN (*MRE11*, *RAD50*, *NBS1*), MutSγ (*MSH4* and *MSH5*), and RAD51-mediated homologous recombination (*RAD51*, *RAD52*, *RAD54B*, *DMC1*, and *BLM*). *AID* and *MUS81* were permuted on an individual gene basis. Permutation testing was conducted for SNPs and haplotypes independently.

### RAD50-Q372X heterozygous cell lines

EBV-immortalized lymphoblast lines were generated at the Karolinska Institutet (Stockholm, Sweden). The HCT116-RAD50-c.1114C>T (*Q372X*) knock-in cell line and a wildtype knockin control line were generated using recombinant adeno-associated virus (rAAV)-assisted gene targeting, as previously described [42]. Briefly, targeting arms were amplified from genomic DNA by PCR and cloned into pJET1.2 (CloneJet PCR kit, Fermentas). Site directed mutagenesis was used to introduce the RAD50 c.1114C>T stop mutation and targeting arms were assembled into pNeDaKO [58]. rAAV was produced in HEK-293T cells and used to transduce HCT116 cells. The resulting G418-resistant clones were screened for targeted insertion by PCR and confirmed by Southern blotting. The introduction of the mutation was confirmed by sequencing.

### Immunoblots and ionizing radiation sensitivity experiments

Cell extracts were prepared using RIPA buffer, fractionated by SDS PAGE, and transferred to PVDF membrane for immunoblotting. The membranes were probed with antibodies to RAD50 (Novus), MRE11 (Genetex), or tubulin (Bethyl) followed by appropriate HRP-conjugated secondary antibodies (BioRad). Radiation sensitivity assays were conducted exposing cultures in a cesium irradiator for the indicated doses. Cell viability was assayed by Propidium Iodide staining and flow cytometry (lymphoblasts) or crystal violet colony staining of plated dilutions (HCT116 cells) [42].

## Supporting Information

Table S1 (0.12 MB PDF)

Table S2 (0.04 MB PDF)

Table S3 (0.09 MB PDF)

Table S4 (0.07 MB PDF)

Table S5 (0.05 MB PDF)

Table S6 (0.11 MB PDF)

Table S7 (0.04 MB PDF)
