# Supplementary material for: Unique DNA Repair Gene Variations and Potential Associations with the Primary Antibody Deficiency Syndromes IgAD and CVID
Source: PLoS One. 2010 Aug 18;5(8):e12260. doi: 10.1371/journal.pone.0012260 (PMC2923613; doi:10.1371/journal.pone.0012260)
Supplement: Table S1 — (0.12 MB PDF) [file pone.0012260.s001.pdf]

Table S1. Genetic association of single markers with IgAD and CVID. Bold values are used to highlight p-values <0.05 and allele frequencies of SNPs detected solely in patient groups. Statistics for the combined IgAD/CVID cohort are bold if p-values are more significant in the combined group compared to IgAD and CVID individually.

			Controls	IgAD			CVID			IgAD & CVID combined		
Gene	SNP	Associated Allele[a]	Allele frequency	Allele Frequency	p-value	Corrected p-value[b]	Allele Frequency	p-value	Corrected p-value[b]	Allele Frequency	p-value	Corrected p-value[b]
AID[c]	rs2028373	A	112/182	175/280	0.84	1.00	64/96	0.40	0.84	239/376	0.64	0.98
AID[c]	rs2580874	T	73/184	151/280	**0.0026**	**0.010**	52/96	**0.021**	0.073	203/376	**0.0015**	**0.0055**
AID[c]	rs1561559	G	2/184	15/280	**0.017**	0.068	2/94	0.49	0.93	17/374	**0.034**	**0.13**
AID_close[c]	rs714629	C	73/178	142/274	**0.024**	0.086	49/90	**0.037**	0.11	191/364	**0.012**	**0.044**
BLM[d]	rs7184015	G	138/184	207/278	0.90	1.00	76/96	0.44	1.00	283/374	0.86	1.00
BLM[d]	rs8034371	T	40/184	65/276	0.65	1.00	24/96	0.54	1.00	89/372	0.57	1.00
BLM[d]	rs6496724	G	50/182	86/280	0.46	1.00	29/94	0.56	1.00	115/374	0.43	1.00
BLM[d]	rs7183841	C	37/178	59/280	0.94	1.00	20/96	0.99	1.00	79/376	0.95	1.00
BLM[d]	rs6496725	C	38/182	67/276	0.40	1.00	21/96	0.85	1.00	88/372	0.46	1.00
BLM[d]	rs3784782	C	142/182	227/280	0.42	1.00	75/96	0.98	1.00	302/376	0.53	1.00
BLM[d]	rs2518968	C	99/176	161/274	0.60	1.00	59/96	0.41	1.00	220/370	0.48	1.00
BLM[d]	rs3784780	G	148/184	229/280	0.72	1.00	75/94	0.90	1.00	304/374	0.81	1.00
BLM[d]	rs3815003	G	49/184	83/278	0.45	1.00	28/94	0.58	1.00	111/372	0.43	1.00
BLM[d]	rs8036601	C	112/180	175/272	0.65	1.00	58/94	0.93	1.00	233/366	0.74	1.00
BLM[d]	rs4932363	A	9/184	20/280	0.33	1.00	7/94	0.39	1.00	27/374	0.29	1.00
BLM[d]	rs7175811	C	106/184	171/278	0.40	1.00	57/96	0.78	1.00	228/374	0.45	1.00
BLM[d]	rs2229035	-	0/182	0/274	--	--	0/96	--	--	0/370	--	--
BLM[d]	rs7182287	C	129/168	218/276	0.59	1.00	71/94	0.82	1.00	289/370	0.73	1.00
BLM[d]	rs1801256	T	0/180	**1/276**	0.42	1.00	0/96	--	1.00	1/372	0.49	1.00
BLM[d]	rs2270132	C	73/182	114/276	0.80	1.00	36/96	0.67	1.00	150/372	0.96	1.00
BLM[d]	rs2073919	G	138/182	222/280	0.38	1.00	72/96	0.88	1.00	294/376	0.53	1.00
BLM[d]	rs7167216	A	12/184	26/278	0.28	1.00	5/94	0.69	1.00	31/372	0.45	1.00
BLM[d]	rs414634	C	131/182	208/278	0.50	1.00	68/94	0.95	1.00	276/372	0.58	1.00
DMC1_close[d]	rs1946990	A	114/184	185/280	0.37	1.00	65/96	0.34	1.00	250/376	0.29	1.00
DMC1[d]	rs5750616	C	45/184	76/278	0.49	1.00	29/96	0.30	1.00	105/374	0.36	1.00
DMC1[d]	rs1980455	A	114/182	184/280	0.50	1.00	66/96	0.31	1.00	250/376	0.37	1.00
DMC1[d]	rs1292811	A	8/184	14/280	0.75	1.00	6/92	0.44	1.00	20/372	0.60	1.00
DMC1[d]	rs2227914	-	0/182	0/278	--	--	0/96	--	--	0/374	--	--
DMC1[d]	rs5757130	G	110/180	184/278	0.27	1.00	66/96	0.21	1.00	250/374	0.19	1.00
DMC1[d]	rs4821792	G	176/184	269/280	0.82	1.00	91/96	0.75	1.00	360/376	0.96	1.00
DMC1[d]	rs5757133	G	115/182	192/280	0.23	1.00	72/96	**0.046**	0.83	264/376	0.10	0.97
DMC1[d]	rs5757135	G	110/182	185/280	0.22	1.00	65/96	0.23	1.00	250/376	0.16	1.00
DMC1[d]	rs5757141	C	115/178	194/278	0.25	1.00	72/94	**0.042**	0.80	266/372	0.10	0.97
DMC1_close[d]	rs8140617	C	121/184	193/274	0.29	1.00	75/96	**0.032**	0.72	268/370	0.11	0.98
DMC1_close[d]	rs1569492	G	110/182	179/280	0.45	1.00	65/96	0.23	1.00	244/376	0.31	1.00
ERCC1_close	rs1046282	G	32/182	67/278	0.10	nd	23/94	0.17	nd	90/372	0.08	nd
ERCC1	rs3212977	-	0/184	0/276	--	--	0/94	--	--	0/370	--	--
ERCC1	rs11615	C	61/184	107/280	0.27	nd	35/92	0.42	nd	142/372	0.25	nd
ERCC1_close	rs1319052	T	52/178	110/280	**0.028**	nd	39/96	0.06	nd	149/376	**0.017**	nd
EXO1_close	rs10802995	T	72/184	122/280	0.34	nd	40/96	0.68	nd	162/376	0.37	nd
EXO1	rs1635518	T	86/184	131/274	0.82	nd	44/94	0.99	nd	175/368	0.86	nd
EXO1	rs4149864	-	0/182	0/276	--	--	0/90	--	--	0/366	--	--
EXO1	rs1635515	T	70/180	111/276	0.78	nd	50/94	**0.023**	nd	161/370	0.30	nd
EXO1	rs2526700	G	92/182	142/280	0.97	nd	52/94	0.45	nd	194/374	0.77	nd
EXO1	rs4149910	-	0/184	0/278	--	--	0/96	--	--	0/374	--	--
EXO1	rs1776133	C	73/180	109/272	0.92	nd	48/94	0.10	nd	157/366	0.60	nd
EXO1	rs2526698	A	92/184	142/278	0.82	nd	52/96	0.51	nd	194/374	0.68	nd
EXO1	rs735943	A	76/182	111/276	0.74	nd	49/92	0.07	nd	160/368	0.70	nd
EXO1	rs851781	C	92/184	148/280	0.55	nd	53/96	0.41	nd	201/376	0.44	nd
EXO1	rs4149963	A	16/182	31/278	0.41	nd	2/96	**0.031**	nd	33/374	0.99	nd
EXO1	rs4149966	G	1/184	3/280	0.55	nd	0/96	0.47	nd	3/376	0.74	nd
EXO1	rs4149967	-	0/184	0/280	--	--	0/96	--	--	0/376	--	--
EXO1	rs2526697	T	104/184	165/278	0.55	nd	47/96	0.23	nd	212/374	0.97	nd
EXO1	rs1047840	C	116/184	181/276	0.58	nd	70/94	0.06	nd	251/370	0.26	nd
EXO1	rs12122770	C	3/184	18/280	**0.015**	nd	3/94	0.40	nd	21/374	**0.029**	nd
EXO1	rs4149978	G	183/184	277/278	0.77	nd	94/94	0.47	nd	371/372	0.61	nd
EXO1	rs1776148	C	108/182	173/272	0.36	nd	61/96	0.50	nd	234/368	0.33	nd
EXO1	rs1635498	C	4/184	6/278	0.99	nd	4/96	0.34	nd	10/374	0.72	nd
EXO1	rs9350	A	24/184	50/280	0.17	nd	17/94	0.26	nd	67/374	0.14	nd
EXO1	rs4150018	G	66/184	127/280	**0.043**	nd	42/94	0.15	nd	169/374	**0.036**	nd
H2AFX	rs1784304	T	44/184	77/280	0.39	nd	23/96	0.99	nd	100/376	0.50	nd
H2AFX	rs7350	A	61/184	93/278	0.95	nd	37/96	0.37	nd	130/374	0.71	nd
H2AFX_close	rs643788	G	68/184	102/274	0.95	nd	40/94	0.37	nd	142/368	0.71	nd
H2AFX_close	rs649870	G	66/182	106/280	0.73	nd	44/96	0.12	nd	150/376	0.41	nd
LIG1	rs11668325	G	183/184	279/280	0.76	nd	96/96	0.47	nd	375/376	0.61	nd
LIG1	rs274883	G	24/184	44/280	0.43	nd	14/96	0.72	nd	58/376	0.45	nd
LIG1	rs156633	A	95/176	156/278	0.66	nd	49/94	0.77	nd	205/372	0.80	nd
LIG1	rs3731003	-	0/184	0/280	--	--	0/96	--	--	0/376	--	--
LIG1	rs156641	C	106/180	163/278	0.96	nd	60/96	0.56	nd	223/374	0.87	nd
LIG1	rs4987068	C	178/184	274/280	0.46	nd	95/96	0.26	nd	369/376	0.30	nd

Table S1. Genetic association of single markers with IgAD and CVID. Bold values are used to highlight p-values <0.05 and allele frequencies of SNPs detected solely in patient groups. Statistics for the combined IgAD/CVID cohort are bold if p-values are more significant in the combined group compared to IgAD and CVID individually.

			Controls	IgAD			CVID			IgAD & CVID combined		
Gene	SNP	Associated Allele[a]	Allele frequency	Allele Frequency	p-value	Corrected p-value [b]	Allele Frequency	p-value	Corrected p-value [b]	Allele Frequency	p-value	Corrected p-value [b]
LIG1	rs2304136	C	9/182	20/278	0.33	nd	6/96	0.65	nd	26/374	0.36	nd
LIG1	rs2288878	G	99/182	158/278	0.61	nd	49/94	0.72	nd	207/372	0.78	nd
LIG1	rs3730933	A	182/184	279/280	0.34	nd	95/96	0.97	nd	374/376	0.46	nd
LIG1	rs3730913	A	25/184	45/280	0.46	nd	15/96	0.64	nd	60/376	0.46	nd
LIG1	rs3730911	-	0/182	0/278	--	--	0/96	--	--	0/374	--	--
LIG1	rs11879148	-	0/184	0/280	--	--	0/96	--	--	0/376	--	--
LIG1	rs274860	A	9/184	16/278	0.69	nd	4/96	0.78	nd	20/374	0.82	nd
LIG1	rs12981963	A	0/184	**1/280**	0.42	nd	0/96	--	--	1/376	0.48	nd
LIG1	rs4987181	G	183/184	280/280	0.22	nd	96/96	0.47	nd	376/376	0.15	nd
LIG1_close	rs274869	C	85/182	131/280	0.99	nd	44/94	0.99	nd	175/374	0.98	nd
LIG1_close	rs274873	G	84/182	133/280	0.78	nd	47/94	0.54	nd	180/374	0.66	nd
MLH1_close	rs1055095	A	93/180	152/276	0.48	nd	57/94	0.16	nd	209/370	0.29	nd
MLH1	rs1800734	C	140/182	216/278	0.85	nd	85/96	**0.019**	nd	301/374	0.33	nd
MLH1	rs9852378	A	78/184	132/280	0.31	nd	47/96	0.29	nd	179/376	0.24	nd
MLH1	rs11541859	-	0/184	0/280	--	--	0/96	--	--	0/376	--	--
MLH1	rs4647224	T	79/180	132/280	0.49	nd	46/94	0.43	nd	178/374	0.41	nd
MLH1	rs4647250	C	75/178	130/278	0.33	nd	45/94	0.36	nd	175/372	0.28	nd
MLH1	rs4647257	-	0/184	0/280	--	--	0/96	--	--	0/376	--	--
MLH1	rs1558528	A	79/180	131/280	0.54	nd	48/96	0.33	nd	179/376	0.41	nd
MLH1	rs2286939	C	73/180	132/278	0.15	nd	47/96	0.18	nd	179/374	0.11	nd
MLH1	rs2286940	A	76/180	132/280	0.30	nd	48/96	0.22	nd	180/376	0.21	nd
MLH1	rs9311150	-	0/184	0/276	--	--	0/96	--	--	0/372	--	--
MLH1	rs2241031	T	83/184	134/280	0.56	nd	48/96	0.44	nd	182/376	0.46	nd
MLH1	rs1800149	-	0/184	0/280	--	--	0/96	--	--	0/376	--	--
MLH3	rs175047	G	83/178	121/274	0.61	nd	53/92	0.09	nd	174/366	0.84	nd
MLH3	rs108622	T	97/184	159/280	0.39	nd	43/96	0.21	nd	202/376	0.82	nd
MLH3	rs175052	-	0/184	0/280	--	--	0/96	--	--	0/376	--	--
MLH3	rs175057	A	88/184	125/280	0.50	nd	56/96	0.09	nd	181/376	0.94	nd
MLH3	rs2098252	G	95/184	154/278	0.43	nd	39/92	0.15	nd	193/370	0.91	nd
MLH3	rs175067	-	0/180	0/280	--	--	0/96	--	--	0/376	--	--
MLH3	rs735452	G	87/180	123/278	0.39	nd	58/96	0.06	nd	181/374	0.99	nd
MLH3	rs149220	A	0/184	**1/278**	0.42	nd	0/96	--	--	1/374	0.48	nd
MLH3	rs3742780	C	95/180	146/278	0.96	nd	59/94	0.11	nd	205/372	0.61	nd
MLH3	rs10136948	G	96/182	148/280	0.98	nd	60/96	0.12	nd	208/376	0.57	nd
MLH3	rs175081	-	0/184	0/280	--	--	0/96	--	--	0/376	--	--
MLH3	rs175082	-	0/184	0/280	--	--	0/96	--	--	0/376	--	--
MLH3_close	rs10142770	C	96/182	147/278	0.98	nd	58/94	0.16	nd	205/372	0.60	nd
MRE11 [e]	rs2155209	G	61/178	94/276	0.96	1.00	36/94	0.51	1.00	130/370	0.84	1.00
MRE11 [e]	rs1805362	-	0/182	0/280	--	--	0/96	--	--	0/376	--	--
MRE11 [e]	rs661957	T	59/180	91/270	0.84	1.00	36/92	0.30	0.97	127/362	0.59	1.00
MRE11 [e]	rs518276	G	114/184	181/280	0.56	1.00	66/94	0.17	0.90	247/374	0.34	0.99
MRE11 [e]	rs682213	T	98/180	164/278	0.34	0.99	62/96	0.10	0.56	226/374	0.18	0.89
MRE11 [e]	rs569143	C	104/182	174/280	0.28	0.98	65/96	0.09	0.48	239/376	0.14	0.66
MRE11 [e]	rs654718	A	121/184	192/280	0.53	1.00	69/96	0.30	0.97	261/376	0.38	0.99
MRE11 [e]	rs1805367	-	0/184	0/280	--	--	0/96	--	--	0/376	--	--
MRE11 [e]	rs1061945	-	0/184	0/278	--	--	0/96	--	--	0/374	--	--
MRE11 [e]	rs497763	G	97/184	165/280	0.19	0.80	62/96	0.06	0.35	227/376	0.08	0.49
MRE11_close [e]	rs472344	T	95/180	168/280	0.13	0.66	60/92	**0.050**	0.31	228/372	0.06	0.37
MSH2 [f,g]	rs12612908	A	0/184	**1/280**	0.42	0.42	0/96	--	--	1/376	0.48	1.00
MSH2 [f,g]	rs1863332	T	160/180	258/278	0.15	0.15	85/96	0.93	1.00	343/374	0.28	0.98
MSH2 [f,g]	rs4987188	T	1/182	7/278	0.11	0.11	0/94	0.47	1.00	7/372	0.22	0.97
MSH2 [f,g]	rs4952887	T	14/184	31/280	0.22	0.22	18/96	**0.0054**	0.062	49/376	0.06	0.49
MSH2 [f,g]	rs2347794	A	60/182	117/278	**0.049**	**0.049**	47/94	**0.0059**	0.070	164/372	**0.012**	0.17
MSH2 [f,g]	rs3771274	T	62/184	118/278	0.06	0.059	49/96	**0.0049**	0.053	167/374	**0.013**	0.18
MSH2 [f,g]	rs6726691	C	45/184	85/280	0.17	0.17	32/96	0.11	0.71	117/376	0.10	0.75
MSH2 [f,g]	rs1981928	T	47/182	81/280	0.47	0.47	29/94	0.38	1.00	110/374	0.38	1.00
MSH2 [f,g]	rs3771275	G	64/184	117/276	0.10	0.10	48/94	**0.0088**	0.098	165/370	**0.027**	0.30
MSH2 [f,g]	rs3771276	A	59/182	116/276	**0.038**	**0.038**	48/96	**0.0042**	**0.0498**	164/372	**0.0085**	0.13
MSH2 [f,g]	rs6729015	A	61/184	122/278	**0.021**	**0.021**	49/96	**0.0036**	**0.045**	171/374	**0.0046**	0.061
MSH2 [f,g]	rs3771281	A	56/184	116/280	**0.016**	**0.016**	43/96	**0.017**	0.21	159/376	**0.0068**	0.079
MSH2 [f,g]	rs3764959	G	47/178	79/274	0.57	0.57	30/96	0.39	1.00	109/370	0.46	1.00
MSH2 [f,g]	rs2042649	G	14/182	25/278	0.62	0.62	7/96	0.90	1.00	32/374	0.73	1.00
MSH2 [f,g]	rs1802577	-	0/184	0/280	--	--	0/96	--	--	0/376	--	--
MSH3_close [g]	rs844370	G	39/182	73/280	0.26	1.00	18/94	0.66	1.00	91/374	0.45	1.00
MSH3 [g]	rs380691	G	58/184	91/280	0.83	1.00	35/96	0.41	1.00	126/376	0.64	1.00
MSH3 [g]	rs1650670	C	40/178	75/276	0.26	1.00	19/96	0.61	1.00	94/372	0.47	1.00
MSH3 [g]	rs1650666	A	40/180	75/278	0.25	1.00	19/96	0.64	1.00	94/374	0.45	1.00
MSH3 [g]	rs1650663	C	42/180	74/276	0.40	1.00	17/94	0.32	1.00	91/370	0.75	1.00
MSH3 [g]	rs1677649	T	44/180	74/276	0.57	1.00	19/94	0.43	1.00	93/370	0.86	1.00
MSH3 [g]	rs6864493	C	39/184	72/280	0.26	0.72	34/96	**0.010**	0.17	106/376	0.08	0.72
MSH3 [g]	rs3776968	T	58/184	108/278	0.11	0.71	39/96	0.13	0.89	147/374	0.07	0.71

Table S1. Genetic association of single markers with IgAD and CVID. Bold values are used to highlight p-values <0.05 and allele frequencies of SNPs detected solely in patient groups. Statistics for the combined IgAD/CVID cohort are bold if p-values are more significant in the combined group compared to IgAD and CVID individually.

			Controls	IgAD			CVID			IgAD & CVID combined		
Gene	SNP	Associated Allele[a]	Allele frequency	Allele Frequency	p-value	Corrected p-value[b]	Allele Frequency	p-value	Corrected p-value[b]	Allele Frequency	p-value	Corrected p-value[b]
MSH3[g]	rs26282	G	136/184	203/278	0.83	1.00	77/96	0.24	0.99	280/374	0.81	1.00
MSH3[g]	rs33013	A	53/182	88/280	0.60	1.00	35/96	0.21	0.98	123/376	0.39	1.00
MSH3[g]	rs6151838	T	165/184	254/280	0.71	1.00	84/94	0.94	1.00	338/374	0.79	1.00
MSH3[g]	rs42290	T	26/184	46/280	0.50	1.00	10/96	0.38	1.00	56/376	0.81	1.00
MSH3[g]	rs3797896	G	164/180	263/280	0.25	0.83	93/96	0.07	0.68	356/376	0.11	0.83
MSH3[g]	rs245391	G	43/182	80/280	0.24	0.58	38/94	**0.0037**	0.070	118/374	0.05	0.58
MSH3[g]	rs32991	T	99/184	152/276	0.79	1.00	63/96	0.06	0.62	215/372	0.37	1.00
MSH3[g]	rs26910	T	22/178	38/270	0.60	0.96	23/94	**0.011**	0.18	61/364	0.18	0.96
MSH3[g]	rs3776978	A	21/184	38/280	0.49	1.00	13/96	0.60	1.00	51/376	0.48	1.00
MSH3[g]	rs184967	G	155/184	235/280	0.93	1.00	86/96	0.22	0.99	321/376	0.72	1.00
MSH3[g]	rs245341	A	42/178	79/278	0.26	1.00	20/96	0.60	1.00	99/374	0.47	1.00
MSH3[g]	rs27887	T	46/182	80/280	0.44	1.00	20/94	0.46	1.00	100/374	0.71	1.00
MSH3_close[g]	rs32964	C	47/184	79/280	0.53	1.00	20/96	0.38	1.00	99/376	0.84	1.00
MSH4_close[h]	rs1498311	C	132/184	204/280	0.79	1.00	72/96	0.56	1.00	276/376	0.68	1.00
MSH4[h]	rs1146646	T	40/178	72/280	0.43	1.00	29/94	0.13	0.92	101/374	0.25	0.99
MSH4[h]	rs5745325	C	128/182	203/280	0.61	1.00	71/96	0.52	1.00	274/376	0.53	1.00
MSH4[h]	rs5745327	A	163/182	255/278	0.43	1.00	87/96	0.78	1.00	342/374	0.47	1.00
MSH4[h]	rs5745329	T	0/180	**1/278**	0.42	1.00	**1/96**	0.17	0.97	**2/374**	0.33	1.00
MSH4[h]	rs1146652	G	170/184	262/280	0.62	1.00	91/96	0.45	1.00	353/376	0.50	1.00
MSH4[h]	rs1144342	C	163/182	257/280	0.42	1.00	87/96	0.78	1.00	344/376	0.46	1.00
MSH4[h]	rs5745390	G	38/182	67/280	0.44	1.00	26/96	0.24	1.00	93/376	0.31	1.00
MSH4[h]	rs1565717	A	38/184	64/278	0.55	1.00	24/94	0.36	1.00	88/372	0.43	1.00
MSH4[h]	rs5745458	A	142/182	221/278	0.70	1.00	74/96	0.86	1.00	295/374	0.82	1.00
MSH4[h]	rs5745459	A	177/184	274/280	0.29	1.00	93/94	0.20	0.99	367/374	0.17	0.97
MSH4[h]	rs1001160	A	131/182	201/278	0.94	1.00	69/94	0.80	1.00	270/372	0.88	1.00
MSH4[h]	rs5745543	A	127/178	196/274	0.97	1.00	67/92	0.80	1.00	263/366	0.90	1.00
MSH4[h]	rs5745545	A	131/184	201/280	0.89	1.00	70/96	0.76	1.00	271/376	0.83	1.00
MSH4[h]	rs5745549	A	6/180	14/280	0.39	1.00	5/94	0.43	1.00	19/374	0.35	1.00
MSH4_close[h]	rs946163	G	132/184	203/280	0.86	1.00	70/94	0.63	1.00	273/374	0.75	1.00
MSH5_close[h]	rs805304	G	68/182	170/280	**9.2E-07**	**0E-00**[g]	37/94	0.75	1.00	207/374	**6.9E-05**	**0.00**
MSH5_close[h]	rs400547	A	10/184	24/278	0.20	0.97	6/96	0.78	1.00	30/374	0.27	1.00
MSH5[h]	rs3131382	G	172/184	271/280	0.09	0.81	90/96	0.93	1.00	361/376	0.19	0.98
MSH5[h]	rs409558	G	30/184	56/280	0.32	1.00	17/96	0.77	1.00	73/376	0.37	1.00
MSH5[h]	rs28381349	T	4/184	9/278	0.50	1.00	3/96	0.63	1.00	12/374	0.49	1.00
MSH5[h]	rs707915	T	10/184	24/280	0.20	0.97	5/96	0.94	1.00	29/376	0.32	1.00
MSH5[h]	rs2075788	G	30/184	56/280	0.32	1.00	17/96	0.77	1.00	73/376	0.37	1.00
MSH5[h]	rs3117572	G	142/182	251/278	**2.7E-04**	**0.01**	76/96	0.83	1.00	327/374	**0.0042**	**0.09**
MSH5[h]	rs2299851	T	20/184	31/280	0.95	1.00	10/96	0.91	1.00	41/376	0.99	1.00
MSH5[h]	rs3131379	A	27/182	87/278	**6.4E-05**	**0.00**	14/92	0.93	1.00	101/370	**0.0011**	**0.02**
MSH5[h]	rs3131378	G	26/182	89/280	**2.1E-05**	**0.00**	18/96	0.33	1.00	107/376	**2.3E-04**	**0.01**
MSH5[h]	rs707939	C	129/180	226/280	**0.024**	**0.38**	66/96	0.61	1.00	292/376	0.12	0.90
MSH5[h]	rs3117577	C	26/180	88/278	**3.2E-05**	**0.00**	15/96	0.79	1.00	103/374	**6.4E-04**	**0.02**
MSH5[h]	rs707938	C	63/180	154/276	**1.4E-05**	**0.00**	35/96	0.81	1.00	189/372	**4.7E-04**	**0.01**
MSH5[h]	rs1802127	C	180/184	267/274	0.79	1.00	95/96	0.50	1.00	362/370	0.99	1.00
MSH5_close[h]	rs707937	G	147/182	232/278	0.46	1.00	83/96	0.23	1.00	315/374	0.31	1.00
MSH5_close[h]	rs707936	T	12/184	25/280	0.35	1.00	7/96	0.81	1.00	32/376	0.41	1.00
MSH6[f]	rs3136245	T	39/184	71/280	0.30	0.98	18/94	0.69	1.00	89/374	0.49	1.00
MSH6[f]	rs1878484	A	182/184	277/280	0.99	1.00	93/94	0.99	1.00	370/374	0.99	1.00
MSH6[f]	rs1800932	C	26/180	45/280	0.64	1.00	18/96	0.35	1.00	63/376	0.49	1.00
MSH6[f]	rs3211299	-	0/178	0/274	--	--	0/96	--	--	0/370	--	--
MSH6[f]	rs1800938	-	0/184	0/280	--	--	0/96	--	--	0/376	--	--
MSH6[f]	rs3136334	-	0/184	0/280	--	--	0/96	--	--	0/376	--	--
MSH6[f]	rs2020912	A	182/184	275/278	0.99	1.00	96/96	0.31	0.99	371/374	0.74	1.00
MSH6[f]	rs2020911	T	63/184	114/278	0.14	0.85	37/96	0.48	1.00	151/374	0.16	0.91
MUS81_close	rs635375	G	47/178	91/280	0.17	nd	28/94	0.55	nd	119/374	0.19	nd
MUS81_close	rs652021	G	49/182	91/280	0.20	nd	31/96	0.35	nd	122/376	0.18	nd
MUS81_close	rs665306	T	58/174	99/268	0.44	nd	33/96	0.86	nd	132/364	0.51	nd
MUS81	rs630303	C	54/184	91/280	0.47	nd	28/94	0.94	nd	119/374	0.55	nd
MUS81	rs765593	-	0/174	0/274	--	--	0/96	--	--	0/370	--	--
MUS81_close	rs659824	A	81/178	135/274	0.43	nd	43/90	0.72	nd	178/364	0.46	nd
MUS81_close	rs630394	C	57/182	93/276	0.60	nd	28/94	0.79	nd	121/370	0.74	nd
NBS1[e]	rs3026268	-	0/182	0/280	--	--	0/96	--	--	0/376	--	--
NBS1[e]	rs769420	-	0/184	0/280	--	--	0/92	--	--	0/372	--	--
NBS1[e]	rs1805794	C	112/178	183/280	0.60	1.00	61/92	0.58	1.00	244/372	0.54	1.00
PMS2_close	rs710939	C	3/184	8/280	0.40	nd	2/96	0.79	nd	10/376	0.45	nd
PMS2_close	rs852520	A	95/184	168/278	0.06	nd	60/96	0.08	nd	228/374	**0.036**	nd
PMS2_close	rs852516	T	11/184	25/280	0.25	nd	11/96	0.11	nd	36/376	0.15	nd
PMS2_close	rs852417	G	118/184	192/278	0.27	nd	62/96	0.94	nd	254/374	0.37	nd
PMS2_close	rs852413	A	3/184	9/280	0.29	nd	2/96	0.79	nd	11/376	0.36	nd
PMS2_close	rs3779092	G	87/178	151/278	0.26	nd	46/94	0.99	nd	197/372	0.37	nd

Table S1. Genetic association of single markers with IgAD and CVID. Bold values are used to highlight p-values <0.05 and allele frequencies of SNPs detected solely in patient groups. Statistics for the combined IgAD/CVID cohort are bold if p-values are more significant in the combined group compared to IgAD and CVID individually.

			Controls	IgAD			CVID			IgAD & CVID combined		
Gene	SNP	Associated Allele[a]	Allele frequency	Allele Frequency	p-value	Corrected p-value[b]	Allele Frequency	p-value	Corrected p-value[b]	Allele Frequency	p-value	Corrected p-value[b]
PMS2_close	rs852394	T	79/176	127/276	0.81	nd	43/94	0.89	nd	170/370	0.82	nd
PMS2_close	rs4724712	A	84/184	128/280	0.99	nd	46/94	0.60	nd	174/374	0.85	nd
PMS2_close	rs852266	C	10/182	25/280	0.17	nd	10/96	0.13	nd	35/376	0.12	nd
PMS2_close	rs852262	G	2/184	8/278	0.20	nd	3/96	0.22	nd	11/374	0.17	nd
PMS2_close	rs1468996	T	67/180	113/276	0.43	nd	37/94	0.73	nd	150/370	0.45	nd
PMS2	rs1805321	T	0/184	**5/280**	0.068	nd	**1/96**	0.17	nd	**6/376**	0.085	nd
PMS2_close	rs2009115	A	14/182	42/278	**0.017**	nd	12/96	0.19	nd	54/374	**0.023**	nd
PMS2_close	rs3779107	G	14/182	44/280	**0.011**	nd	12/96	0.19	nd	56/376	**0.016**	nd
PMS2_close	rs1860459	A	18/184	25/280	0.76	nd	13/96	0.34	nd	38/376	0.90	nd
POLH_close	rs699937	T	52/182	86/274	0.52	nd	25/94	0.73	nd	111/368	0.70	nd
POLH	rs6458343	A	10/182	12/278	0.56	nd	9/96	0.22	nd	21/374	0.95	nd
POLH	rs2307456	G	183/184	280/280	0.22	nd	96/96	0.47	nd	376/376	0.15	nd
POLH	rs9333548	-	0/184	0/278	--	--	0/94	--	--	0/372	--	--
RAD50_close[e]	rs2522410	A	0/184	**2/280**	0.25	0.98	**1/96**	0.17	0.88	**3/376**	0.22	0.96
RAD50[e]	rs4526098	C	0/184	**1/280**	0.42	1.00	0/96	--	--	1/376	0.48	1.00
RAD50[e]	rs2244012	T	138/184	222/280	0.28	0.98	74/96	0.70	1.00	296/376	0.32	0.99
RAD50[e]	rs2706348	G	137/182	217/272	0.26	0.98	74/96	0.74	1.00	291/368	0.31	0.98
RAD50[e]	rs2230017	-	0/184	0/280	--	--	0/96	--	--	0/376	--	--
RAD50[e]	rs2252775	A	137/182	219/278	0.38	0.99	74/96	0.74	1.00	293/374	0.42	1.00
RAD50[e]	rs1047380	-	0/184	0/276	--	--	0/94	--	--	0/370	--	--
RAD50[e]	rs1047382	-	0/184	0/280	--	--	0/96	--	--	0/376	--	--
RAD50[e]	rs3187395	G	183/184	278/278	0.22	0.93	96/96	0.47	1.00	374/374	0.15	0.86
RAD50[e]	rs1047386	-	0/184	0/280	--	--	0/96	--	--	0/376	--	--
RAD50[e]	rs1047387	-	0/184	0/278	--	--	0/96	--	--	0/374	--	--
RAD50[e]	rs2237060	G	59/184	117/280	**0.035**	0.23	48/96	**0.0034**	**0.029**	165/376	**0.0073**	0.057
RAD50[e]	rs2240032	C	138/184	221/278	0.26	0.98	72/94	0.77	1.00	293/372	0.32	0.99
RAD50_close[e]	rs2158177	A	141/184	227/280	0.25	0.95	73/94	0.85	1.00	300/374	0.33	0.99
RAD51_close[d]	rs2412545	G	109/184	185/280	0.14	0.99	56/96	0.88	1.00	241/376	0.26	1.00
RAD51[d]	rs2619681	T	25/184	47/280	0.35	1.00	13/96	0.99	1.00	60/376	0.46	1.00
RAD51[d]	rs7174493	-	0/184	0/280	--	--	0/96	--	--	0/376	--	--
RAD51[d]	rs2412546	G	83/182	133/278	0.64	1.00	39/96	0.43	1.00	172/374	0.93	1.00
RAD51[d]	rs11858337	G	85/182	137/276	0.54	1.00	40/94	0.51	1.00	177/370	0.80	1.00
RAD51[d]	rs957603	T	72/182	119/276	0.45	1.00	35/94	0.71	1.00	154/370	0.64	1.00
RAD51[d]	rs11070291	A	83/184	138/280	0.38	1.00	41/96	0.70	1.00	179/376	0.58	1.00
RAD51[d]	rs1056742	-	0/184	0/278	--	--	0/96	--	--	0/374	--	--
RAD51_close[d]	rs4924501	G	84/184	140/278	0.32	1.00	41/94	0.75	1.00	181/372	0.50	1.00
RAD51_close[d]	rs11558809	C	179/180	280/280	0.21	1.00	96/96	0.46	1.00	376/376	0.15	1.00
RAD52[d]	rs1060499	G	29/182	48/276	0.68	1.00	25/94	**0.034**	0.74	73/370	0.28	1.00
RAD52[d]	rs1051669	G	144/180	223/280	0.93	1.00	82/96	0.27	1.00	305/376	0.75	1.00
RAD52[d]	rs10744729	G	83/182	138/276	0.36	1.00	54/94	0.06	0.91	192/370	0.16	1.00
RAD52[d]	rs4766370	A	82/182	137/280	0.42	1.00	54/96	0.08	0.94	191/376	0.20	1.00
RAD52[d]	rs9634161	C	28/184	54/280	0.26	1.00	27/96	**0.010**	0.34	81/376	0.08	0.93
RAD52[d]	rs7312883	-	0/184	0/280	--	--	0/96	--	--	0/376	--	--
RAD52[d]	rs7487683	T	3/184	13/280	0.08	0.95	6/96	**0.038**	0.77	19/376	0.05	0.85
RAD52[d]	rs4766377	A	144/182	223/280	0.89	1.00	82/96	0.20	1.00	305/376	0.58	1.00
RAD52[d]	rs1131839	-	0/184	0/280	--	--	0/96	--	--	0/376	--	--
RAD52[d]	rs1833095	C	48/184	75/280	0.87	1.00	25/96	0.99	1.00	100/376	0.90	1.00
RAD52[d]	rs7311151	G	77/184	116/280	0.93	1.00	43/96	0.64	1.00	159/376	0.92	1.00
RAD52[d]	rs2887531	A	134/184	204/280	0.99	1.00	72/96	0.70	1.00	276/376	0.88	1.00
RAD52_close[d]	rs10849605	A	75/178	123/276	0.61	1.00	59/94	**0.0012**	0.060	182/370	0.12	0.99
RAD54B[d]	rs2046666	C	71/184	143/280	**0.0083**	0.27	46/96	0.13	0.99	189/376	**0.0092**	0.32
RAD54B[d]	rs2470740	T	72/180	144/276	**0.0109**	0.36	46/96	0.21	1.00	190/372	**0.015**	0.44
RAD54B[d]	rs2046663	A	16/184	32/278	0.33	1.00	11/96	0.46	1.00	43/374	0.31	1.00
RAD54B[d]	rs3019149	A	47/184	97/280	**0.038**	0.76	34/96	0.08	0.96	131/376	**0.026**	0.64
RAD54B[d]	rs3136421	T	10/184	21/280	0.38	1.00	7/96	0.54	1.00	28/376	0.37	1.00
RAD54B[d]	rs3019279	G	63/184	134/276	**0.0024**	0.094	47/94	**0.011**	0.36	181/370	**0.0010**	**0.046**
RAD54B[d]	rs2921385	C	71/182	145/276	**0.0045**	0.17	48/96	0.08	0.95	193/372	**0.0044**	0.16
RAD54B[d]	rs2930968	A	64/184	132/280	**0.0084**	0.28	47/94	**0.014**	0.44	179/374	**0.0034**	0.14
RAD54B[d]	rs1372048	G	49/184	95/278	0.09	0.96	35/96	0.09	0.96	130/374	0.05	0.86
RAD54B[d]	rs2919661	-	0/184	0/278	--	--	0/92	--	1.00	0/370	--	--
RAD54B_close[d]	rs1992371	T	66/178	131/280	**0.041**	0.78	42/96	0.28	1.00	173/376	**0.047**	0.82
SPO11	rs3736832	-	0/182	0/280	--	--	0/94	--	--	0/374	--	--
SPO11	rs6099553	G	112/184	175/280	0.72	nd	67/94	0.09	nd	242/374	0.38	nd
SPO11	rs1467581	T	2/180	8/274	0.20	nd	6/96	**0.015**	nd	14/370	0.08	nd
TP53BP1	rs1058298	T	53/180	86/280	0.77	nd	28/94	0.95	nd	114/374	0.80	nd
TP53BP1	rs11554564	-	0/184	0/280	--	--	0/96	--	--	0/376	--	--
TP53BP1	rs2230449	-	0/184	0/280	--	--	0/96	--	--	0/376	--	--
TP53BP1	rs2242069	C	29/184	55/280	0.29	nd	17/96	0.68	nd	72/376	0.33	nd
TP53BP1	rs542898	A	56/184	88/280	0.82	nd	28/96	0.83	nd	116/376	0.92	nd

Table S1. Genetic association of single markers with IgAD and CVID. Bold values are used to highlight p-values <0.05 and allele frequencies of SNPs detected solely in patient groups. Statistics for the combined IgAD/CVID cohort are bold if p-values are more significant in the combined group compared to IgAD and CVID individually.

			Controls	IgAD			CVID			IgAD & CVID combined		
Gene	SNP	Associated Allele[a]	Allele frequency	Allele Frequency	p-value	Corrected p-value [b]	Allele Frequency	p-value	Corrected p-value [b]	Allele Frequency	p-value	Corrected p-value [b]
TP53BP1	rs2602141	T	128/184	194/280	0.95	nd	68/96	0.83	nd	262/376	0.98	nd
TP53BP1	rs536313	T	50/176	85/280	0.66	nd	28/96	0.89	nd	113/376	0.69	nd
TP53BP1	rs2256238	-	0/182	0/276	--	--	0/96	--	--	0/372	--	--
TP53BP1	rs2467739	A	13/180	37/280	**0.044**	nd	10/96	0.36	nd	47/376	0.06	nd
TP53BP1	rs2467741	C	56/182	93/280	0.58	nd	29/96	0.92	nd	122/376	0.69	nd
TP53BP1	rs694725	C	54/182	84/278	0.90	nd	27/96	0.79	nd	111/374	1.00	nd
TP53BP1	rs689647	T	15/184	34/280	0.17	nd	10/96	0.53	nd	44/376	0.20	nd
TP53BP1	rs560191	G	58/184	92/280	0.76	nd	30/94	0.95	nd	122/374	0.79	nd
TP53BP1	rs2439850	G	15/182	34/278	0.18	nd	8/94	0.94	nd	42/372	0.27	nd
TP53BP1_close	rs1869258	G	54/184	87/280	0.69	nd	28/96	0.97	nd	115/376	0.76	nd
TP53BP1_close	rs523156	G	131/184	198/278	0.99	nd	70/94	0.56	nd	268/372	0.83	nd
XRCC2_close	rs6962238	G	8/184	25/280	0.06	nd	8/96	0.17	nd	33/376	0.06	nd
XRCC2	rs3218536	T	10/180	18/280	0.70	nd	6/96	0.81	nd	24/376	0.70	nd
XRCC2	rs3111471	T	88/184	149/280	0.26	nd	52/96	0.31	nd	201/376	0.21	nd
XRCC2	rs3218408	G	44/184	72/278	0.63	nd	24/96	0.84	nd	96/374	0.65	nd
XRCC2_close	rs2040639	C	85/182	149/280	0.17	nd	50/94	0.31	nd	199/374	0.15	nd
XRCC2_close	rs6464268	T	162/184	250/280	0.68	nd	82/96	0.53	nd	332/376	0.93	nd
XRCC2_close	rs10227264	G	34/182	55/280	0.80	nd	28/94	**0.036**	nd	83/374	0.34	nd
XRCC2_close	rs684088	A	109/184	162/280	0.77	nd	61/96	0.48	nd	223/376	0.99	nd
XRCC2_close	rs513586	C	171/184	266/278	0.20	nd	87/96	0.50	nd	353/374	0.50	nd
XRCC2_close	rs2018083	T	121/182	189/276	0.66	nd	64/88	0.30	nd	253/364	0.47	nd
XRCC3_close	rs2273175	G	67/184	109/278	0.54	nd	31/94	0.57	nd	140/372	0.78	nd
XRCC3_close	rs941474	A	87/176	136/274	0.97	nd	49/96	0.80	nd	185/370	0.90	nd

a) as determined in the combined IgAD/CVID cohort
b) p-value corrected for multiple testing bias by 100,000 permutations of all markers within pathways as described in Materials and Methods
c) Permutation tests performed on markers in AID
d) Permutation tests performed on markers of the RAD52 extended epistasis group
e) Permutation tests performed on markers of the MRN complex
f) Permutation tests performed on markers of the MutSα complex - p-values reported for MSH2 are from this permutation set
g) Permutation tests performed on markers of the MutSβ complex
h) Permutation tests performed on markers of the MutSγ complex
g) no chi-squared permutations exceeded those observed for SNP rs805304
