# Supplementary material for: Unique DNA Repair Gene Variations and Potential Associations with the Primary Antibody Deficiency Syndromes IgAD and CVID
Source: PLoS One. 2010 Aug 18;5(8):e12260. doi: 10.1371/journal.pone.0012260 (PMC2923613; doi:10.1371/journal.pone.0012260)
Supplement: Table S2 — (0.04 MB PDF) [file pone.0012260.s002.pdf]

Table S2. Haplotype block structure obtained from control genotypes.

Gene	Haplotype block	SNPs in block					
BLM	1	rs8034371	rs6496724	rs7183841			
BLM	2	rs3815003	rs8036601	rs7175811			
BLM	3	rs7182287	rs2270132	rs2073919			
DMC1	4	rs1946990	rs5750616	rs1980455			
DMC1	5	rs5757130	rs5757133	rs5757135	rs5757141	rs8140617	rs1569492
ERCC1	6	rs11615	rs1319052				
EXO1	7	rs1635515	rs2526700	rs1776133	rs2526698	rs735943	rs851781
H2AFX	8	rs643788	rs649870				
LIG1	9	rs156633	rs156641	rs2288878			
LIG1	10	rs274869	rs274873				
MLH1	11	rs9852378	rs4647224	rs4647250	rs1558528	rs2286939	rs2286940
		rs2241031					
MLH3	12	rs175047	rs108622	rs175057	rs2098252	rs735452	rs3742780
		rs10136948	rs10142770				
MRE11	13	rs2155209	rs661957				
MRE11	14	rs682213	rs569143				
MRE11	15	rs497763	rs472344				
MSH2	16	rs2347794	rs3771274	rs6726691	rs1981928	rs3771275	rs3771276
		rs6729015	rs3771281				
MSH3	17	rs1650670	rs1650666	rs1650663	rs1677649	rs6864493	rs3776968
		rs26282	rs33013	rs6151838			
MSH3	18	rs245391	rs32991	rs26910			
MSH3	19	rs245341	rs27887	rs32964			
MSH4	20	rs5745325	rs5745327	rs1146652	rs1144342	rs5745390	rs1565717
		rs5745458	rs1001160	rs5745543	rs5745545	rs946163	
MSH5	21	rs2075788	rs3117572	rs2299851	rs3131379	rs3131378	rs3117577
		rs707938					
MUS81	22	rs635375	rs652021	rs665306	rs630303	rs659824	
RAD50	23	rs2244012	rs2706348	rs2252775	rs2237060	rs2240032	rs2158177
RAD51	24	rs2412545	rs2619681	rs2412546	rs11858337	rs957603	rs11070291
		rs4924501					
RAD52	25	rs10744729	rs4766370	rs9634161	rs4766377	rs1833095	
RAD54B	26	rs2046666	rs2470740	rs2046663	rs3019149	rs3136421	rs3019279
		rs2921385					
TP53BP1	27	rs542898	rs2602141	rs536313	rs2467739	rs2467741	rs694725
		rs689647	rs560191	rs2439850	rs1869258	rs523156	
XRCC2	28	rs3111471	rs3218408				
