# Supplementary material for: Unique DNA Repair Gene Variations and Potential Associations with the Primary Antibody Deficiency Syndromes IgAD and CVID
Source: PLoS One. 2010 Aug 18;5(8):e12260. doi: 10.1371/journal.pone.0012260 (PMC2923613; doi:10.1371/journal.pone.0012260)
Supplement: Table S3 — (0.09 MB PDF) [file pone.0012260.s003.pdf]

Table S3. Haplotype association for SNPs genotyped in IgAD and CVID. *p*-values (<0.05) are highlighted in bold.

	IgAD				CVID				Combined CVID & IgAD			
	Hap Freq.	Case, Control Frequencies	p-value	Corrected p-value [a]	Hap Freq.	Case, Control Frequencies	P Value	Corrected p-value [a]	Hap Freq.	Case, Control Frequencies	P Value	Corrected p-value [a]
BLM[g]												
Block 1												
ATT	0.702	0.693, 0.716	0.59	1.00	0.705	0.685, 0.716	0.59	1.00	0.699	0.691, 0.716	0.54	1.00
TGC	0.206	0.211, 0.200	0.78	1.00	0.203	0.208, 0.200	0.87	1.00	0.207	0.210, 0.200	0.78	1.00
AGT	0.065	0.071, 0.055	0.49	1.00	0.059	0.065, 0.055	0.76	1.00	0.065	0.070, 0.055	0.52	1.00
TGT	0.022	0.025, 0.017	0.58	1.00	0.021	0.031, 0.016	0.43	1.00	0.023	0.026, 0.017	0.48	1.00
TTT	--	--	--	--	0.005	0.011, 0.001	0.24	1.00	0.002	0.003, 0.001	0.57	1.00
ATC	0.002	0.000, 0.006	0.20	1.00	0.004	0.000, 0.006	0.45	1.00	0.002	0.000, 0.006	0.14	1.00
AGC	0.002	0.000, 0.005	0.25	1.00	0.003	0.000, 0.005	0.49	1.00	0.002	0.000, 0.005	0.18	1.00
BLM[g]												
Block 2												
ACC	0.575	0.583, 0.563	0.68	1.00	0.571	0.583, 0.564	0.77	1.00	0.576	0.583, 0.564	0.67	1.00
GTT	0.262	0.268, 0.253	0.71	1.00	0.267	0.291, 0.254	0.51	1.00	0.267	0.274, 0.253	0.60	1.00
ATT	0.091	0.073, 0.119	0.09	0.97	0.105	0.081, 0.118	0.35	1.00	0.089	0.075, 0.118	0.09	0.97
ACT	0.038	0.033, 0.046	0.50	1.00	0.041	0.033, 0.046	0.60	1.00	0.037	0.033, 0.046	0.46	1.00
GCC	0.014	0.020, 0.007	0.25	1.00	0.004	0.000, 0.006	0.50	1.00	0.012	0.015, 0.006	0.40	1.00
ATC	0.009	0.012, 0.006	0.53	1.00	0.008	0.011, 0.006	0.66	1.00	0.01	0.011, 0.006	0.54	1.00
GCT	0.008	0.008, 0.007	0.88	1.00	0.005	0.002, 0.006	0.57	1.00	0.007	0.006, 0.007	0.98	1.00
GTC	0.002	0.003, 0.000	0.45	1.00	--	--	--	--	0.002	0.003, 0.000	0.50	1.00
BLM[g]												
Block 3												
CCG	0.409	0.415, 0.400	0.75	1.00	0.386	0.363, 0.398	0.57	1.00	0.4	0.401, 0.399	0.96	1.00
CAG	0.365	0.375, 0.350	0.59	1.00	0.364	0.387, 0.352	0.56	1.00	0.37	0.379, 0.351	0.53	1.00
TAA	0.224	0.207, 0.250	0.28	1.00	0.244	0.238, 0.248	0.85	1.00	0.226	0.214, 0.249	0.36	1.00
TCA	--	--	--	--	0.006	0.012, 0.002	0.26	1.00	0.003	0.004, 0.001	0.58	1.00
TAG	0.002	0.004, 0.000	0.47	1.00	--	--	--	--	0.002	0.003, 0.000	0.53	1.00
DMC1[g]												
Block 4												
AAA	0.375	0.379, 0.369	0.83	1.00	0.373	0.380, 0.369	0.87	1.00	0.376	0.379, 0.369	0.82	1.00
GAC	0.347	0.332, 0.369	0.41	1.00	0.346	0.302, 0.369	0.26	1.00	0.339	0.324, 0.369	0.29	1.00
ACA	0.26	0.271, 0.245	0.53	1.00	0.259	0.287, 0.245	0.44	1.00	0.265	0.275, 0.245	0.45	1.00
AAC	0.009	0.011, 0.006	0.56	1.00	0.007	0.011, 0.006	0.65	1.00	0.009	0.011, 0.006	0.55	1.00
GAA	0.007	0.004, 0.011	0.33	1.00	0.009	0.006, 0.011	0.66	1.00	0.006	0.004, 0.011	0.33	1.00
GCA	0.002	0.004, 0.000	0.42	1.00	0.005	0.015, 0.000	0.10	0.97	0.005	0.007, 0.000	0.27	1.00
DMC1[g]												
Block 5												
GGGCCG	0.601	0.625, 0.565	0.20	1.00	0.596	0.655, 0.565	0.14	1.00	0.61	0.633, 0.565	0.12	1.00
AAATTA	0.308	0.296, 0.326	0.50	1.00	0.282	0.198, 0.325	**0.024**	0.47	0.289	0.271, 0.326	0.18	1.00
AGACCA	0.036	0.036, 0.038	0.91	1.00	0.046	0.063, 0.038	0.33	1.00	0.041	0.043, 0.038	0.79	1.00
GAGCCG	0.017	0.014, 0.022	0.54	1.00	0.018	0.011, 0.022	0.49	1.00	0.016	0.013, 0.022	0.45	1.00
GGGCCA	0.017	0.022, 0.011	0.40	1.00	0.011	0.010, 0.011	0.97	1.00	0.016	0.019, 0.011	0.50	1.00
AAATCA	0.007	0.004, 0.011	0.36	1.00	0.014	0.021, 0.011	0.51	1.00	0.009	0.008, 0.011	0.75	1.00
AAACTA	--	--	--	--	0.007	0.021, 0.000	0.060	0.89	0.004	0.005, 0.000	0.34	1.00
AGATCA	0.002	0.004, 0.000	0.43	1.00	0.003	0.010, 0.000	0.19	1.00	0.004	0.005, 0.000	0.33	1.00
GGATCG	--	--	--	--	0.004	0.010, 0.000	0.17	1.00	0.002	0.003, 0.000	0.48	1.00
AAATTG	0.002	0.000, 0.005	0.22	1.00	0.004	0.000, 0.005	0.47	1.00	0.002	0.000, 0.005	0.16	1.00
AGACCG	0.002	0.000, 0.006	0.21	1.00	0.004	0.000, 0.006	0.46	1.00	0.002	0.000, 0.006	0.15	1.00
GAATTA	0.002	0.000, 0.005	0.22	1.00	0.004	0.000, 0.006	0.47	1.00	0.002	0.000, 0.005	0.15	1.00
GGGTCG	0.002	0.000, 0.005	0.22	1.00	0.004	0.001, 0.006	0.56	1.00	0.002	0.000, 0.005	0.17	1.00
AGATTA	0.002	0.000, 0.006	0.23	1.00	0.002	0.000, 0.004	0.56	1.00	0.002	0.000, 0.006	0.16	1.00
GGATTA	--	--	--	--	0.001	0.000, 0.002	0.67	1.00	--	--	--	--
ERCC1												
Block 6												
TC	0.616	0.589, 0.657	0.14	nd	0.632	0.583, 0.657	0.22	nd	0.61	0.587, 0.657	0.11	nd
CT	0.337	0.364, 0.297	0.13	nd	0.326	0.384, 0.296	0.14	nd	0.345	0.369, 0.297	0.093	nd
CC	0.025	0.018, 0.035	0.26	nd	0.027	0.011, 0.035	0.24	nd	0.023	0.017, 0.035	0.17	nd
TT	0.022	0.029, 0.012	0.21	nd	0.015	0.023, 0.011	0.47	nd	0.022	0.028, 0.012	0.23	nd

Table S3. Haplotype association for SNPs genotyped in IgAD and CVID. *p*-values (<0.05) are highlighted in bold.

	IgAD				CVID				Combined CVID & IgAD			
	Hap Freq.	Case, Control Frequencies	p-value	Corrected p-value [a]	Hap Freq.	Case, Control Frequencies	P Value	Corrected p-value [a]	Hap Freq.	Case, Control Frequencies	P Value	Corrected p-value [a]
EXO1												
Block 7												
CTTGGT	0.476	0.468, 0.489	0.65	nd	0.464	0.417, 0.489	0.25	nd	0.468	0.457, 0.489	0.47	nd
TGCAAC	0.394	0.396, 0.391	0.91	nd	0.432	0.510, 0.391	0.056	nd	0.415	0.427, 0.391	0.41	nd
CGTAGC	0.101	0.111, 0.087	0.41	nd	0.064	0.021, 0.087	**0.032**	nd	0.088	0.089, 0.087	0.95	nd
CTTGGC	0.011	0.014, 0.005	0.37	nd	0.007	0.010, 0.005	0.64	nd	0.011	0.013, 0.005	0.39	nd
TTCAAC	0.004	0.007, 0.000	0.26	nd	--	--	--	--	0.004	0.005, 0.000	0.33	nd
CGCAAC	0.004	0.000, 0.011	0.079	nd	0.007	0.000, 0.011	0.31	nd	0.004	0.000, 0.011	**0.043**	nd
TGCAAT	0.004	0.000, 0.011	0.082	nd	0.007	0.000, 0.011	0.31	nd	0.004	0.000, 0.011	**0.045**	nd
CGTGGC	0.002	0.000, 0.005	0.22	nd	0.007	0.010, 0.005	0.64	nd	0.004	0.003, 0.005	0.61	nd
TTTGGT	--	--	--	--	0.004	0.010, 0.000	0.17	nd	0.002	0.003, 0.000	0.48	nd
CTTGAT	0.002	0.004, 0.000	0.43	nd	--	--	--	--	0.002	0.003, 0.000	0.49	nd
CTCGGT	--	--	--	--	0.004	0.010, 0.000	0.17	nd	--	--	--	--
TGTAAT	--	--	--	--	0.002	0.006, 0.000	0.31	nd	--	--	--	--
TGTAGT	--	--	--	--	0.002	0.005, 0.000	0.35	nd	--	--	--	--
H2AFX												
Block 8												
AA	0.623	0.621, 0.625	0.94	nd	0.596	0.541, 0.625	0.18	nd	0.609	0.601, 0.625	0.59	nd
GG	0.368	0.375, 0.359	0.72	nd	0.382	0.427, 0.358	0.27	nd	0.378	0.388, 0.359	0.50	nd
AG	0.004	0.004, 0.005	0.78	nd	0.015	0.032, 0.006	0.085	nd	0.009	0.011, 0.006	0.53	nd
GA	0.004	0.000, 0.011	0.084	nd	0.007	0.000, 0.011	0.31	nd	0.004	0.000, 0.011	**0.046**	nd
LIG1												
Block 9												
GCA	0.437	0.428, 0.450	0.64	nd	0.452	0.457, 0.450	0.90	nd	0.44	0.436, 0.450	0.74	nd
ATG	0.4	0.405, 0.393	0.81	nd	0.382	0.354, 0.397	0.48	nd	0.393	0.392, 0.397	0.91	nd
ACG	0.145	0.156, 0.128	0.41	nd	0.127	0.135, 0.124	0.79	nd	0.142	0.151, 0.124	0.41	nd
GTG	0.007	0.007, 0.006	0.83	nd	0.007	0.010, 0.006	0.66	nd	0.007	0.008, 0.006	0.75	nd
GCG	0.004	0.000, 0.011	0.081	nd	0.015	0.021, 0.012	0.53	nd	0.007	0.005, 0.011	0.46	nd
ATA	0.004	0.000, 0.011	0.087	nd	0.008	0.011, 0.007	0.79	nd	0.004	0.003, 0.007	0.44	nd
ACA	--	--	--	--	0.007	0.011, 0.004	0.49	nd	0.003	0.003, 0.004	0.84	nd
GTA	0.002	0.004, 0.000	0.47	nd	--	--	--	--	0.002	0.003, 0.000	0.53	nd
LIG1												
Block 10												
TA	0.526	0.518, 0.538	0.67	nd	0.528	0.510, 0.538	0.66	nd	0.523	0.516, 0.538	0.62	nd
CG	0.461	0.461, 0.462	0.98	nd	0.464	0.469, 0.462	0.92	nd	0.462	0.463, 0.462	0.99	nd
TG	0.009	0.014, 0.000	0.11	nd	0.007	0.021, 0.000	**0.049**	nd	0.011	0.016, 0.000	0.087	nd
CA	0.004	0.007, 0.000	0.27	nd	--	--	--	--	0.004	0.005, 0.000	0.34	nd
MLH1 [d]												
Block 11												
GCTCTGC	0.526	0.511, 0.549	0.42	1.00	0.525	0.490, 0.543	0.39	1.00	0.520	0.505, 0.549	0.33	1.00
ATCACAT	0.431	0.461, 0.385	0.11	0.68	0.414	0.479, 0.380	0.11	0.46	0.437	0.465, 0.379	0.054	0.53
ATTATAT	0.008	0.000, 0.020	**0.018**	**0.036**	0.013	0.000, 0.020	0.16	0.64	0.006	0.000, 0.020	**0.0062**	**0.014**
GCTCTGT	0.004	0.004, 0.005	0.76	1.00	--	--	--	--	0.005	0.003, 0.011	0.21	0.97
GCTATAT	--	--	--	--	0.004	0.000, 0.005	0.47	1.00	0.004	0.003, 0.005	0.60	1.00
ATTACAT	0.005	0.004, 0.008	0.55	1.00	0.005	0.000, 0.007	0.43	1.00	0.004	0.003, 0.008	0.40	1.00
ACTCTGC	0.004	0.007, 0.000	0.25	1.00	--	--	--	--	0.004	0.005, 0.000	0.33	1.00
GCCCCAT	0.004	0.007, 0.000	0.25	1.00	--	--	--	--	0.004	0.005, 0.000	0.32	1.00
ATCATGT	0.004	0.000, 0.011	0.079	0.32	0.007	0.000, 0.011	0.31	1.00	0.004	0.000, 0.011	**0.042**	0.25
GTCACAT	0.004	0.000, 0.011	0.081	0.38	0.007	0.000, 0.011	0.31	1.00	0.004	0.000, 0.011	**0.043**	0.28
ACTCCAT	--	--	--	--	--	--	--	--	0.002	0.003, 0.000	0.49	1.00
GCTATGC	--	--	--	--	0.004	0.010, 0.000	0.17	0.94	0.002	0.003, 0.000	0.49	1.00
GTTACGT	0.002	0.004, 0.000	0.42	1.00	--	--	--	--	0.002	0.003, 0.000	0.49	1.00
GTTCTGC	0.002	0.004, 0.000	0.42	1.00	--	--	--	--	0.002	0.003, 0.000	0.48	1.00
ATTACGT	0.002	0.000, 0.005	0.22	0.95	0.004	0.000, 0.005	0.47	1.00	0.002	0.000, 0.005	0.15	0.86
GCCATGC	0.001	0.000, 0.003	0.37	1.00	0.002	0.000, 0.003	0.60	1.00	--	--	--	--
GTCATGC	0.001	0.000, 0.003	0.39	1.00	0.002	0.000, 0.003	0.62	1.00	--	--	--	--
GCTCTAT	--	--	--	--	0.011	0.010, 0.011	0.97	1.00	--	--	--	--
ACTACAT	--	--	--	--	0.004	0.010, 0.000	0.17	0.92	--	--	--	--

Table S3. Haplotype association for SNPs genotyped in IgAD and CVID. *p*-values (<0.05) are highlighted in bold.

	IgAD				CVID				Combined CVID & IgAD			
	Hap Freq.	Case, Control Frequencies	p-value	Corrected p-value [a]	Hap Freq.	Case, Control Frequencies	P Value	Corrected p-value [a]	Hap Freq.	Case, Control Frequencies	P Value	Corrected p-value [a]
MLH3												
Block 12												
GCAAGCGC	0.444	0.432, 0.462	0.53	nd	0.486	0.531, 0.462	0.27	nd	0.461	0.457, 0.467	0.83	nd
CTGGAGAT	0.459	0.461, 0.456	0.93	nd	0.417	0.344, 0.455	0.073	nd	0.438	0.431, 0.454	0.60	nd
CTGGACGC	0.065	0.079, 0.043	0.13	nd	0.044	0.042, 0.045	0.90	nd	0.062	0.069, 0.046	0.28	nd
GTAGGCGC	0.006	0.011, 0.000	0.16	nd	0.005	0.016, 0.000	0.089	nd	0.011	0.016, 0.000	0.087	nd
CTGAAGAT	0.007	0.007, 0.005	0.81	nd	0.002	0.000, 0.003	0.60	nd	0.005	0.005, 0.005	1.00	nd
CTGGGGAT	--	--	--	--	0.008	0.021, 0.002	0.090	nd	0.004	0.005, 0.002	0.56	nd
CTGGAGAC	0.004	0.004, 0.005	0.76	nd	0.004	0.000, 0.005	0.47	nd	0.004	0.003, 0.006	0.60	nd
CTGAGGAT	--	--	--	--	0.004	0.010, 0.000	0.17	nd	0.002	0.003, 0.000	0.49	nd
GCAGGCGC	--	--	--	--	0.004	0.010, 0.000	0.17	nd	0.002	0.003, 0.000	0.49	nd
GCAAACGC	--	--	--	--	0.002	0.006, 0.000	0.30	nd	0.002	0.003, 0.000	0.49	nd
CTGGAGGT	0.002	0.004, 0.000	0.42	nd	--	--	--	--	0.002	0.003, 0.000	0.49	nd
GTAAACGC	0.002	0.004, 0.000	0.42	nd	--	--	--	--	0.002	0.003, 0.000	0.49	nd
CTAAAGGT	0.002	0.000, 0.005	0.22	nd	0.004	0.000, 0.005	0.47	nd	0.002	0.000, 0.005	0.15	nd
CTAAGCGC	0.002	0.000, 0.005	0.22	nd	0.004	0.000, 0.005	0.47	nd	0.002	0.000, 0.005	0.15	nd
GCAAAGAT	0.002	0.000, 0.005	0.22	nd	0.004	0.000, 0.005	0.47	nd	0.002	0.000, 0.005	0.15	nd
CTGGGCGC	0.002	0.000, 0.005	0.22	nd	0.003	0.000, 0.004	0.53	nd	0.001	0.000, 0.004	0.24	nd
GCGGGCGC	0.001	0.000, 0.003	0.38	nd	0.002	0.000, 0.003	0.61	nd	--	--	--	--
GCGGGGGC	0.001	0.000, 0.003	0.38	nd	0.002	0.000, 0.003	0.61	nd	--	--	--	--
GTAAGCGC	--	--	--	--	0.005	0.016, 0.000	0.089	nd	--	--	--	--
CCAAACGC	--	--	--	--	0.002	0.004, 0.000	0.37	nd	--	--	--	--
GTGAAGAT	--	--	--	--	0.001	0.000, 0.002	0.65	nd	--	--	--	--
MRE11 [e]												
Block 13												
AC	0.651	0.654, 0.646	0.86	1.00	0.636	0.617, 0.646	0.63	1.00	0.646	0.645, 0.646	0.97	1.00
GT	0.335	0.338, 0.331	0.88	1.00	0.348	0.383, 0.331	0.39	1.00	0.343	0.349, 0.331	0.67	1.00
GC	0.009	0.004, 0.017	0.15	0.90	0.011	0.000, 0.017	0.20	0.96	0.008	0.003, 0.017	0.075	0.72
AT	0.005	0.004, 0.006	0.76	1.00	0.004	0.000, 0.006	0.48	1.00	0.004	0.003, 0.006	0.61	1.00
MRE11 [e]												
Block 14												
TC	0.564	0.585, 0.532	0.25	1.00	0.571	0.646, 0.532	0.067	0.63	0.578	0.601, 0.532	0.12	0.87
CG	0.392	0.375, 0.418	0.36	1.00	0.385	0.323, 0.417	0.12	0.81	0.38	0.361, 0.418	0.20	0.99
CC	0.038	0.036, 0.040	0.85	1.00	0.037	0.032, 0.040	0.74	1.00	0.036	0.035, 0.039	0.79	1.00
TG	0.007	0.004, 0.011	0.34	1.00	0.008	0.000, 0.011	0.31	1.00	0.006	0.003, 0.011	0.21	0.99
MRE11 [e]												
Block 15												
GT	0.558	0.586, 0.516	0.14	0.88	0.56	0.645, 0.516	**0.039**	0.27	0.573	0.601, 0.516	0.057	0.62
AA	0.422	0.396, 0.462	0.16	0.93	0.421	0.343, 0.462	0.057	0.57	0.409	0.383, 0.462	0.075	0.72
AT	0.013	0.014, 0.011	0.78	1.00	0.011	0.011, 0.011	0.97	1.00	0.013	0.013, 0.011	0.83	1.00
GA	0.007	0.004, 0.011	0.33	1.00	0.007	0.000, 0.011	0.33	1.00	0.006	0.003, 0.011	0.21	0.99
MSH2 [b, c]												
Block 16												
GCGACGCG	0.575	0.543, 0.625	0.079	0.40	0.568	0.458, 0.625	**0.0075**	**0.025**	0.558	0.521, 0.633	**0.013**	0.066
ATCTGAAA	0.258	0.285, 0.217	0.10	0.74	0.25	0.311, 0.217	0.085	0.53	0.27	0.293, 0.222	0.077	0.57
ATGAGAAA	0.095	0.115, 0.065	0.075	0.39	0.086	0.126, 0.065	0.085	0.53	0.102	0.118, 0.067	0.059	0.47
ATGAGAAG	0.011	0.007, 0.016	0.32	1.00	0.025	0.041, 0.016	0.21	0.99	0.016	0.015, 0.017	0.91	1.00
GCGACGAG	0.015	0.018, 0.011	0.54	1.00	0.007	0.000, 0.011	0.31	1.00	0.013	0.013, 0.011	0.82	1.00
ATGACGCG	0.004	0.004, 0.005	0.76	1.00	0.011	0.021, 0.005	0.23	0.99	0.007	0.008, 0.006	0.75	1.00
GCGACAAG	0.004	0.004, 0.005	0.76	1.00	0.007	0.010, 0.005	0.64	1.00	0.005	0.005, 0.006	0.98	1.00
ATCTGAAG	0.005	0.004, 0.005	0.83	1.00	0.004	0.001, 0.005	0.58	1.00	0.004	0.003, 0.006	0.71	1.00
ATCAGAAA	0.004	0.007, 0.000	0.25	1.00	--	--	--	--	0.004	0.005, 0.000	0.33	1.00
GCGAGGCG	0.002	0.004, 0.000	0.41	1.00	0.004	0.010, 0.000	0.16	0.70	0.004	0.005, 0.000	0.32	1.00
GTCAGAAA	--	--	--	--	0.004	0.010, 0.000	0.17	0.94	0.002	0.003, 0.000	0.49	1.00
GTCTGACG	0.004	0.000, 0.011	0.080	0.65	0.007	0.000, 0.011	0.31	1.00	0.002	0.000, 0.006	0.15	0.75
GCCACGCA	0.002	0.004, 0.000	0.41	1.00	--	--	--	--	0.002	0.003, 0.000	0.48	1.00
GCGACACA	0.002	0.004, 0.000	0.42	1.00	--	--	--	--	0.002	0.003, 0.000	0.49	1.00
GTCACGCG	0.002	0.004, 0.000	0.42	1.00	--	--	--	--	0.002	0.003, 0.000	0.49	1.00
ACGACGCA	0.002	0.000, 0.005	0.22	0.98	0.004	0.000, 0.005	0.47	1.00	0.002	0.000, 0.006	0.15	0.75
ATGTGGAA	0.002	0.000, 0.005	0.22	0.98	0.004	0.000, 0.005	0.47	1.00	0.002	0.000, 0.006	0.15	0.75
GCGTGGCG	0.002	0.000, 0.005	0.22	0.98	0.004	0.000, 0.005	0.47	1.00	0.002	0.000, 0.006	0.15	0.75
GTCAGGCA	0.002	0.000, 0.005	0.22	0.98	0.004	0.000, 0.005	0.47	1.00	0.002	0.000, 0.006	0.15	0.75
GTGTGGCG	0.002	0.000, 0.005	0.22	0.98	0.004	0.000, 0.005	0.47	1.00	0.002	0.000, 0.006	0.15	0.75
ACCTGGCG	0.001	0.000, 0.003	0.39	1.00	0.002	0.000, 0.003	0.61	1.00	--	--	--	--
ACGAGAAA	0.001	0.000, 0.003	0.38	1.00	0.002	0.000, 0.003	0.61	1.00	--	--	--	--
ACGTGAAA	0.001	0.000, 0.003	0.39	1.00	0.002	0.000, 0.003	0.61	1.00	--	--	--	--
GCCTGGCG	0.001	0.000, 0.003	0.38	1.00	0.002	0.000, 0.003	0.61	1.00	--	--	--	--
GCCACGAG	--	--	--	--	0.002	0.005, 0.000	0.33	1.00	--	--	--	--
GCCAGGAG	--	--	--	--	0.002	0.005, 0.000	0.33	1.00	--	--	--	--

Table S3. Haplotype association for SNPs genotyped in IgAD and CVID. *p*-values (<0.05) are highlighted in bold.

	IgAD				CVID				Combined CVID & IgAD			
	Hap Freq.	Case, Control Frequencies	p-value	Corrected p-value [a]	Hap Freq.	Case, Control Frequencies	P Value	Corrected p-value [a]	Hap Freq.	Case, Control Frequencies	P Value	Corrected p-value [a]
MSH3 [c]												
Block 17												
TGTCTCGGT	0.292	0.286, 0.304	0.68	1.00	0.311	0.332, 0.301	0.60	1.00	0.3	0.298, 0.302	0.93	1.00
TGTCCTGAT	0.221	0.246, 0.186	0.13	0.97	0.224	0.301, 0.183	**0.026**	0.35	0.236	0.262, 0.184	**0.042**	0.55
CACTTCTGT	0.147	0.162, 0.125	0.27	1.00	0.115	0.094, 0.125	0.44	1.00	0.138	0.145, 0.124	0.51	1.00
TGTCTTGGT	0.127	0.125, 0.131	0.84	1.00	0.115	0.084, 0.131	0.24	1.00	0.118	0.112, 0.130	0.55	1.00
CACTTCTGC	0.093	0.089, 0.099	0.74	1.00	0.096	0.093, 0.097	0.91	1.00	0.093	0.091, 0.098	0.79	1.00
TGTCTCGAT	0.072	0.057, 0.096	0.12	0.97	0.073	0.031, 0.095	0.052	0.76	0.065	0.051, 0.095	**0.047**	0.77
TGTCCCGGT	0.006	0.002, 0.012	0.19	1.00	0.012	0.011, 0.012	0.91	1.00	0.007	0.004, 0.013	0.29	1.00
CACTTTTGT	0.006	0.010, 0.001	0.25	1.00	--	--	--	--	0.005	0.008, 0.001	0.32	1.00
TGTTTCTGT	0.006	0.000, 0.015	**0.044**	0.49	0.01	0.001, 0.014	0.26	1.00	0.005	0.000, 0.015	**0.022**	0.32
TGTTCCTGT	--	--	--	--	0.007	0.010, 0.005	0.67	1.00	0.004	0.004, 0.005	0.75	1.00
TGTCCCGAT	0.004	0.001, 0.010	0.13	0.97	0.007	0.001, 0.010	0.39	1.00	0.004	0.000, 0.010	0.086	0.94
CGTCTCTGT	0.004	0.004, 0.005	0.76	1.00	0.004	0.000, 0.005	0.47	1.00	0.004	0.003, 0.005	0.61	1.00
CGCTTCGGT	0.004	0.004, 0.005	0.77	1.00	0.001	0.000, 0.002	0.69	1.00	0.004	0.003, 0.005	0.61	1.00
TATCTCGGT	--	--	--	--	--	--	--	--	0.003	0.004, 0.000	0.37	1.00
CGTCCTGAT	--	--	--	--	0.004	0.010, 0.000	0.17	0.98	0.002	0.003, 0.000	0.48	1.00
TATTTCTGC	--	--	--	--	0.004	0.000, 0.005	0.50	1.00	0.002	0.000, 0.005	0.15	1.00
TGTCCTGAC	--	--	--	--	0.004	0.011, 0.000	0.19	1.00	0.002	0.003, 0.000	0.52	1.00
TGTTCCGAT	--	--	--	--	0.004	0.010, 0.000	0.17	1.00	0.002	0.003, 0.000	0.49	1.00
TGTCTCTGT	0.003	0.000, 0.007	0.16	0.99	0.005	0.000, 0.007	0.40	1.00	0.002	0.000, 0.007	0.10	0.96
TGTTTCGAT	0.002	0.004, 0.000	0.42	1.00	--	--	--	--	0.002	0.003, 0.000	0.48	1.00
TATCTTGGT	--	--	--	--	--	--	--	--	0.001	0.002, 0.000	0.58	1.00
TATCCTGAT	0.004	0.007, 0.000	0.25	1.00	--	--	--	--	0.001	0.002, 0.000	0.55	1.00
CATTTCTGC	0.001	0.000, 0.003	0.34	1.00	--	--	--	--	--	--	--	--
CGCCTCTGC	0.001	0.002, 0.000	0.57	1.00	--	--	--	--	--	--	--	--
TGCCTCTGC	0.001	0.002, 0.000	0.57	1.00	--	--	--	--	--	--	--	--
CACTTCGGT	--	--	--	--	0.003	0.000, 0.004	0.58	1.00	--	--	--	--
TATTTCGGT	--	--	--	--	0.003	0.010, 0.000	0.17	1.00	--	--	--	--
MSH3 [c]												
Block 18												
TGA	0.451	0.448, 0.456	0.87	1.00	0.416	0.342, 0.455	0.068	0.83	0.433	0.422, 0.456	0.44	1.00
TTA	0.282	0.266, 0.307	0.34	1.00	0.291	0.259, 0.308	0.39	1.00	0.278	0.264, 0.307	0.28	1.00
GTT	0.137	0.143, 0.128	0.64	1.00	0.171	0.252, 0.129	**0.010**	0.14	0.157	0.171, 0.128	0.19	1.00
GTA	0.126	0.142, 0.103	0.22	1.00	0.116	0.145, 0.101	0.27	1.00	0.13	0.143, 0.103	0.18	1.00
GGA	0.003	0.001, 0.006	0.34	1.00	0.005	0.002, 0.007	0.57	1.00	0.003	0.001, 0.006	0.26	1.00
MSH3 [c]												
Block 19												
GCT	0.722	0.711, 0.739	0.51	1.00	0.75	0.771, 0.739	0.56	1.00	0.73	0.726, 0.739	0.75	1.00
ATC	0.261	0.282, 0.228	0.20	1.00	0.218	0.198, 0.228	0.56	1.00	0.25	0.261, 0.228	0.40	1.00
GTC	0.009	0.000, 0.022	**0.014**	0.20	0.014	0.000, 0.022	0.14	0.95	0.007	0.000, 0.022	**0.0043**	0.075
GTT	0.002	0.004, 0.000	0.42	1.00	0.004	0.010, 0.000	0.17	1.00	0.004	0.005, 0.000	0.33	1.00
GCC	0.001	0.000, 0.003	0.40	1.00	0.007	0.010, 0.006	0.64	1.00	0.004	0.003, 0.005	0.60	1.00
ACT	0.002	0.004, 0.000	0.43	1.00	--	--	--	--	0.003	0.004, 0.000	0.41	1.00
ATT	0.002	0.000, 0.005	0.22	1.00	0.007	0.010, 0.005	0.64	1.00	0.003	0.001, 0.005	0.38	1.00
ACC	0.001	0.000, 0.003	0.37	1.00	--	--	--	--	--	--	--	--
MSH4 [f]												
Block 20												
CAGCTGAAAAG	0.384	0.393, 0.370	0.63	1.00	0.366	0.364, 0.370	0.93	1.00	0.38	0.385, 0.372	0.75	1.00
TAGCTGACTGA	0.264	0.257, 0.275	0.66	1.00	0.261	0.229, 0.279	0.37	1.00	0.259	0.251, 0.277	0.51	1.00
CAGCGAGAAAG	0.196	0.196, 0.195	0.99	1.00	0.193	0.187, 0.196	0.85	1.00	0.195	0.195, 0.196	0.96	1.00
CTATTGAAAAG	0.067	0.064, 0.071	0.79	1.00	0.064	0.052, 0.071	0.54	1.00	0.065	0.062, 0.071	0.67	1.00
CTGTTGAAAAG	0.022	0.018, 0.028	0.48	1.00	0.033	0.042, 0.028	0.55	1.00	0.025	0.024, 0.028	0.78	1.00
CAGCGAAAAAG	0.02	0.029, 0.006	0.076	0.62	0.018	0.042, 0.006	**0.031**	0.34	0.024	0.033, 0.006	**0.050**	0.65
CAGCTGGAAAG	0.008	0.004, 0.015	0.20	0.98	0.015	0.011, 0.017	0.69	1.00	0.009	0.006, 0.015	0.25	1.00
TAGCTGACTGG	0.004	0.007, 0.000	0.25	1.00	--	--	--	--	0.005	0.008, 0.000	0.22	1.00
CAGCGGAAAAG	0.004	0.007, 0.000	0.27	1.00	0.004	0.010, 0.000	0.19	1.00	0.005	0.008, 0.000	0.27	1.00
CAGCTGACTGA	0.004	0.007, 0.000	0.25	1.00	0.004	0.010, 0.000	0.17	0.99	0.005	0.008, 0.000	0.23	1.00
TAGCTGAAAAG	0.004	0.004, 0.006	0.76	1.00	0.004	0.000, 0.006	0.47	1.00	0.004	0.003, 0.006	0.60	1.00
TAGCTGACAGA	0.002	0.000, 0.005	0.22	1.00	0.007	0.010, 0.005	0.64	1.00	0.004	0.003, 0.005	0.61	1.00
CAGCGAGAAGG	--	--	--	--	0.004	0.010, 0.000	0.17	0.99	0.002	0.003, 0.000	0.48	1.00
CAGCTGAATAG	--	--	--	--	0.004	0.011, 0.000	0.19	1.00	0.002	0.003, 0.000	0.51	1.00
TAGCTGGCTGA	0.003	0.004, 0.002	0.75	1.00	--	--	--	--	0.002	0.003, 0.002	0.80	1.00
CAGCGAGCAAG	0.002	0.004, 0.000	0.42	1.00	--	--	--	--	0.002	0.003, 0.000	0.48	1.00
CAGCGGACTGA	0.002	0.004, 0.000	0.41	1.00	--	--	--	--	0.002	0.003, 0.000	0.48	1.00
TAGCTGAATGA	0.002	0.004, 0.000	0.42	1.00	--	--	--	--	0.002	0.003, 0.000	0.49	1.00
CAGCTGAAAGG	0.002	0.000, 0.005	0.22	1.00	0.004	0.000, 0.005	0.47	1.00	0.002	0.000, 0.005	0.15	0.97
CAGTTGAAAAG	0.002	0.000, 0.005	0.21	0.99	0.004	0.000, 0.006	0.46	1.00	0.002	0.000, 0.006	0.15	0.96
CTACTGAAAAG	0.002	0.000, 0.005	0.22	1.00	0.004	0.000, 0.005	0.47	1.00	0.002	0.000, 0.005	0.15	0.98
CAGCGGGAAAG	0.002	0.000, 0.005	0.23	1.00	0.003	0.000, 0.005	0.48	1.00	0.002	0.000, 0.005	0.17	1.00
TAGCTAAAAAG	0.001	0.000, 0.003	0.38	1.00	--	--	--	--	--	--	--	--
TAGCTAGAAAG	0.001	0.000, 0.003	0.39	1.00	--	--	--	--	--	--	--	--
TAGCGAGCTGG	--	--	--	--	0.004	0.010, 0.000	0.17	0.99	--	--	--	--
TAGCGGGCAAA	--	--	--	--	0.002	0.005, 0.000	0.33	1.00	--	--	--	--
TAGCGGGCTAA	--	--	--	--	0.002	0.005, 0.000	0.33	1.00	--	--	--	--

Table S3. Haplotype association for SNPs genotyped in IgAD and CVID. *p*-values (<0.05) are highlighted in bold.

	IgAD				CVID				Combined CVID & IgAD			
	Hap Freq.	Case, Control Frequencies	p-value	Corrected p-value [a]	Hap Freq.	Case, Control Frequencies	P Value	Corrected p-value [a]	Hap Freq.	Case, Control Frequencies	P Value	Corrected p-value [a]
MSH5 [f]												
Block 21												
TGCGATT	0.363	0.328, 0.418	**0.049**	0.46	0.412	0.399, 0.418	0.76	1.00	0.37	0.345, 0.422	0.077	0.78
TGCAGCC	0.243	0.307, 0.146	**7.5E-05**	**0.0010**	0.146	0.146, 0.145	0.99	1.00	0.227	0.266, 0.147	**0.0017**	**0.023**
TACGATT	0.139	0.091, 0.212	**2.0E-04**	**0.0033**	0.211	0.208, 0.212	0.94	1.00	0.15	0.121, 0.209	**0.0066**	0.095
GGTGATC	0.104	0.105, 0.103	0.95	1.00	0.102	0.100, 0.103	0.95	1.00	0.104	0.104, 0.104	0.98	1.00
GGCGATC	0.078	0.089, 0.060	0.25	1.00	0.065	0.073, 0.060	0.67	1.00	0.077	0.085, 0.061	0.31	1.00
TGCGATC	0.055	0.058, 0.050	0.72	1.00	0.044	0.031, 0.050	0.46	1.00	0.051	0.051, 0.051	0.98	1.00
TGCGGTT	0.002	0.004, 0.000	0.42	1.00	0.01	0.028, 0.000	**0.024**	0.28	0.007	0.011, 0.000	0.17	1.00
TGTGATT	0.005	0.004, 0.006	0.74	1.00	0.002	0.000, 0.003	0.60	1.00	0.004	0.003, 0.006	0.58	1.00
TGCAGCT	0.003	0.005, 0.000	0.35	1.00	--	--	--	--	0.003	0.004, 0.000	0.43	1.00
TGCGGCC	--	--	--	--	0.004	0.010, 0.000	0.17	1.00	0.002	0.003, 0.000	0.49	1.00
GACGATT	0.002	0.004, 0.000	0.43	1.00	--	--	--	--	0.002	0.003, 0.000	0.50	1.00
TACGACT	0.002	0.004, 0.000	0.43	1.00	--	--	--	--	0.002	0.003, 0.000	0.49	1.00
GGTAGCC	0.001	0.002, 0.000	0.52	1.00	--	--	--	--	0.001	0.002, 0.000	0.58	1.00
TACAACT	0.001	0.000, 0.003	0.38	1.00	0.002	0.000, 0.003	0.62	1.00	--	--	--	--
TACAATT	0.001	0.000, 0.003	0.38	1.00	0.002	0.000, 0.003	0.60	1.00	--	--	--	--
TATGATT	--	--	--	--	0.002	0.000, 0.003	0.64	1.00	--	--	--	--
GGTGGTC	--	--	--	--	0.001	0.003, 0.000	0.42	1.00	--	--	--	--
MUS81 [h]												
Block 22												
AACTG	0.504	0.489, 0.527	0.42	0.95	0.52	0.510, 0.525	0.80	1.00	0.504	0.494, 0.525	0.48	0.99
GGTCA	0.303	0.325, 0.269	0.20	0.61	0.269	0.276, 0.266	0.85	1.00	0.297	0.313, 0.266	0.26	0.91
AACTA	0.148	0.152, 0.142	0.75	1.00	0.14	0.139, 0.141	0.96	1.00	0.147	0.149, 0.141	0.81	1.00
AATTA	0.018	0.018, 0.018	0.97	1.00	0.018	0.019, 0.017	0.91	1.00	0.018	0.018, 0.017	0.94	1.00
AATTG	0.016	0.016, 0.017	0.91	1.00	0.02	0.024, 0.017	0.72	1.00	0.018	0.018, 0.017	0.95	1.00
AATCA	0.011	0.000, 0.027	**0.0053**	0.014	0.018	0.000, 0.027	0.11	0.49	0.009	0.000, 0.027	**0.0014**	0.0054
GGTTA	--	--	--	--	0.007	0.011, 0.005	0.63	1.00	0.004	0.003, 0.005	0.61	1.00
AGTCA	--	--	--	--	0.004	0.011, 0.000	0.17	0.74	0.002	0.003, 0.000	0.50	1.00
GGCCA	--	--	--	--	0.004	0.011, 0.000	0.19	0.83	0.002	0.003, 0.000	0.51	1.00
RAD50 [e]												
Block 23												
TGAGCA	0.365	0.398, 0.313	0.064	0.50	0.367	0.476, 0.310	**0.0062**	**0.041**	0.386	0.422, 0.311	**0.011**	0.097
TGATCA	0.4	0.384, 0.426	0.37	1.00	0.368	0.253, 0.429	**0.0038**	**0.024**	0.373	0.346, 0.428	0.061	0.63
CACTTG	0.19	0.177, 0.210	0.38	1.00	0.2	0.183, 0.209	0.60	1.00	0.189	0.179, 0.210	0.37	1.00
CACTTA	0.02	0.018, 0.022	0.73	1.00	0.026	0.032, 0.022	0.61	1.00	0.022	0.022, 0.022	0.97	1.00
TGATCG	--	--	--	--	0.007	0.021, 0.000	0.056	0.54	0.004	0.005, 0.000	0.33	1.00
TGCGCA	0.004	0.007, 0.000	0.25	1.00	--	--	--	--	0.004	0.005, 0.000	0.33	1.00
CACGCG	--	--	--	--	0.005	0.010, 0.003	0.40	1.00	0.003	0.003, 0.003	0.99	1.00
CACGTG	0.004	0.002, 0.007	0.36	1.00	0.006	0.003, 0.008	0.64	1.00	0.003	0.001, 0.007	0.30	1.00
CACTCG	0.004	0.000, 0.011	0.08	0.65	0.006	0.000, 0.008	0.40	1.00	0.003	0.000, 0.008	0.084	0.77
TGATTA	0.002	0.000, 0.005	0.25	0.99	0.007	0.010, 0.005	0.61	1.00	0.003	0.003, 0.005	0.64	1.00
CGCTTG	--	--	--	--	--	--	--	--	0.002	0.004, 0.000	0.43	1.00
TGAGTG	--	--	--	--	0.003	0.010, 0.000	0.17	0.89	0.002	0.003, 0.000	0.48	1.00
CAATCA	0.002	0.004, 0.000	0.42	1.00	--	--	--	--	0.002	0.003, 0.000	0.48	1.00
TGAGCG	0.002	0.004, 0.000	0.42	1.00	--	--	--	--	0.002	0.003, 0.000	0.50	1.00
TGATTG	0.002	0.000, 0.006	0.19	0.97	0.004	0.001, 0.006	0.52	1.00	0.002	0.000, 0.006	0.16	0.94
CGCGTG	0.004	0.007, 0.000	0.25	1.00	--	--	--	--	0.001	0.002, 0.000	0.56	1.00
RAD51 [g]												
Block 24												
GCGGTAG	0.387	0.402, 0.365	0.43	1.00	0.36	0.343, 0.368	0.68	1.00	0.379	0.387, 0.364	0.60	1.00
ACAACGT	0.351	0.328, 0.386	0.21	1.00	0.396	0.416, 0.386	0.62	1.00	0.362	0.351, 0.386	0.42	1.00
GTAACGT	0.151	0.161, 0.136	0.46	1.00	0.128	0.114, 0.136	0.62	1.00	0.144	0.149, 0.136	0.68	1.00
GCGGCAG	0.061	0.066, 0.053	0.57	1.00	0.054	0.063, 0.050	0.66	1.00	0.062	0.065, 0.054	0.61	1.00
GCAGTAG	0.012	0.014, 0.008	0.55	1.00	0.006	0.010, 0.003	0.45	1.00	0.012	0.013, 0.010	0.72	1.00
GCAACGT	0.004	0.004, 0.005	0.76	1.00	0.014	0.031, 0.006	0.085	0.96	0.009	0.011, 0.005	0.54	1.00
ACGGTAG	0.006	0.004, 0.011	0.34	1.00	0.007	0.000, 0.011	0.31	1.00	0.005	0.003, 0.011	0.21	1.00
GCGGTAT	0.004	0.004, 0.005	0.76	1.00	0.004	0.000, 0.005	0.47	1.00	0.004	0.003, 0.005	0.61	1.00
GTAATGG	0.003	0.004, 0.000	0.38	1.00	--	--	--	--	0.002	0.003, 0.000	0.44	1.00
ACAGCGT	0.002	0.003, 0.000	0.43	1.00	--	--	--	--	0.002	0.003, 0.000	0.49	1.00
GCGATAG	0.002	0.004, 0.000	0.42	1.00	--	--	--	--	0.002	0.003, 0.000	0.49	1.00
ACAACGG	0.001	0.002, 0.000	0.56	1.00	--	--	--	--	0.002	0.003, 0.000	0.48	1.00
GCAGCAG	0.003	0.000, 0.008	0.15	1.00	0.009	0.000, 0.013	0.26	1.00	0.002	0.000, 0.007	0.13	1.00
GTAACAG	--	--	--	--	0.004	0.010, 0.000	0.17	1.00	0.002	0.003, 0.000	0.48	1.00
GTAATGT	--	--	--	--	0.004	0.011, 0.000	0.19	1.00	0.002	0.003, 0.000	0.50	1.00
ACGACGT	0.002	0.000, 0.005	0.22	1.00	0.004	0.000, 0.005	0.48	1.00	0.002	0.000, 0.005	0.16	1.00
ACGGCGT	0.002	0.000, 0.005	0.25	1.00	0.004	0.000, 0.005	0.47	1.00	0.002	0.000, 0.005	0.17	1.00
GCGGCGG	0.002	0.000, 0.005	0.22	1.00	0.004	0.000, 0.005	0.47	1.00	0.002	0.000, 0.005	0.15	1.00
GCGGTGG	0.002	0.000, 0.005	0.22	1.00	0.004	0.000, 0.005	0.47	1.00	0.002	0.000, 0.005	0.15	1.00
GTAACGG	0.002	0.003, 0.000	0.46	1.00	--	--	--	--	0.001	0.002, 0.000	0.53	1.00
ACGACGG	0.001	0.002, 0.000	0.58	1.00	--	--	--	--	--	--	--	--

Table S3. Haplotype association for SNPs genotyped in IgAD and CVID. *p*-values (<0.05) are highlighted in bold.

	IgAD				CVID				Combined CVID & IgAD			
	Hap Freq.	Case, Control Frequencies	p-value	Corrected p-value [a]	Hap Freq.	Case, Control Frequencies	P Value	Corrected p-value [a]	Hap Freq.	Case, Control Frequencies	P Value	Corrected p-value [a]
RAD52 [g]												
Block 25												
TTTAC	0.257	0.255, 0.260	0.90	1.00	0.256	0.248, 0.260	0.83	1.00	0.255	0.253, 0.260	0.86	1.00
TTTAT	0.262	0.248, 0.283	0.40	1.00	0.247	0.178, 0.283	0.054	0.86	0.248	0.231, 0.283	0.18	1.00
GATGT	0.202	0.200, 0.205	0.90	1.00	0.184	0.146, 0.204	0.23	1.00	0.192	0.186, 0.205	0.60	1.00
GACAT	0.171	0.184, 0.151	0.37	1.00	0.193	0.271, 0.152	**0.017**	0.37	0.186	0.202, 0.151	0.14	1.00
GATAT	0.096	0.096, 0.094	0.93	1.00	0.108	0.134, 0.094	0.30	1.00	0.104	0.109, 0.094	0.59	1.00
GACAC	0.006	0.009, 0.001	0.24	1.00	--	--	--	--	0.007	0.010, 0.001	0.21	1.00
GTTGT	0.004	0.004, 0.006	0.76	1.00	--	--	--	--	0.004	0.003, 0.005	0.60	1.00
GTTAC	0.002	0.004, 0.000	0.42	1.00	--	--	--	--	0.002	0.003, 0.000	0.48	1.00
GTCAT	--	--	--	--	0.004	0.011, 0.000	0.16	1.00	0.002	0.003, 0.000	0.48	1.00
GATAC	--	--	--	--	0.005	0.012, 0.001	0.20	1.00	--	--	--	--
TTTGT	--	--	--	--	0.004	0.000, 0.006	0.48	1.00	--	--	--	--
RAD54B [g]												
Block 26												
TAGGGCT	0.508	0.457, 0.587	**0.0062**	0.15	0.55	0.479, 0.587	0.086	0.96	0.503	0.463, 0.587	**0.0057**	0.16
CTGAGGC	0.292	0.331, 0.233	**0.023**	0.52	0.262	0.317, 0.233	0.13	0.99	0.296	0.326, 0.233	**0.023**	0.56
CTAGTGC	0.06	0.064, 0.054	0.66	1.00	0.059	0.069, 0.054	0.63	1.00	0.062	0.066, 0.054	0.58	1.00
CTGGGCC	0.05	0.047, 0.054	0.73	1.00	0.043	0.021, 0.054	0.19	1.00	0.045	0.040, 0.054	0.46	1.00
CTAGGGC	0.041	0.050, 0.027	0.23	1.00	0.032	0.042, 0.027	0.51	1.00	0.041	0.048, 0.027	0.25	1.00
TAGGGGC	0.007	0.007, 0.005	0.81	1.00	0.014	0.031, 0.005	0.084	0.95	0.01	0.013, 0.005	0.42	1.00
TTGAGGC	0.008	0.006, 0.011	0.55	1.00	0.007	0.000, 0.011	0.30	1.00	0.007	0.004, 0.011	0.34	1.00
CTGAGGT	0.002	0.000, 0.005	0.23	1.00	0.009	0.016, 0.005	0.36	1.00	0.006	0.006, 0.006	1.00	1.00
TAGGGGT	0.007	0.011, 0.000	0.16	1.00	--	--	--	--	0.005	0.008, 0.000	0.23	1.00
CTGGGCT	0.007	0.007, 0.006	0.87	1.00	0.004	0.000, 0.006	0.46	1.00	0.005	0.005, 0.006	0.94	1.00
TAGAGGC	0.003	0.004, 0.000	0.37	1.00	--	--	--	--	0.003	0.004, 0.000	0.39	1.00
CTGATGC	0.003	0.004, 0.000	0.36	1.00	--	--	--	--	0.002	0.003, 0.000	0.44	1.00
CTGGTCC	0.002	0.003, 0.000	0.52	1.00	--	--	--	--	0.002	0.002, 0.000	0.58	1.00
CTGGTGC	0.002	0.004, 0.000	0.42	1.00	--	--	--	--	0.002	0.003, 0.000	0.48	1.00
TTGGGCC	0.002	0.004, 0.000	0.42	1.00	--	--	--	--	0.002	0.003, 0.000	0.45	1.00
CAGAGGC	--	--	--	--	0.004	0.010, 0.000	0.17	1.00	0.002	0.003, 0.000	0.49	1.00
TTGAGCC	--	--	--	--	0.004	0.010, 0.000	0.17	1.00	0.002	0.002, 0.000	0.52	1.00
CAAGGGC	0.002	0.000, 0.005	0.22	1.00	0.004	0.000, 0.005	0.47	1.00	0.002	0.000, 0.005	0.15	1.00
TAGAGCT	0.002	0.000, 0.005	0.22	1.00	0.004	0.000, 0.005	0.47	1.00	0.002	0.000, 0.005	0.16	1.00
TTGGGCT	0.002	0.000, 0.005	0.24	1.00	0.004	0.000, 0.005	0.47	1.00	0.002	0.000, 0.005	0.17	1.00
CTAGTGT	--	--	--	--	0.001	0.004, 0.000	0.37	1.00	--	--	--	--
TP53BP1												
Block 27												
GTCGTTCCCTG	0.636	0.626, 0.652	0.56	nd	0.661	0.677, 0.652	0.68	nd	0.645	0.642, 0.652	0.81	nd
AGTGCCCGCGC	0.194	0.185, 0.206	0.57	nd	0.2	0.187, 0.206	0.71	nd	0.193	0.187, 0.206	0.58	nd
AGTACCTGGGC	0.084	0.100, 0.060	0.13	nd	0.061	0.062, 0.060	0.93	nd	0.079	0.088, 0.060	0.24	nd
GTCGTTCGCTG	0.019	0.021, 0.016	0.69	nd	0.018	0.021, 0.016	0.76	nd	0.02	0.022, 0.016	0.68	nd
GTCGCTCCCTG	0.019	0.025, 0.011	0.28	nd	0.007	0.000, 0.011	0.31	nd	0.016	0.019, 0.011	0.48	nd
AGTACCTGGGG	0.011	0.014, 0.005	0.37	nd	0.014	0.031, 0.005	0.084	nd	0.014	0.019, 0.005	0.21	nd
GTCATTCCCTG	0.006	0.010, 0.000	0.17	nd	--	--	--	--	0.005	0.008, 0.000	0.23	nd
AGCGTTCCCTG	0.004	0.004, 0.005	0.76	nd	0.004	0.000, 0.005	0.47	nd	0.004	0.003, 0.005	0.61	nd
AGTACTTGGGG	0.004	0.004, 0.005	0.76	nd	0.004	0.000, 0.005	0.47	nd	0.004	0.003, 0.005	0.61	nd
GTCGTTCCCGG	0.004	0.004, 0.005	0.77	nd	0.004	0.000, 0.005	0.47	nd	0.004	0.003, 0.005	0.61	nd
GTCACTCCCTG	--	--	--	--	0.004	0.010, 0.000	0.17	nd	0.002	0.002, 0.000	0.50	nd
ATCACCTGGGG	0.002	0.004, 0.000	0.42	nd	--	--	--	--	0.002	0.003, 0.000	0.48	nd
ATCGTTCCCTC	0.002	0.004, 0.000	0.42	nd	--	--	--	--	0.002	0.003, 0.000	0.48	nd
AGCGCCCGCGC	0.002	0.000, 0.006	0.21	nd	0.004	0.000, 0.006	0.46	nd	0.002	0.000, 0.006	0.15	nd
AGTACCTGGTC	0.002	0.000, 0.006	0.21	nd	0.004	0.000, 0.006	0.47	nd	0.002	0.000, 0.006	0.15	nd
AGTGCCCGCTG	0.002	0.000, 0.005	0.22	nd	0.004	0.000, 0.005	0.47	nd	0.002	0.000, 0.005	0.15	nd
GTCGTTCCCGC	0.002	0.000, 0.005	0.22	nd	0.004	0.000, 0.005	0.47	nd	0.002	0.000, 0.005	0.15	nd
GTCGTTCCGTG	0.002	0.000, 0.005	0.22	nd	0.004	0.000, 0.005	0.47	nd	0.002	0.000, 0.005	0.16	nd
AGTGCCTGGTC	0.002	0.000, 0.005	0.23	nd	0.003	0.000, 0.005	0.48	nd	0.002	0.000, 0.005	0.16	nd
AGTGCTTGCGC	--	--	--	--	0.002	0.005, 0.000	0.33	nd	--	--	--	--
AGTGCTTGGGC	--	--	--	--	0.002	0.005, 0.000	0.33	nd	--	--	--	--
XRCC2												
Block 28												
CT	0.486	0.463, 0.521	0.23	nd	0.5	0.458, 0.522	0.31	nd	0.481	0.462, 0.521	0.19	nd
TT	0.264	0.279, 0.240	0.35	nd	0.257	0.292, 0.239	0.34	nd	0.269	0.283, 0.240	0.28	nd
TG	0.247	0.253, 0.238	0.72	nd	0.243	0.250, 0.239	0.84	nd	0.247	0.252, 0.238	0.73	nd
CG	0.003	0.004, 0.001	0.49	nd	--	--	--	--	0.003	0.003, 0.001	0.55	nd

a) p-value corrected for multiple testing bias by 100,000 permutations of all markers within pathways as described in Materials and Methods
b) Permutation tests performed on haplotypes (in blocks) of the MutSα complex - p-values reported for MSH2 are from this permutation set
c) Permutation tests performed on haplotypes (in blocks) of the MutSβ complex
d) Permutation tests performed on haplotypes (in blocks) of the MutLα complex
e) Permutation tests performed on haplotypes (in blocks) of the MRN complex
f) Permutation tests performed on haplotypes (in blocks) of the MutSγ complex
g) Permutation tests performed on haplotypes (in blocks) of the RAD52 extended epistasis group
h) Permutation tests performed on haplotypes (in blocks) in MUS81
