# Supplementary material for: Unique DNA Repair Gene Variations and Potential Associations with the Primary Antibody Deficiency Syndromes IgAD and CVID
Source: PLoS One. 2010 Aug 18;5(8):e12260. doi: 10.1371/journal.pone.0012260 (PMC2923613; doi:10.1371/journal.pone.0012260)
Supplement: Table S4 — (0.07 MB PDF) [file pone.0012260.s004.pdf]

Table S4. SNPs identified by resequencing.

Gene	Variation [a]	Detection Category [b]	Position (Build 36)	Classification	Novel [c]
AID	rs104894981	1	Chr12:8646213	3'UTR	√
AID	rs11324989	3	Chr12:8646692	3'UTR	-
AID	rs104894982	1	Chr12:8647701	3'UTR	√
AID	rs12307097	1	Chr12:8647838	3'UTR	-
AID	rs11046349	1	Chr12:8648058	3'UTR	-
AID	rs2028373	1	Chr12:8648748	SYN	-
AID	rs104894983	1	Chr12:8649100	SYN	√
AID	rs104894984	1	Chr12:8649295	SYN	√
AID	rs2518144	1	Chr12:8650712	Intron	-
AID	rs104894985	1	Chr12:8650860	SYN	√
APEX1	rs1760944	1	Chr14:19992989	5' flanking	-
APEX1	rs104894986	1	Chr14:19993136	5'UTR	√
APEX1	rs3136814	1	Chr14:19993137	5'UTR	-
APEX1	rs11622131	1	Chr14:19993163	5'UTR	-
APEX1	rs41561214	1	Chr14:19993256	5'UTR	-
APEX1	rs2307490	1	Chr14:19993380	5'UTR	-
APEX1	rs2307485	1	Chr14:19993571	Intron	-
APEX1	rs1048945	1	Chr14:19994007	Q51H	-
APEX1	rs17111967	1	Chr14:19994165	Intron	-
APEX1	rs41541122	1	Chr14:19994632	Intron	-
APEX1	rs104894987	1	Chr14:19994991	SYN	√
APEX1	rs1130409	1	Chr14:19994994	D148E	-
ERCC1	rs104894988	1	Chr19:50608418	3' flanking	√
ERCC1	rs3212978	1	Chr19:50608427	3' flanking	-
ERCC1	rs104894989	1	Chr19:50608997	Intron	√
ERCC1	rs41558212	1	Chr19:50610085	Intron	-
ERCC1	rs41561512	1	Chr19:50610095	Intron	-
ERCC1	rs3212961	1	Chr19:50614163	Intron	-
ERCC1	rs104894990	1	Chr19:50614306	Intron	√
ERCC1	rs3212955	1	Chr19:50615336	Intron	-
ERCC1	rs11615	1	Chr19:50615493	SYN	-
ERCC1	rs104894991	1	Chr19:50615648	Intron	√
ERCC1	rs3212948	1	Chr19:50616202	Intron	-
ERCC1	rs3212947	1	Chr19:50616372	SYN	-
ERCC1	rs104894992	1	Chr19:50616564	Intron	√
ERCC1	rs104894993	3	Chr19:50616574	Intron	√
ERCC1	rs2298881	1	Chr19:50618756	Intron	-
ERCC1	rs41559012	1	Chr19:50618890	5'UTR	-
ERCC1	rs3212931	2	Chr19:50619155	5' flanking	-
MLH1	rs1800734	1	Chr3:37009950	5' flanking	-
MLH1	rs56198082	1	Chr3:37010015	5'UTR	-
MLH1	rs104894994	1	Chr3:37010036	5'UTR	√
MLH1	rs104894995	1	Chr3:37013005	Intron	√
MLH1	rs4234259	1	Chr3:37023637	Intron	-
MLH1	rs4647255	1	Chr3:37025258	Intron	-
MLH1	rs1799977	1	Chr3:37028572	I219V	-
MLH1	rs104894996	1	Chr3:37030988	S247A	√
MLH1	rs104894998	1	Chr3:37033922	Intron	√

Table S4. SNPs identified by resequencing.

Gene	Variation [a]	Detection Category [b]	Position (Build 36)	Classification	Novel [c]
MLH1	rs104894999	1	Chr3:37036724	Intron	√
MLH1	rs2286939	1	Chr3:37037044	Intron	-
MLH1	rs104895000	1	Chr3:37042319	Q409P	√
MLH1	rs2286940	1	Chr3:37045110	Intron	-
MLH1	rs41562513	1	Chr3:37045441	Intron	-
MLH1	rs104895001	1	Chr3:37058728	Intron	√
MLH1	rs9876116	1	Chr3:37058744	Intron	-
MLH1	SWISS:VAR_004462	1	Chr3:37064134-5	K618A	-
MLH1	rs63750549	1	Chr3:37065027	G638R	√
MLH1	rs1800146	1	Chr3:37065074	SYN	-
MLH1	rs2241031	1	Chr3:37065278	Intron	-
MLH1	rs35831931	1	Chr3:37067023	V716M	-
MLH1	rs104895002	1	Chr3:37067057	H727L	√
MLH1	rs104895003	3	Chr3:37067429	3' flanking	√
MRE11	rs13447762	1	Chr11:93790066	3' flanking	-
MRE11	rs2155209	1	Chr11:93790438	3'UTR	-
MRE11	rs104895004	1	Chr11:93791006	3'UTR	√
MRE11	rs104895005	1	Chr11:93791163	3'UTR	√
MRE11	rs104895006	2	Chr11:93791193	3'UTR	√
MRE11	rs104895007	1	Chr11:93791711	3'UTR	√
MRE11	rs104895008	3	Chr11:93792254	3'UTR	√
MRE11	rs591959	3	Chr11:93792279	3'UTR	-
MRE11	rs11020777	1	Chr11:93792281	3'UTR	-
MRE11	rs104895009	1	Chr11:93792283	3'UTR	√
MRE11	rs13447749	1	Chr11:93792428	3'UTR	-
MRE11	rs104895010	1	Chr11:93792508	3'UTR	√
MRE11	rs104895011	1	Chr11:93792725	3'UTR	√
MRE11	rs104895012	1	Chr11:93802894	Intron	√
MRE11	rs104895013	1	Chr11:93802911	Intron	√
MRE11	rs1014666	1	Chr11:93818773	Intron	-
MRE11	rs104895014	1	Chr11:93818823	Intron	√
MRE11	rs13447696	1	Chr11:93819873	Intron	-
MRE11	rs13447695	1	Chr11:93819914	Intron	-
MRE11	rs104895015	1	Chr11:93832054	Intron	√
MRE11	rs104895016	1	Chr11:93832242	E494K	√
MRE11	rs61749249	1	Chr11:93832247	A492D	-
MRE11	rs529126	1	Chr11:93833951	Intron	-
MRE11	rs641936	1	Chr11:93836908	Intron	-
MRE11	rs640627	1	Chr11:93837216	Intron	-
MRE11	rs104895017	1	Chr11:93837270	Intron	√
MRE11	rs104895018	1	Chr11:93840496	Intron	√
MRE11	rs610611	1	Chr11:93843516	Intron	-
MRE11	rs104895019	1	Chr11:93844302	Intron	√
MRE11	rs13447623	1	Chr11:93848996	Intron	-
MRE11	rs535801	1	Chr11:93851696	Intron	-
MRE11	rs680695	1	Chr11:93851802	Intron	-
MRE11	rs104895020	1	Chr11:93863879	Intron	√
MRE11	rs496797	1	Chr11:93865455	Intron	-
MRE11	rs497763	1	Chr11:93865568	Intron	-
MRE11	rs1805363	1	Chr11:93866600	5'UTR	-
MRE11	rs11020802	1	Chr11:93866773	5' flanking	-

Table S4. SNPs identified by resequencing.

Gene	Variation [a]	Detection Category [b]	Position (Build 36)	Classification	Novel [c]
MSH2	rs2303425	1	Chr2:47483717	5' flanking	-
MSH2	rs2303426	1	Chr2:47484054	Intron	-
MSH2	rs3815865	1	Chr2:47484143	Intron	-
MSH2	rs17217758	2	Chr2:47490569	Intron	-
MSH2	rs104895021	1	Chr2:47490629	Intron	√
MSH2	rs17217765	1	Chr2:47490651	Intron	-
MSH2	rs104895022	2	Chr2:47494993	T292S	√
MSH2	rs4987188	1	Chr2:47496961	G322D	-
MSH2	rs104895023	2	Chr2:47497248	Intron	√
MSH2	rs1981929	1	Chr2:47526073	Intron	-
MSH2	rs17224444	1	Chr2:47526404	Intron	-
MSH2	rs104895024	1	Chr2:47543409	Intron	√
MSH2	rs6741393	1	Chr2:47543424	Intron	-
MSH2	rs3771278	1	Chr2:47543908	Intron	-
MSH2	rs3771279	1	Chr2:47543912	Intron	-
MSH2	rs3771280	1	Chr2:47543915	Intron	-
MSH2	rs3732182	1	Chr2:47547210	Intron	-
MSH2	rs12998837	1	Chr2:47547292	Intron	-
MSH2	rs3732183	1	Chr2:47547463	Intron	-
MSH2	rs10183143	1	Chr2:47547541	Intron	-
MSH2	rs104895025	2	Chr2:47547627	Intron	√
MSH2	rs17218363	1	Chr2:47551762	Intron	-
MSH2	rs3764959	1	Chr2:47551812	Intron	-
MSH2	rs3764960	1	Chr2:47551888	Intron	-
MSH2	rs17218439	2	Chr2:47555606	Intron	-
MSH2	rs17218446	3	Chr2:47555955	Intron	-
MSH2	rs2303428	1	Chr2:47557004	Intron	-
MSH2	rs104895026	1	Chr2:47557183	A727S	√
MSH2	rs4583514	1	Chr2:47557389	Intron	-
MSH2	rs104895027	3	Chr2:47563653	3'UTR	√
MSH2	rs17225060	1	Chr2:47563818	3'UTR	-
MSH2	rs56116962	1	Chr2:47563935	3' flanking	-
MSH2	rs104895028	1	Chr2:47563979	3' flanking	√
MSH2	rs104895029	1	Chr2:47563984	3' flanking	√
NBS1	rs14448	1	Chr8:91015009	3'UTR	-
NBS1	rs9995	1	Chr8:91015232	3'UTR	-
NBS1	rs13312986	1	Chr8:91015294	3'UTR	-
NBS1	rs1063054	1	Chr8:91015777	3'UTR	-
NBS1	rs2735383	1	Chr8:91016445	3'UTR	-
NBS1	rs104895030	1	Chr8:91016585	3'UTR	√
NBS1	rs1063053	1	Chr8:91016713	3'UTR	-
NBS1	rs13312971	1	Chr8:91018273	Intron	-
NBS1	rs13312970	1	Chr8:91018342	Intron	-
NBS1	rs13312969	1	Chr8:91018601	Intron	-
NBS1	rs7840099	1	Chr8:91024534	Intron	-
NBS1	rs3736639	2	Chr8:91024800	Intron	-
NBS1	rs1061302	1	Chr8:91027598	SYN	-
NBS1	rs2308962	1	Chr8:91027706	Intron	-
NBS1	rs104895031	1	Chr8:91034914	D527Y	√
NBS1	rs104895032	1	Chr8:91036822	L421S	√
NBS1	rs104895033	1	Chr8:91036882	P401R	√
NBS1	rs709816	1	Chr8:91036887	SYN	-
NBS1	rs1805786	1	Chr8:91037038	Intron	-
NBS1	rs1805818	1	Chr8:91040038	Intron	-

Table S4. SNPs identified by resequencing.

Gene	Variation [a]	Detection Category [b]	Position (Build 36)	Classification	Novel [c]
NBS1	rs2234744	1	Chr8:91040111	Intron	-
NBS1	rs1805824	1	Chr8:91046082	Intron	-
NBS1	rs1805826	1	Chr8:91051732	Intron	-
NBS1	rs769418	1	Chr8:91051979	Intron	-
NBS1	rs104895034	1	Chr8:91051990	Intron	√
NBS1	rs3026271	1	Chr8:91052428	Intron	-
NBS1	rs104895035	1	Chr8:91052451	Intron	√
NBS1	rs104895036	1	Chr8:91052493	Intron	√
NBS1	SWISS:VAR_025798	1	Chr8:91052651	V210F	-
NBS1	rs1805794	1	Chr8:91059655	E185Q	-
NBS1	rs104895037	1	Chr8:91059895	Intron	√
NBS1	rs61754795	1	Chr8:91062237	SYN	-
NBS1	rs1805797	1	Chr8:91063059	Intron	-
NBS1	rs104895038	1	Chr8:91063375	5' UTR	√
NBS1	rs104895039	1	Chr8:91063456	Intron	√
NBS1	rs1063045	1	Chr8:91064195	SYN	-
RAD50	rs104895040	1	Chr5:131920564	5'UTR	√
RAD50	rs104895041	1	Chr5:131920632	5'UTR	√
RAD50	rs4526098	1	Chr5:131920878	5'UTR	-
RAD50	rs104895042	3	Chr5:131923031	Intron	√
RAD50	rs74769721	1	Chr5:131939324	Intron	√
RAD50	rs104895043	1	Chr5:131942745	Intron	√
RAD50	rs104895044	1	Chr5:131943036	P165H	√
RAD50	rs17166050	1	Chr5:131943112	Intron	-
RAD50	rs75639632	1	Chr5:131943178	Intron	√
RAD50	rs2522403	1	Chr5:131943216	Intron	-
RAD50	rs28903091	1	Chr5:131951609	R327H	-
RAD50	rs104895045	1	Chr5:131952240	Intron	√
RAD50	rs104895046	1	Chr5:131952340	Q372X	√
RAD50	rs56798121	1	Chr5:131953073	Intron	-
RAD50	rs2706362	1	Chr5:131953086	Intron	-
RAD50	rs2706377	1	Chr5:131967396	Intron	-
RAD50	rs12187537	1	Chr5:131967803	Intron	-
RAD50	rs2522391	1	Chr5:131968240	Intron	-
RAD50	rs104895047	1	Chr5:131972175	Intron	√
RAD50	rs104895048	1	Chr5:131979756	Intron	√
RAD50	rs35191767	1	Chr5:131979770	Intron	-
RAD50	rs2301713	1	Chr5:131979895	Intron	-
RAD50	rs104895049	4	Chr5:131981506	Intron	√
RAD50	rs104895050	1	Chr5:131981657	Intron	√
RAD50	rs104895051	1	Chr5:131981726	R1077Q	√
RAD50	rs2074369	1	Chr5:132001562	Intron	-
RAD50	rs104895052	1	Chr5:132001904	Intron	√
RAD50	rs7737470	1	Chr5:132001962	Intron	-
RAD50	rs104895053	3	Chr5:132004328	SYN	√
RAD52	rs10849584	1	Chr12:891420	3' flanking	-
RAD52	rs56131726	1	Chr12:891449	3' flanking	-
RAD52	rs104895054	4	Chr12:891624	3'UTR	√
RAD52	rs11571487	1	Chr12:891643	3'UTR	-

Table S4. SNPs identified by resequencing.

Gene	Variation [a]	Detection Category [b]	Position (Build 36)	Classification	Novel [c]
RAD52	rs7963551	1	Chr12:891776	3'UTR	-
RAD52	rs1051672	1	Chr12:891818	3'UTR	-
RAD52	rs104895055	3	Chr12:891826	3'UTR	√
RAD52	rs11226	2	Chr12:892074	3'UTR	-
RAD52	rs11571478	2	Chr12:892173	3'UTR	-
RAD52	rs104895056	1	Chr12:892350	3'UTR	√
RAD52	rs104895057	2	Chr12:892365	3'UTR	√
RAD52	rs7310449	1	Chr12:892376	3'UTR	-
RAD52	rs104895058	1	Chr12:892423	3'UTR	√
RAD52	rs7301931	1	Chr12:892445	3'UTR	-
RAD52	rs104895059	1	Chr12:892493	3'UTR	√
RAD52	rs104895060	1	Chr12:892494	3'UTR	√
RAD52	rs104895061	1	Chr12:892496	3'UTR	√
RAD52	rs104895062	1	Chr12:892497	3'UTR	√
RAD52	rs104895063	1	Chr12:892498	3'UTR	√
RAD52	rs104895064	1	Chr12:892500	3'UTR	√
RAD52	rs104895065	1	Chr12:892501	3'UTR	√
RAD52	rs104895066	1	Chr12:892502	3'UTR	√
RAD52	rs104895067	1	Chr12:892503	3'UTR	√
RAD52	rs11571475	1	Chr12:892613	3'UTR	-
RAD52	rs1051669	1	Chr12:892713	3'UTR	-
RAD52	rs11571474	1	Chr12:892714	3'UTR	-
RAD52	rs4987208	1	Chr12:892830	3'UTR	-
RAD52	rs6413436	1	Chr12:892940	3'UTR	-
RAD52	rs4987207	1	Chr12:893479	3'UTR	-
RAD52	rs104895068	2	Chr12:893723	Intron	√
RAD52	rs28910277	1	Chr12:893931	3'UTR	-
RAD52	rs11571446	1	Chr12:906078	Intron	-
RAD52	rs7487683	1	Chr12:906303	G180R	-
RAD52	rs104895069	1	Chr12:906486	Intron	√
RAD52	rs104895070	1	Chr12:906551	Intron	√
RAD52	rs4766377	1	Chr12:909003	Intron	-
RAD52	rs11571425	1	Chr12:909027	Intron	-
RAD52	rs11571424	1	Chr12:909115	Intron	-
RAD52	rs11571420	1	Chr12:909596	Intron	-
RAD52	rs11571409	1	Chr12:910480	Intron	-
RAD52	rs104895071	4	Chr12:910558	Intron	√
RAD52	rs7303748	1	Chr12:910634	Intron	-
RAD52	rs3748522	2	Chr12:928949	Intron	-
RAD52	rs3748523	2	Chr12:928999	Intron	-

a) SNP ID from dbSNP or SwissProt where available

b) Description of detection categories:

Category	Confidence	Description
1	High	One or more PolyPhred calls at PolyPhred SNP Score 99 (regardless of local read quality)
2	High	One or more PolyPhred calls at PolyPhred SNP Score 60 **and** one or more PolyDhan calls
3	High	One or more PolyPhred calls with >= PolyPhred SNP Score 95 with average local read quality >= q30
4	High	One or more PolyPhred calls with >= PolyPhred SNP Score 90 with average local read quality >= q40
5	Low	All other calls

c) A check (√) denotes a novel SNP
