# Supplementary material for: Unique DNA Repair Gene Variations and Potential Associations with the Primary Antibody Deficiency Syndromes IgAD and CVID
Source: PLoS One. 2010 Aug 18;5(8):e12260. doi: 10.1371/journal.pone.0012260 (PMC2923613; doi:10.1371/journal.pone.0012260)
Supplement: Table S5 — (0.05 MB PDF) [file pone.0012260.s005.pdf]

Table S5. Genetic association of SNPs identified by reseqencing. Non-synonymous SNPs are depicted as the amino acid changes they encode; synonymous SNPs, SYN; Intronic SNPs, Intron; SNPs located in the 5' untranslated region, 5' UTR; SNPs located in the 3' untranslated region, 3' UTR. *p*-values <0.05 are highlighted in bold. Bold allele frequency values indicate that the SNP is unique to that group.

			Controls	IgAD		CVID	
Gene	SNP	Allele	Allele Frequency	Allele Frequency	*p*-Value	Allele Frequency	*p*-Value
APEX1	rs11622131	5' UTR	15/1892	9/650	0.18	0/226	0.18
APEX1	rs41561214	5' UTR	59/1902	18/658	0.64	3/228	0.13
APEX1	rs2307490	5' UTR	3/1872	2/642	0.46	2/230	**0.037**
APEX1	rs2307485	Intron (5)	1/1868	2/640	0.10	0/230	0.73
APEX1	rs1048945	Q51H	60/1840	12/626	0.085	9/212	0.45
MLH1	rs56198082	5' UTR	9/1904	6/658	0.20	1/230	0.94
MLH1	rs104894994	5' UTR	8/1876	6/644	0.14	1/230	0.99
MLH1	rs1799977	I219V	563/1904	187/656	0.61	79/222	0.065
MLH1	rs104894996	S247A	0/1876	2/642	**0.016**	0/230	--
MLH1	rs104895000	Q409P	0/1902	0/656	--	**1/230**	**0.0040**
MLH1	rs35001569	K618A	13/1882	5/646	0.83	2/230	0.76
MLH1	rs63750549	G638R	0/1872	**1/642**	0.088	0/230	--
MLH1	rs1800146	SYN	17/1876	10/648	0.17	2/228	0.97
MLH1	rs35831931	V716M	3/1904	1/658	0.98	1/230	0.36
MLH1	rs104895002	H727L	1/1872	0/642	0.56	1/228	0.075
MRE11	rs104895004	3' UTR	2/1866	2/636	0.26	3/228	**0.00040**
MRE11	rs104895005	3' UTR	1/1898	0/654	0.56	1/230	0.074
MRE11	rs104895007	3' UTR	2/1878	1/642	0.75	0/230	0.62
MRE11	rs13447749	3' UTR	106/1870	25/602	0.15	13/216	0.83
MRE11	rs104895010	3' UTR	0/1864	0/636	--	**1/228**	**0.0042**
MRE11	rs104895016	A492D	2/1898	3/652	0.077	1/230	0.21
MRE11	rs61749249	E494K	8/1876	0/642	0.098	2/230	0.36
MSH2	rs104895022	T292S	1/1890	2/652	0.10	0/230	0.73
MSH2	rs104895026	A727S	0/1888	**1/650**	0.088	0/230	--
MSH2	rs17225060	3' UTR	2/1892	2/646	0.26	1/228	0.21
NBS1	rs104895031	D527Y	0/1844	**1/598**	0.079	0/212	--
NBS1	rs104895032	L421S	1/1884	0/644	0.56	1/230	0.076
NBS1	rs104895033	P401R	0/1892	**2/646**	**0.016**	0/230	--
NBS1	rs61754796	V210F	4/1858	2/638	0.66	0/230	0.48
NBS1	rs1805794	E185Q	625/1892	207/650	0.58	77/224	0.69
NBS1	rs61754795	SYN	1/1872	1/642	0.43	1/230	0.077
NBS1	rs104895039	Intron	0/1814	**1/602**	0.083	0/226	--
RAD50	rs104895040	5' UTR	0/1842	**1/640**	0.090	0/230	--
RAD50	rs104895041	5' UTR	0/1900	**1/658**	0.089	0/230	--
RAD50	rs4526098	5' UTR	3/1868	3/640	0.17	0/230	0.54
RAD50	rs104895044	P165H	0/1842	**1/640**	0.0897	0/230	--
RAD50	rs28903091	R327H	4/1826	1/584	0.83	0/214	0.49
RAD50	rs104895046	Q372X	0/1900	0/654	--	**1/230**	**0.0040**
RAD50	rs104895051	R1077Q	0/1856	**1/596**	0.078	0/216	--
RAD50	rs104895053	SYN	0/1900	**1/652**	0.088	0/230	--
RAD52	rs7487683	G180R	74/1876	17/644	0.13	9/230	0.98
