# Supplementary material for: Unique DNA Repair Gene Variations and Potential Associations with the Primary Antibody Deficiency Syndromes IgAD and CVID
Source: PLoS One. 2010 Aug 18;5(8):e12260. doi: 10.1371/journal.pone.0012260 (PMC2923613; doi:10.1371/journal.pone.0012260)
Supplement: Table S6 — (0.11 MB PDF) [file pone.0012260.s006.pdf]

Table S6. Control genotype data for SNPs attempted in genotyping screen. Legend: chr:location, chromosome locations relative to human genome build 36 coordinates; % Genotyped, percent of control individuals successfully genotyped for a given marker; PredHET, predicted heterozygocity based on allele frequencies; ObsHET, observed heterozygocity; HWpval, p-value from chi-squared test of ObsHET compared to PredHET; QC pass, 303 SNPs for which >90% of samples were successfully genotyped and HWpval >0.001 are marked with a check (√).

Gene	SNP	chr:location	% Genotyped	PredHET	ObsHET	HWpval	QC pass
AID_close	rs2119089	chr12:8643499	0	-	-	-	-
AID	rs2028373	chr12:8648748	98.9	0.47	0.48	1.00	√
AID	rs2580874	chr12:8650077	100	0.48	0.42	0.35	√
AID	rs1561559	chr12:8654272	100	0.02	0.02	1.00	√
AID_close	rs714629	chr12:8657628	96.7	0.48	0.46	0.77	√
BLM	rs7184015	chr15:89075579	100	0.38	0.41	0.53	√
BLM	rs8034371	chr15:89089359	100	0.34	0.35	1.00	√
BLM	rs6496724	chr15:89090734	98.9	0.40	0.40	1.00	√
BLM	rs7183841	chr15:89095901	96.7	0.33	0.35	0.90	√
BLM	rs6496725	chr15:89096964	98.9	0.33	0.35	0.84	√
BLM	rs3784782	chr15:89103252	98.9	0.34	0.37	0.64	√
BLM	rs2518968	chr15:89108414	95.7	0.49	0.58	0.16	√
BLM	rs3784780	chr15:89110479	100	0.32	0.35	0.55	√
BLM	rs3815003	chr15:89113827	100	0.39	0.36	0.56	√
BLM	rs8036601	chr15:89116502	97.8	0.47	0.42	0.42	√
BLM	rs4932363	chr15:89124105	100	0.09	0.10	1.00	√
BLM	rs2227935	chr15:89127103	0	-	-	-	-
BLM	rs7175811	chr15:89132550	100	0.49	0.46	0.64	√
BLM	rs2229035	chr15:89138509	98.9	0.00	0.00	1.00	√
BLM	rs7182287	chr15:89145670	91.3	0.36	0.35	0.94	√
BLM	rs1801256	chr15:89148467	97.8	0.00	0.00	1.00	√
BLM	rs2270132	chr15:89152872	98.9	0.48	0.50	1.00	√
BLM	rs2073919	chr15:89154123	98.9	0.37	0.37	1.00	√
BLM	rs7167216	chr15:89155525	100	0.12	0.13	1.00	√
BLM	rs414634	chr15:89157257	98.9	0.40	0.43	0.79	√
BLM	rs374294	chr15:89158033	0	-	-	-	-
BLM_close	rs447804	chr15:89162940	0	-	-	-	-
DMC1_close	rs1946990	chr22:37242717	100	0.47	0.57	0.10	√
DMC1	rs5750616	chr22:37248840	100	0.37	0.36	0.94	√
DMC1	rs1980455	chr22:37255665	98.9	0.47	0.57	0.06	√
DMC1	rs1292811	chr22:37257832	100	0.08	0.09	1.00	√
DMC1	rs2227914	chr22:37264552	98.9	0.00	0.00	1.00	√
DMC1	rs5757130	chr22:37267740	97.8	0.48	0.60	0.025	√
DMC1	rs4821792	chr22:37272872	100	0.08	0.09	1.00	√
DMC1	rs5757133	chr22:37277781	98.9	0.47	0.50	0.75	√
DMC1	rs5757135	chr22:37282519	98.9	0.48	0.59	0.040	√
DMC1	rs5757141	chr22:37287429	96.7	0.46	0.55	0.10	√
DMC1	rs1129426	chr22:37292674	0	-	-	-	-
DMC1_close	rs8140617	chr22:37300385	100	0.45	0.53	0.14	√
DMC1_close	rs1013339	chr22:37304626	0	-	-	-	-
DMC1_close	rs1569492	chr22:37305457	98.9	0.48	0.59	0.040	√
CD3EAP,ERCC1_close	rs1046282	chr19:50602512	98.9	0.29	0.33	0.37	√
ERCC1	rs3212977	chr19:50608822	100	0.00	0.00	1.00	√
ERCC1	rs11615	chr19:50615493	100	0.44	0.45	1.00	√
ERCC1	rs3188420	chr19:50616367	0	-	-	-	-
ERCC1_close	rs1319052	chr19:50623061	96.7	0.41	0.43	1.00	√
LOC100133057,EXO1_close	rs10802995	chr1:240068905	100	0.48	0.46	0.81	√
EXO1,LOC100133057	rs1635518	chr1:240077197	100	0.50	0.52	0.84	√
EXO1	rs4149864	chr1:240082281	98.9	0.00	0.00	1.00	√
EXO1	rs1635515	chr1:240083632	97.8	0.48	0.47	0.99	√

Table S6. Control genotype data for SNPs attempted in genotyping screen. Legend: chr:location, chromosome locations relative to human genome build 36 coordinates; % Genotyped, percent of control individuals successfully genotyped for a given marker; PredHET, predicted heterozygocity based on allele frequencies; ObsHET, observed heterozygocity; HWpval, p-value from chi-squared test of ObsHET compared to PredHET; QC pass, 303 SNPs for which >90% of samples were successfully genotyped and HWpval >0.001 are marked with a check (√).

Gene	SNP	chr:location	% Genotyped	PredHET	ObsHET	HWpval	QC pass
EXO1	rs2526700	chr1:240086622	98.9	0.50	0.46	0.56	√
EXO1	rs4149910	chr1:240090581	100	0.00	0.00	1.00	√
EXO1	rs1776133	chr1:240091721	97.8	0.48	0.46	0.72	√
EXO1	rs2526698	chr1:240095724	100	0.50	0.48	0.79	√
EXO1	rs735943	chr1:240096774	98.9	0.49	0.46	0.74	√
EXO1	rs851781	chr1:240101491	100	0.50	0.44	0.27	√
EXO1	rs4149963	chr1:240102005	98.9	0.16	0.15	1.00	√
EXO1	rs4149966	chr1:240102067	100	0.01	0.01	1.00	√
EXO1	rs4149967	chr1:240102197	100	0.00	0.00	1.00	√
EXO1	rs2526697	chr1:240106752	100	0.49	0.50	1.00	√
EXO1	rs1047840	chr1:240108924	100	0.47	0.41	0.36	√
EXO1	rs12122770	chr1:240108987	100	0.03	0.03	1.00	√
EXO1	rs4149978	chr1:240109060	100	0.01	0.01	1.00	√
EXO1	rs1776148	chr1:240109168	98.9	0.48	0.44	0.49	√
EXO1	rs1635498	chr1:240111898	100	0.04	0.04	1.00	√
EXO1	rs3886571	chr1:240113323	0	-	-	-	-
EXO1	rs9350	chr1:240115297	100	0.23	0.24	1.00	√
EXO1	rs4150005	chr1:240117359	0	-	-	-	-
EXO1	rs4150018	chr1:240119244	100	0.46	0.44	0.72	√
EXO1_close	rs2526694	chr1:240124312	0	-	-	-	-
H2AFX,HMBS	rs1784304	chr11:118468026	100	0.36	0.26	0.016	√
H2AFX	rs7350	chr11:118470258	100	0.44	0.55	0.030	√
DPAGT1,H2AFX_close	rs643788	chr11:118472968	100	0.47	0.54	0.18	√
DPAGT1,H2AFX_close	rs649870	chr11:118476461	98.9	0.46	0.55	0.12	√
DPAGT1,H2AFX_close	rs3825059	chr11:118478323	0	-	-	-	-
H2AFX_close	rs4938628	chr11:118480088	0	-	-	-	-
LIG1	rs251692	chr19:53310523	0	-	-	-	-
LIG1	rs11666150	chr19:53310942	0	-	-	-	-
LIG1	rs11668325	chr19:53312774	100	0.01	0.01	1.00	√
LIG1	rs274883	chr19:53314357	100	0.23	0.20	0.34	√
LIG1	rs3731008	chr19:53318362	0	-	-	-	-
LIG1	rs156633	chr19:53319074	95.7	0.50	0.44	0.39	√
LIG1	rs3731003	chr19:53323070	100	0.00	0.00	1.00	√
LIG1	rs156641	chr19:53323220	97.8	0.48	0.44	0.54	√
LIG1	rs2288883	chr19:53328346	0	-	-	-	-
LIG1	rs3730980	chr19:53330834	0	-	-	-	-
LIG1	rs4987068	chr19:53332619	100	0.06	0.07	1.00	√
LIG1	rs3730966	chr19:53332740	0	-	-	-	-
LIG1	rs2304136	chr19:53334936	98.9	0.09	0.10	1.00	√
LIG1	rs3730947	chr19:53335082	0	-	-	-	-
LIG1	rs2288878	chr19:53338695	98.9	0.50	0.47	0.77	√
LIG1	rs3730933	chr19:53339009	100	0.02	0.02	1.00	√
LIG1	rs3730913	chr19:53344612	100	0.24	0.23	1.00	√
LIG1	rs3730911	chr19:53344862	98.9	0.00	0.00	1.00	√
LIG1	rs11879148	chr19:53345246	100	0.00	0.00	1.00	√
LIG1	rs274860	chr19:53349432	100	0.09	0.10	1.00	√
LIG1	rs12981963	chr19:53352097	100	0.00	0.00	1.00	√
LIG1	rs4987181	chr19:53356529	100	0.01	0.01	1.00	√
LIG1	rs3730862	chr19:53356610	0	-	-	-	-
LIG1,LOC374920	rs3730842	chr19:53364812	0	-	-	-	-
LOC374920,LIG1_close	rs274869	chr19:53369960	98.9	0.50	0.47	0.74	√
LOC374920,LIG1_close	rs274873	chr19:53371521	98.9	0.50	0.46	0.60	√

Table S6. Control genotype data for SNPs attempted in genotyping screen. Legend: chr:location, chromosome locations relative to human genome build 36 coordinates; % Genotyped, percent of control individuals successfully genotyped for a given marker; PredHET, predicted heterozygocity based on allele frequencies; ObsHET, observed heterozygocity; HWpval, p-value from chi-squared test of ObsHET compared to PredHET; QC pass, 303 SNPs for which >90% of samples were successfully genotyped and HWpval >0.001 are marked with a check (√).

Gene	SNP	chr:location	% Genotyped	PredHET	ObsHET	HWpval	QC pass
EPM2AIP1,MLH1_close	rs1055095	chr3:37002937	97.8	0.50	0.52	0.87	√
EPM2AIP1,MLH1	rs1800734	chr3:37009950	98.9	0.36	0.33	0.64	√
MLH1	rs9852378	chr3:37013775	100	0.49	0.50	1.00	√
MLH1	rs11541859	chr3:37017507	100	0.00	0.00	1.00	√
MLH1	rs4647224	chr3:37018234	97.8	0.49	0.48	0.90	√
MLH1	rs4234259	chr3:37023637	0	-	-	-	-
MLH1	rs4647250	chr3:37024102	96.7	0.49	0.44	0.43	√
MLH1	rs4647257	chr3:37028240	100	0.00	0.00	1.00	√
MLH1	rs1799977	chr3:37028572	0	-	-	-	-
LOC100131713,MLH1	rs1558528	chr3:37031994	97.8	0.49	0.48	0.90	√
LOC100131713,MLH1	rs4647277	chr3:37033513	0	-	-	-	-
MLH1	rs2286939	chr3:37037044	97.8	0.48	0.43	0.42	√
MLH1	rs2286940	chr3:37045110	97.8	0.49	0.49	1.00	√
MLH1	rs3774332	chr3:37049672	0	-	-	-	-
MLH1	rs9311150	chr3:37061692	100	0.00	0.00	1.00	√
MLH1	rs2241031	chr3:37065278	100	0.50	0.47	0.70	√
MLH1	rs1800149	chr3:37067062	100	0.00	0.00	1.00	√
MLH3	rs175047	chr14:74548792	96.7	0.50	0.46	0.59	√
MLH3	rs108621	chr14:74550390	0	-	-	-	-
MLH3	rs108622	chr14:74551661	100	0.50	0.47	0.66	√
MLH3	rs13712	chr14:74553565	0	-	-	-	-
MLH3	rs175052	chr14:74555159	100	0.00	0.00	1.00	√
MLH3	rs175057	chr14:74559385	100	0.50	0.44	0.28	√
MLH3	rs175062	chr14:74562047	0	-	-	-	-
MLH3	rs2098252	chr14:74562485	100	0.50	0.42	0.19	√
MLH3	rs175067	chr14:74565825	97.8	0.00	0.00	1.00	√
MLH3	rs735452	chr14:74567737	97.8	0.50	0.48	0.80	√
MLH3	rs149220	chr14:74573426	100	0.00	0.00	1.00	√
MLH3	rs3742780	chr14:74574761	97.8	0.50	0.52	0.85	√
MLH3	rs10136948	chr14:74580828	98.9	0.50	0.51	1.00	√
MLH3	rs175081	chr14:74583636	100	0.00	0.00	1.00	√
MLH3	rs175082	chr14:74587484	100	0.00	0.00	1.00	√
ACYP1,MLH3_close	rs10142770	chr14:74595202	98.9	0.50	0.51	1.00	√
MRE11	rs516037	chr11:93788460	0	-	-	-	-
MRE11	rs2155209	chr11:93790438	96.7	0.45	0.44	0.93	√
MRE11	rs1805362	chr11:93792974	98.9	0.00	0.00	1.00	√
MRE11	rs661957	chr11:93803832	97.8	0.44	0.46	0.99	√
MRE11	rs518276	chr11:93806905	100	0.47	0.54	0.23	√
MRE11	rs682213	chr11:93816167	97.8	0.50	0.62	0.031	√
MRE11	rs569143	chr11:93828035	98.9	0.49	0.57	0.18	√
MRE11	rs654718	chr11:93829763	100	0.45	0.49	0.59	√
MRE11	rs1805367	chr11:93832319	100	0.00	0.00	1.00	√
MRE11	rs680695	chr11:93851802	0	-	-	-	-
MRE11	rs1061945	chr11:93863708	100	0.00	0.00	1.00	√
ANKRD49,MRE11	rs497763	chr11:93865568	100	0.50	0.64	0.012	√
ANKRD49,MRE11_close	rs472344	chr11:93867917	97.8	0.50	0.61	0.059	√
MSH2	rs12612908	chr2:47481793	100	0.00	0.00	1.00	√
MSH2	rs1863332	chr2:47483402	97.8	0.20	0.20	1.00	√
MSH2	rs3815865	chr2:47484143	0	-	-	-	-
MSH2	rs1800151	chr2:47490943	0	-	-	-	-
MSH2	rs4987188	chr2:47496961	98.9	0.01	0.01	1.00	√
MSH2	rs4952887	chr2:47500472	100	0.14	0.15	1.00	√
MSH2	rs2347794	chr2:47510305	98.9	0.44	0.40	0.41	√

Table S6. Control genotype data for SNPs attempted in genotyping screen. Legend: chr:location, chromosome locations relative to human genome build 36 coordinates; % Genotyped, percent of control individuals successfully genotyped for a given marker; PredHET, predicted heterozygocity based on allele frequencies; ObsHET, observed heterozygocity; HWpval, p-value from chi-squared test of ObsHET compared to PredHET; QC pass, 303 SNPs for which >90% of samples were successfully genotyped and HWpval >0.001 are marked with a check (√).

Gene	SNP	chr:location	% Genotyped	PredHET	ObsHET	HWpval	QC pass
MSH2	rs3771274	chr2:47517349	100	0.45	0.39	0.31	√
MSH2	rs6726691	chr2:47523808	100	0.37	0.32	0.24	√
MSH2	rs1981928	chr2:47525979	98.9	0.38	0.34	0.40	√
MSH2	rs3771275	chr2:47528835	100	0.45	0.39	0.25	√
MSH2	rs3771276	chr2:47533677	98.9	0.44	0.41	0.61	√
MSH2	rs6729015	chr2:47540077	100	0.44	0.42	0.81	√
MSH2	rs3771278	chr2:47543908	0	-	-	-	-
MSH2	rs3771281	chr2:47545785	100	0.42	0.39	0.59	√
MSH2	rs3764959	chr2:47551812	96.7	0.39	0.37	0.81	√
MSH2	rs1800152	chr2:47555825	0	-	-	-	-
MSH2	rs2229061	chr2:47557207	0	-	-	-	-
MSH2	rs2042649	chr2:47563208	98.9	0.14	0.15	1.00	√
MSH2	rs1802577	chr2:47563570	100	0.00	0.00	1.00	√
DHFR,MSH3_close	rs844370	chr5:79978758	98.9	0.34	0.32	0.77	√
MSH3	rs380691	chr5:79987790	100	0.43	0.48	0.46	√
MSH3	rs1650670	chr5:79996605	96.7	0.35	0.27	0.066	√
MSH3	rs1650666	chr5:79998195	97.8	0.35	0.27	0.061	√
MSH3	rs1650663	chr5:79998953	97.8	0.36	0.27	0.034	√
MSH3	rs1677649	chr5:80001953	97.8	0.37	0.27	0.019	√
MSH3	rs836810	chr5:80007968	0	-	-	-	-
MSH3	rs6864493	chr5:80021458	100	0.33	0.36	0.76	√
MSH3	rs836817	chr5:80028174	0	-	-	-	-
MSH3	rs3776968	chr5:80042314	100	0.43	0.41	0.81	√
MSH3	rs245016	chr5:80056949	0	-	-	-	-
MSH3	rs3852191	chr5:80063586	0	-	-	-	-
MSH3	rs26282	chr5:80086737	100	0.39	0.26	0.0049	√
MSH3	rs33013	chr5:80095772	98.9	0.41	0.41	1.00	√
MSH3	rs6151838	chr5:80118621	100	0.19	0.19	1.00	√
MSH3	rs42290	chr5:80124778	100	0.24	0.24	1.00	√
MSH3	rs3797896	chr5:80129556	97.8	0.16	0.16	1.00	√
MSH3	rs245391	chr5:80139799	98.9	0.36	0.39	0.80	√
MSH3	rs32991	chr5:80150158	100	0.50	0.42	0.21	√
MSH3	rs26910	chr5:80156689	96.7	0.22	0.23	1.00	√
MSH3	rs3776978	chr5:80162567	100	0.20	0.19	0.64	√
MSH3	rs184967	chr5:80185737	100	0.27	0.23	0.31	√
MSH3	rs245341	chr5:80196149	96.7	0.36	0.38	0.85	√
MSH3	rs27887	chr5:80205233	98.9	0.38	0.40	0.92	√
MSH3_close	rs32964	chr5:80211553	100	0.38	0.40	0.84	√
RABGGTB,MSH4,close	rs1498311	chr1:76027314	100	0.41	0.39	0.88	√
MSH4	rs5745311	chr1:76035437	0	-	-	-	-
MSH4	rs1146646	chr1:76038088	96.7	0.35	0.27	0.066	√
MSH4	rs1146649	chr1:76041391	0	-	-	-	-
MSH4	rs5745325	chr1:76042048	98.9	0.42	0.37	0.42	√
MSH4	rs5745327	chr1:76042628	98.9	0.19	0.19	1.00	√
MSH4	rs5745329	chr1:76045310	97.8	0.00	0.00	1.00	√
MSH4	rs1146652	chr1:76046296	100	0.14	0.15	1.00	√
MSH4	rs5745353	chr1:76047725	0	-	-	-	-
MSH4	rs1144336	chr1:76050009	0	-	-	-	-
MSH4	rs1144342	chr1:76056952	98.9	0.19	0.17	0.48	√
MSH4	rs5745384	chr1:76061203	0	-	-	-	-
MSH4	rs5745390	chr1:76079179	98.9	0.33	0.26	0.10	√
MSH4	rs1565717	chr1:76093029	100	0.33	0.26	0.10	√
MSH4	rs2029682	chr1:76114207	0	-	-	-	-
MSH4	rs5745458	chr1:76118017	98.9	0.34	0.26	0.057	√

Table S6. Control genotype data for SNPs attempted in genotyping screen. Legend: chr:location, chromosome locations relative to human genome build 36 coordinates; % Genotyped, percent of control individuals successfully genotyped for a given marker; PredHET, predicted heterozygocity based on allele frequencies; ObsHET, observed heterozygocity; HWpval, p-value from chi-squared test of ObsHET compared to PredHET; QC pass, 303 SNPs for which >90% of samples were successfully genotyped and HWpval >0.001 are marked with a check (√).

Gene	SNP	chr:location	% Genotyped	PredHET	ObsHET	HWpval	QC pass
MSH4	rs5745459	chr1:76118411	100	0.07	0.05	0.22	√
MSH4	rs3819949	chr1:76126668	0	-	-	-	-
MSH4	rs1001160	chr1:76131179	98.9	0.40	0.39	0.80	√
MSH4	rs5745543	chr1:76137699	96.7	0.41	0.39	0.86	√
MSH4	rs5745545	chr1:76149944	100	0.41	0.38	0.61	√
MSH4	rs5745549	chr1:76151090	97.8	0.06	0.07	1.00	√
ASB17,MSH4_close	rs946163	chr1:76155637	100	0.41	0.39	0.88	√
CLIC1,DDAH2,MSH5_close	rs805304	chr6:31806067	98.9	0.47	0.48	0.97	√
CLIC1,MSH5_close	rs400547	chr6:31810689	100	0.10	0.09	0.45	√
MSH5	rs3131382	chr6:31815709	100	0.12	0.13	1.00	√
MSH5	rs409558	chr6:31816126	100	0.27	0.30	0.52	√
MSH5	rs2075789	chr6:31816307	0	-	-	-	-
MSH5	rs28381349	chr6:31817023	100	0.04	0.04	1.00	√
MSH5	rs707915	chr6:31818947	100	0.10	0.09	0.45	√
MSH5	rs2075788	chr6:31820160	100	0.27	0.30	0.52	√
MSH5	rs3117572	chr6:31825671	98.9	0.34	0.26	0.057	√
MSH5	rs2299851	chr6:31826581	100	0.19	0.17	0.55	√
MSH5	rs3131379	chr6:31829012	98.9	0.25	0.21	0.20	√
MSH5	rs3131378	chr6:31833264	98.9	0.25	0.20	0.15	√
MSH5	rs707939	chr6:31834667	97.8	0.41	0.43	0.76	√
MSH5	rs3117577	chr6:31835453	97.8	0.25	0.20	0.16	√
MSH5	rs3115672	chr6:31835876	0	-	-	-	-
C6orf26,MSH5	rs707938	chr6:31837338	97.8	0.46	0.46	1.00	√
C6orf26,MSH5	rs1802127	chr6:31837904	100	0.04	0.04	1.00	√
C6orf26,MSH5_close	rs707937	chr6:31838993	98.9	0.31	0.32	1.00	√
C6orf27,MSH5_close	rs707936	chr6:31841629	100	0.12	0.11	0.64	√
MSH6	rs330792	chr2:47862078	0	-	-	-	-
MSH6	rs3136245	chr2:47866350	100	0.33	0.34	1.00	√
MSH6	rs1878484	chr2:47870051	100	0.02	0.02	1.00	√
MSH6	rs1800932	chr2:47871585	97.8	0.25	0.22	0.52	√
MSH6	rs3211299	chr2:47871740	96.7	0.00	0.00	1.00	√
MSH6	rs1800935	chr2:47876619	0	-	-	-	-
MSH6	rs1800938	chr2:47879286	100	0.00	0.00	1.00	√
MSH6	rs2020908	chr2:47879812	100	0.04	0.00	0.00020	-
MSH6	rs728619	chr2:47880239	0	-	-	-	-
MSH6	rs3136334	chr2:47880493	100	0.00	0.00	1.00	√
MSH6	rs2020912	chr2:47881259	100	0.02	0.02	1.00	√
MSH6	rs2020911	chr2:47884342	100	0.45	0.38	0.19	√
FBXO11,MSH6	rs3136367	chr2:47887055	0	-	-	-	-
CFL1,MUS81_close	rs635375	chr11:65378571	96.7	0.39	0.39	1.00	√
CFL1,MUS81_close	rs652021	chr11:65379923	98.9	0.39	0.41	1.00	√
CFL1,MUS81_close	rs665306	chr11:65380601	94.6	0.44	0.48	0.61	√
MUS81	rs13817	chr11:65384910	0	-	-	-	-
MUS81	rs545500	chr11:65386510	0	-	-	-	-
EFEMP2,MUS81	rs630303	chr11:65388549	100	0.42	0.44	0.88	√
EFEMP2,MUS81	rs765593	chr11:65389308	94.6	0.00	0.00	1.00	√
EFEMP2,MUS81_close	rs659824	chr11:65393085	96.7	0.50	0.48	0.93	√
EFEMP2,MUS81_close	rs630394	chr11:65394560	98.9	0.43	0.39	0.41	√
NBS1	rs3026268	chr8:91035004	98.9	0.00	0.00	1.00	√
NBS1	rs769420	chr8:91051867	100	0.00	0.00	1.00	√
NBS1	rs1805794	chr8:91059655	96.7	0.47	0.45	0.86	√

Table S6. Control genotype data for SNPs attempted in genotyping screen. Legend: chr:location, chromosome locations relative to human genome build 36 coordinates; % Genotyped, percent of control individuals successfully genotyped for a given marker; PredHET, predicted heterozygocity based on allele frequencies; ObsHET, observed heterozygocity; HWpval, p-value from chi-squared test of ObsHET compared to PredHET; QC pass, 303 SNPs for which >90% of samples were successfully genotyped and HWpval >0.001 are marked with a check (√).

Gene	SNP	chr:location	% Genotyped	PredHET	ObsHET	HWpval	QC pass
RNF216,PMS2_close	rs710939	chr7:5657216	100	0.03	0.03	1.00	√
RNF216,PMS2_close	rs852520	chr7:5661793	100	0.50	0.53	0.71	√
RNF216,PMS2_close	rs852516	chr7:5664454	100	0.11	0.12	1.00	√
RNF216,PMS2_close	rs852417	chr7:5670195	100	0.46	0.46	1.00	√
RNF216,PMS2_close	rs852413	chr7:5675052	100	0.03	0.03	1.00	√
RNF216,PMS2_close	rs3779092	chr7:5679126	96.7	0.50	0.46	0.56	√
RNF216,PMS2_close	rs852394	chr7:5687974	95.7	0.50	0.53	0.63	√
RNF216,PMS2_close	rs4724712	chr7:5692548	100	0.50	0.57	0.28	√
RNF216,PMS2_close	rs852266	chr7:5694525	98.9	0.10	0.11	1.00	√
RNF216,PMS2_close	rs852262	chr7:5696742	100	0.02	0.02	1.00	√
RNF216,PMS2_close	rs1468996	chr7:5700983	97.8	0.47	0.46	0.94	√
PMS2	rs1805321	chr7:5993514	100	0.00	0.00	1.00	√
PMS2	rs2286680	chr7:6002033	0	-	-	-	-
JTV1,PMS2_close	rs2009115	chr7:6019082	98.9	0.14	0.15	1.00	√
JTV1,PMS2_close	rs3779107	chr7:6022455	98.9	0.14	0.15	1.00	√
JTV1,PMS2_close	rs1860459	chr7:6023145	100	0.18	0.17	1.00	√
XPO5,POLH_close	rs699937	chr6:43648904	98.9	0.41	0.37	0.54	√
POLH	rs6458343	chr6:43654356	98.9	0.10	0.11	1.00	√
POLH	rs2307456	chr6:43673546	100	0.01	0.01	1.00	√
POLH	rs9333548	chr6:43680445	100	0.00	0.00	1.00	√
POLH	rs9296419	chr6:43689563	0	-	-	-	-
POLH	rs9333555	chr6:43689913	0	-	-	-	-
POLH	rs6941583	chr6:43690069	0	-	-	-	-
RAD50_close	rs2522410	chr5:131913216	100	0.00	0.00	1.00	√
RAD50	rs4526098	chr5:131920878	100	0.00	0.00	1.00	√
RAD50	rs2244012	chr5:131929124	100	0.38	0.39	0.95	√
RAD50	rs2706348	chr5:131933709	98.9	0.37	0.39	1.00	√
RAD50	rs2230017	chr5:131943473	100	0.00	0.00	1.00	√
RAD50	rs2252775	chr5:131946343	98.9	0.37	0.39	1.00	√
RAD50	rs1047380	chr5:131958512	100	0.00	0.00	1.00	√
RAD50	rs1047382	chr5:131959284	100	0.00	0.00	1.00	√
RAD50	rs3187395	chr5:131972260	100	0.01	0.01	1.00	√
RAD50	rs1047386	chr5:131972768	100	0.00	0.00	1.00	√
RAD50	rs1047387	chr5:131979669	100	0.00	0.00	1.00	√
RAD50	rs2237060	chr5:131998784	100	0.44	0.42	0.93	√
RAD50	rs2240032	chr5:132005026	100	0.38	0.37	1.00	√
RAD50_close	rs2158177	chr5:132011957	100	0.36	0.36	1.00	√
RAD51_close	rs2412545	chr15:38766943	100	0.48	0.49	1.00	√
RAD51	rs2619681	chr15:38776313	100	0.24	0.27	0.30	√
RAD51	rs4924496	chr15:38782627	0	-	-	-	-
RAD51	rs7174493	chr15:38785766	100	0.00	0.00	1.00	√
RAD51	rs2412546	chr15:38793815	98.9	0.50	0.54	0.58	√
RAD51	rs11858337	chr15:38795572	98.9	0.50	0.54	0.61	√
RAD51	rs957603	chr15:38796960	98.9	0.48	0.53	0.48	√
RAD51	rs11070291	chr15:38803850	100	0.50	0.53	0.64	√
RAD51	rs11544205	chr15:38809068	0	-	-	-	-
RAD51	rs2229876	chr15:38809343	0	-	-	-	-
RAD51	rs1056742	chr15:38810585	100	0.00	0.00	1.00	√
FAM82A2,RAD51_close	rs4924501	chr15:38814286	100	0.50	0.57	0.28	√
FAM82A2,RAD51_close	rs11558809	chr15:38815668	97.8	0.01	0.01	1.00	√

Table S6. Control genotype data for SNPs attempted in genotyping screen. Legend: chr:location, chromosome locations relative to human genome build 36 coordinates; % Genotyped, percent of control individuals successfully genotyped for a given marker; PredHET, predicted heterozygocity based on allele frequencies; ObsHET, observed heterozygocity; HWpval, p-value from chi-squared test of ObsHET compared to PredHET; QC pass, 303 SNPs for which >90% of samples were successfully genotyped and HWpval >0.001 are marked with a check (√).

Gene	SNP	chr:location	% Genotyped	PredHET	ObsHET	HWpval	QC pass
RAD52,WNK1	rs1060499	chr12:890527	98.9	0.27	0.19	0.017	√
RAD52	rs11226	chr12:892074	0	-	-	-	-
RAD52	rs1051669	chr12:892713	97.8	0.32	0.31	0.98	√
RAD52	rs10744729	chr12:894855	98.9	0.50	0.47	0.77	√
RAD52	rs4766370	chr12:897623	98.9	0.50	0.46	0.62	√
RAD52	rs9634161	chr12:900192	100	0.26	0.20	0.061	√
RAD52	rs7312883	chr12:905165	100	0.00	0.00	1.00	√
RAD52	rs7487683	chr12:906303	100	0.03	0.03	1.00	√
RAD52	rs4766377	chr12:909003	98.9	0.33	0.31	0.67	√
RAD52	rs1131839	chr12:909294	100	0.00	0.00	1.00	√
RAD52	rs11571421	chr12:909550	0	-	-	-	-
RAD52	rs10774471	chr12:912282	0	-	-	-	-
RAD52	rs1833095	chr12:916469	100	0.39	0.39	1.00	√
RAD52	rs7311151	chr12:920515	100	0.49	0.47	0.83	√
RAD52	rs11064602	chr12:923999	0	-	-	-	-
RAD52	rs2887531	chr12:926148	100	0.40	0.39	1.00	√
RAD52	rs3748522	chr12:928949	0	-	-	-	-
RAD52_close	rs10849605	chr12:934699	96.7	0.49	0.44	0.43	√
RAD54B	rs2046666	chr8:95457324	100	0.47	0.45	0.68	√
RAD54B	rs2470740	chr8:95468727	97.8	0.48	0.44	0.59	√
RAD54B	rs2046663	chr8:95476626	100	0.16	0.13	0.26	√
RAD54B	rs3019149	chr8:95499755	100	0.38	0.36	0.73	√
LOC100128414,RAD54B	rs3136421	chr8:95514505	100	0.10	0.11	1.00	√
LOC100128414,RAD54B	rs3019279	chr8:95517081	100	0.45	0.51	0.31	√
RAD54B	rs2921385	chr8:95528524	98.9	0.48	0.47	1.00	√
RAD54B	rs2930968	chr8:95531871	100	0.45	0.48	0.82	√
RAD54B	rs1372048	chr8:95536417	100	0.39	0.38	0.95	√
RAD54B	rs2919661	chr8:95539687	100	0.00	0.00	1.00	√
RAD54B_close	rs1992371	chr8:95564238	96.7	0.47	0.49	0.78	√
SPO11_close	rs6014975	chr20:55328836	0	-	-	-	-
SPO11_close	rs12624637	chr20:55335323	0	-	-	-	-
SPO11	rs3736832	chr20:55341676	98.9	0.00	0.00	1.00	√
SPO11	rs6099553	chr20:55344526	100	0.48	0.41	0.27	√
SPO11	rs1467581	chr20:55349761	97.8	0.02	0.02	1.00	√
SPO11	rs2236330	chr20:55351421	0	-	-	-	-
TUBGCP4,TP53BP1_close	rs2244746	chr15:41482375	0	-	-	-	-
TP53BP1	rs2242067	chr15:41486595	0	-	-	-	-
TP53BP1	rs1058298	chr15:41488222	97.8	0.42	0.41	1.00	√
TP53BP1	rs11554564	chr15:41499999	100	0.00	0.00	1.00	√
TP53BP1	rs2230449	chr15:41500136	100	0.00	0.00	1.00	√
TP53BP1	rs2242069	chr15:41500926	100	0.27	0.21	0.085	√
TP53BP1	rs542898	chr15:41506684	100	0.42	0.41	0.96	√
TP53BP1	rs3803339	chr15:41511824	0	-	-	-	-
TP53BP1	rs2602141	chr15:41511938	100	0.42	0.41	0.96	√
TP53BP1	rs536313	chr15:41517290	95.7	0.41	0.39	0.78	√
TP53BP1	rs2256238	chr15:41526951	98.9	0.00	0.00	1.00	√
TP53BP1	rs2467739	chr15:41527488	97.8	0.13	0.08	0.0085	√
TP53BP1	rs2467741	chr15:41529918	98.9	0.43	0.40	0.62	√
TP53BP1	rs690367	chr15:41535596	0	-	-	-	-
TP53BP1	rs694725	chr15:41543502	98.9	0.42	0.44	0.85	√
TP53BP1	rs689647	chr15:41549488	100	0.15	0.10	0.022	√
TP53BP1	rs560191	chr15:41555066	100	0.43	0.44	1.00	√
TP53BP1	rs689754	chr15:41563187	100	0.14	0.07	0.00060	-

Table S6. Control genotype data for SNPs attempted in genotyping screen. Legend: chr:location, chromosome locations relative to human genome build 36 coordinates; % Genotyped, percent of control individuals successfully genotyped for a given marker; PredHET, predicted heterozygocity based on allele frequencies; ObsHET, observed heterozygocity; HWpval, p-value from chi-squared test of ObsHET compared to PredHET; QC pass, 303 SNPs for which >90% of samples were successfully genotyped and HWpval >0.001 are marked with a check (√).

Gene	SNP	chr:location	% Genotyped	PredHET	ObsHET	HWpval	QC pass
TP53BP1	rs2439850	chr15:41572146	98.9	0.15	0.10	0.022	√
TP53BP1	rs495175	chr15:41585976	0	-	-	-	-
TP53BP1_close	rs1869258	chr15:41590913	100	0.42	0.41	1.00	√
MAP1A,TP53BP1_close	rs523156	chr15:41599135	100	0.41	0.42	1.00	√
XRCC2_close	rs6962238	chr7:151970041	100	0.08	0.07	0.29	√
XRCC2	rs3218536	chr7:151976940	97.8	0.11	0.11	1.00	√
XRCC2	rs3111471	chr7:151993943	100	0.50	0.52	0.86	√
XRCC2	rs3218408	chr7:151998549	100	0.36	0.37	1.00	√
XRCC2_close	rs2040639	chr7:152006121	98.9	0.50	0.47	0.74	√
XRCC2_close	rs6464268	chr7:152012083	100	0.21	0.22	1.00	√
XRCC2_close	rs479215	chr7:152408312	0	-	-	-	-
XRCC2_close	rs10227264	chr7:152409893	98.9	0.30	0.24	0.11	√
XRCC2_close	rs684088	chr7:152415847	100	0.48	0.55	0.25	√
XRCC2_close	rs513586	chr7:152424532	100	0.13	0.12	0.73	√
XRCC2_close	rs2018083	chr7:152437650	98.9	0.45	0.39	0.26	√
KLC1,XRCC3_close	rs2273175	chr14:103229894	100	0.46	0.42	0.52	√
KLC1,XRCC3	rs861539	chr14:103235506	0	-	-	-	-
KLC1,XRCC3	rs709399	chr14:103237298	0	-	-	-	-
XRCC3	rs3212057	chr14:103243218	0	-	-	-	-
XRCC3	rs3212038	chr14:103247939	0	-	-	-	-
ZFYVE21,XRCC3_close	rs941474	chr14:103259614	95.7	0.50	0.51	1.00	√
