# Supplementary material for: Unique DNA Repair Gene Variations and Potential Associations with the Primary Antibody Deficiency Syndromes IgAD and CVID
Source: PLoS One. 2010 Aug 18;5(8):e12260. doi: 10.1371/journal.pone.0012260 (PMC2923613; doi:10.1371/journal.pone.0012260)
Supplement: Table S7 — (0.04 MB PDF) [file pone.0012260.s007.pdf]

Table S7. Target gene resequencing coverage by gene.

Gene	# of targets (exons)	# of 2x Covered Targets	Exons not 2x covered	Target bases	1x covered bases	2x covered bases	% 1x covered bases	% 2x covered bases	Territory bases	1x territory bases covered	2x territory bases covered	% 1x territory bases covered	% 2x territory bases covered
AID	5	5	-	2,791	2,545	2,369	91.2%	84.9%	267,936	244,258	228,141	91.2%	85.2%
APEX1	7	7	-	1,953	1,953	1,934	100.0%	99.0%	187,488	184,553	180,785	98.4%	96.4%
ERCC1	12	5	2-4, 7-9, 12	1,687	1,255	921	74.4%	54.6%	161,952	120,868	107,383	74.6%	66.3%
MLH1	19	17	10, 12	2,522	2,434	2,263	96.5%	89.7%	242,112	230,143	215,311	95.1%	88.9%
MRE11	21	19	18, 20	5,331	5,330	5,240	100.0%	98.3%	511,776	506,764	496,390	99.0%	97.0%
MSH2	16	12	2, 4, 7, 16	3,145	2,635	2,501	83.8%	79.5%	301,920	249,097	237,490	82.5%	78.7%
NBS1	17	16	1	4,671	4,398	4,325	94.2%	92.6%	448,416	415,851	405,753	92.7%	90.5%
RAD50	26	23	7, 11, 23	5,950	5,681	5,582	95.5%	93.8%	571,200	536,411	522,613	93.9%	91.5%
RAD52	18	15	1, 2, 18	4,104	3,284	3,135	80.0%	76.4%	393,984	350,473	335,790	89.0%	85.2%
